# A plant vesicle-dendritic cell chimera for enhancing cancer immunotherapy

**DOI:** 10.1038/s41467-026-73788-5

**Published:** 2026-05-28

**Authors:** Wenzhe Yi, Xindi Qian, Wenlu Yan, Dan Yan, Zhiwen Zhao, Fang Sun, Qi Zhao, Dangge Wang, Yaping Li

**Affiliations:** 1 State Key Laboratory of Drug Research & Center of Pharmaceutics, Shanghai Institute of Materia Medica, Chinese Academy of Sciences, Shanghai, China; 2 University of Chinese Academy of Sciences, Beijing, China; 3 Department of Gastroenterology, Xinhua Hospital, Shanghai Jiaotong University School of Medicine, Shanghai, China; 4 Cancer Centre, Institute of Translational Medicine, Department of Biomedical Sciences, Faculty of Health Sciences, University of Macau, Taipa, Macau SAR China; 5 Precision Research Center for Refractory Diseases, Shanghai General Hospital, Shanghai Jiao Tong University School of Medicine, Shanghai, China; 6 Shandong Laboratory of Yantai Drug Discovery & Bohai Rim Advanced Research Institute for Drug Discovery, Yantai, China

**Keywords:** Cell delivery, Biomaterials - cells, Drug delivery

## Abstract

The tumour microenvironment (TME) causes mitochondrial dysfunction in resident dendritic cells (DCs), resulting in inadequate antigen presentation and weak T cell priming. Herein, we identify hypoxia as a key factor for causing pathological mitochondrial fission in tumour-associated DCs, and develop a plant vesicle-DC chimera to relieve hypoxia-induced mitochondrial dysfunction for enhancing cancer immunotherapy. The biohybrid chimera is fabricated by loading algae-derived nanovesicles (ANVs) with genetically engineered CCR2 overexpressing-DCs. The CCR2-DC-ANVs target tumour by leveraging the C-C motif chemokine ligand 2 (CCL2) in tumours. Upon light exposure, the ANVs produce oxygen and NADPH to resolve hypoxic and oxidative stress, which reverse pathological mitochondrial fission in DCs. Mitochondrial network restoration alleviates endoplasmic reticulum stress, reduces lipid droplet accumulation, and initiates metabolic reprogramming to enhance antigen presentation and T cell priming of CCR2-DC-ANVs in the TME. The biohybrid chimera enhances therapeutic efficiency in humanized mouse models of breast cancer in female mice without requiring external tumour antigens. This approach highlights a cross-species chimera for next-generation DC therapy, and provides the basis for a nanobiotechnology platform to facilitate organelle medicine by combining photosynthesis with immunotherapy.

## Abstract (web summary)

Mitochondrial dysfunction is a cause of impaired fitness of dendritic cells (DCs) in the tumour microenvironment (TME). Here, by integrating algae-derived nanovesicles into genetically engineered DCs overexpressing CCR2, the authors show that the plant vesicle-DC chimera is sufficient to overcome DC mitochondrial dysfunction in the TME and promote antigen presentation, resulting in improved anti tumour immune responses in preclinical models.

## Introduction

An effective antitumor immune response requires the successful initiation of the cancer-immunity cycle (CIC)^1^. Dendritic cells (DCs), the most potent antigen-presenting cells (APCs), play a central role in this process by activating tumour-specific T cells through antigen presentation, co-stimulatory signalling and cytokine secretion^2–5^. However, the immunosuppressive tumour microenvironment (TME) disrupts DC function by impairing antigen presentation and preventing full activation, ultimately leading to poor T cell responses^6^. Multiple TME-driven mechanisms contribute to the dysfunction of DCs. Impaired recruitment and infiltration are major barriers. Tumour-intrinsic β-catenin signalling suppresses C-C motif chemokine ligand 4 (CCL4) production, limiting DC recruitment^7^. Although tumour-infiltrating natural killer (NK) cells recruit DCs by secreting CCL5 and XC-chemokine ligand 1 (XCL1), prostaglandin E_2_ (PGE_2_) inhibits NK cell viability and indirectly suppresses DC infiltration^8^. Low DC infiltration results in inadequate antigen-specific immune activation^9^. Furthermore, the TME induces profound mitochondrial dysfunction in DCs^10^. Previous study has shown that tumours subvert cDC1 function by modulating their mitochondria in an MST1/2-dependent manner, blunting their capacity to prime CD8^+^ T cells^11^. Dysfunctional mitochondria contribute to impaired DC motility^12^, elevated programmed cell death ligand 1 (PD-L1) expression and even cell death^13^. In addition, mitochondrial defects promote lipid accumulation in DCs, leading to lipid peroxidation^14^. The resulting by-products induce endoplasmic reticulum (ER) stress and inhibit trafficking of peptide-major histocompatibility complex (MHC) class I complexes to the cell surface^15^. Therefore, strategies that enhance intratumoural DC infiltration and restore mitochondrial fitness are critically needed to overcome TME-mediated DC suppression and potentiate antitumour immunity.

DC-based immunotherapies have been explored for the treatment of various malignancies in previous studies^16–18^. One strategy is mobilizing endogenous DCs through in vivo delivery of tumour antigens and immunomodulators^3,19–25^. However, systemic administration often suffers from rapid clearance, low bioavailability, and poor targeting, necessitating repeated dosing and increasing the risk of immune tolerance^26,27^. While intratumoural injection mitigates rapid clearance, high local concentrations of immunomodulators can induce DC dysfunction and impaired maturation^28–31^. An alternative technique involves the ex vivo generation of antigen-pulsed autologous DCs followed by reinfusion into patients^32^. Although this method allows precise DC manipulation, its clinical translation is hampered by poor migratory capacity and susceptibility to TME-mediated suppression^33,34^. Considering the crucial role of mitochondria in organelle medicine, manipulating mitochondrial homeostasis to enhance DC function has emerged as a promising strategy. Farnesyl pyrophosphate (FPP) has been reported to remodel the mitochondrial function of DCs^35^. Mitochondrial transplantation is a promising strategy for repairing mitochondrial damage, which has been successfully used in T and mesenchymal stem cells^36,37^, but the transfer rate is low (~10%)^37^. Moreover, persistent excessive oxidative stress in DCs caused by TME-induced hypoxia may diminish the effect of mitochondrial repair^38^. Inspired by recent advances in photosynthesis intervention for reprogramming mammalian cellular metabolism^39^, photosynthesis may provide a novel approach to reverse mitochondrial dysfunction in DCs. In plant photosynthetic cells, mitochondria facilitate the coordinated utilization of adenosine triphosphate (ATP), oxygen, and nicotinamide adenine dinucleotide phosphate (NADPH) generated by photosynthesis to support metabolic balance^40^. Therefore, we hypothesized that leveraging plant photosynthetic mechanisms could restore mitochondrial fitness in DCs, thereby overcoming immunosuppression and potentiating antitumour immunity.

In this study, we develop a plant vesicle-DC chimera to reverse mitochondrial dysfunction and improve antigen presentation in the TME for enhancing cancer immunotherapy. To increase tumour-specific infiltration of DCs, genetically engineered DCs overexpressing CCR2 (CCR2-DCs) are manufactured. To address TME-induced mitochondrial dysfunction and immune tolerance in DCs, algae-derived nanovesicles (ANVs) are obtained and integrated into CCR2-DCs, creating a plant vesicle-DC chimera (CCR2-DC-ANV) (Supplementary Fig. 1). Upon 670 nm light exposure, ANVs initiate photosynthetic oxygen and NADPH, simultaneously alleviating hypoxic and oxidative stress while rescuing aberrant mitochondrial fission. Enhanced mitochondrial function reduces ER stress and diminishes lipid droplet (LD) store, thereby promoting CCR2-DC-ANV survival within hypoxic TME. Mitochondrial and ER recovery further initiate metabolic reprogramming in CCR2-DC-ANVs, which in turn augmented antigen presentation and amplified T cell priming. The tumour-targeting potency and the immune-eliciting behaviours of CCR2-DC-ANVs are explored in vivo. The translational potential of CCR2-DC-ANVs has also been explored in humanized mouse models. This study highlights a promising cross-species chimera that reverses the mitochondrial dysfunction of DCs through photosynthesis, thereby enhancing the efficiency of DC-based immunotherapy.

## Results

### Progressive decline in DC infiltration and activation as tumours progress

**Fig. 1:**
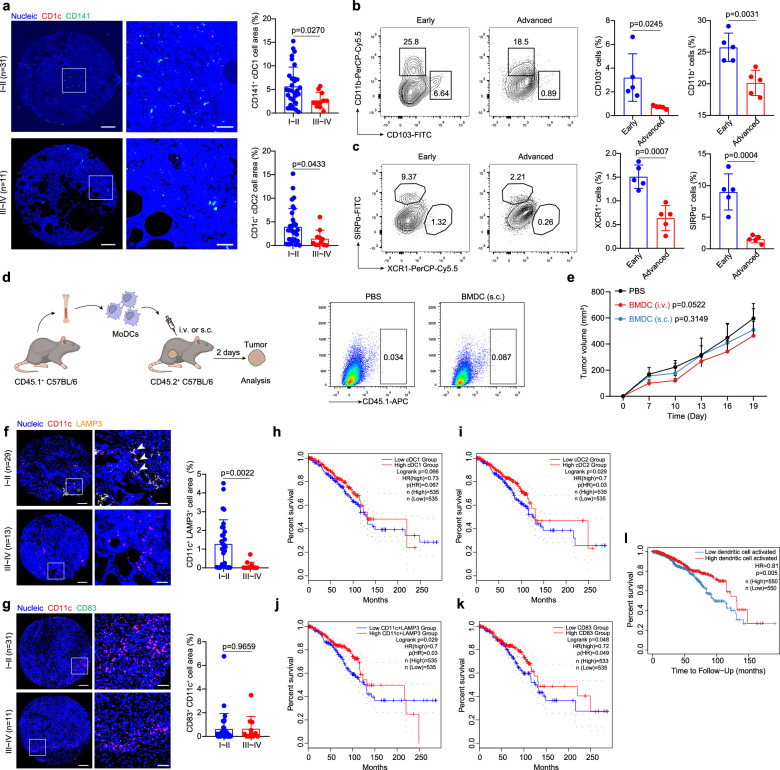
Low infiltration and activation of DCs as tumours progression. **a** Immunofluorescence staining of CD1c (cDC2s) and CD141 (cDC1s) in TMA of early (I-II)- or late (III-IV)-stage breast cancer patients and quantification of CD1c and CD141 positive area out of the total tumour area (%). *n*  =  31 (early) and *n*  =  11 (late) patients. Scale bars, 200 μm (left) and 50 μm (right). Representative flow cytometry plots and quantification of CD103^+^ and CD11b^+^ cells (gated on CD11c^+^MHC II^+^ cells) (**b**) or XCR1^+^ and SIRPα^+^ cells (gated on CD11c^+^MHC II^+^ cells) (**c**) in 4T1 tumour-bearing mice (*n* = 5). **d** Procedure to study tumour infiltration of adoptive transferred DCs and representative cytometry plots of CD45.1 BMDCs. BMDCs were generated from the bone borrow of CD45.1 mice and transferred to CD45.2 mice. **e** 4T1 tumour growth curves after adoptive transfer of BMDCs (*n* = 5). **f** Immunofluorescence staining of mature cDCs (CD11c and LAMP3) in TMA of breast cancer patients and quantification of the CD11c^+^LAMP3^+^ double-positive area out of the total tumour area (%). *n * = 29 (early) and *n * =  13 (late). Scale bars, 200 μm (left) and 50 μm (right). **g** Immunofluorescence staining of activated DCs (CD11c and CD83) in TMA of breast cancer patients and quantification of the CD11c^+^CD83^+^ double-positive area out of the total tumour area (%). *n * = 31 (early) and *n * =  11 (late). Scale bars, 200 μm (left) and 50 μm (right). Kaplan-Meier curve of the overall survival with different TPM levels of cDC1 (**h**), cDC2 (**i**), CD11c and LAMP3 (**j**) and CD83 (**k**) calculated by the GEPIA analysis of TCGA/GTEx databases. cDC1s were characterized by CD11c, CD141, XCR1 and Clec9A. cDC2s were characterized by CD11c, CD1c, CD11b and CD172a (SIRPα). **l** Kaplan–Meier curve of the overall survival with different levels of activated myeloid dendritic cell infiltration calculated by TIMER bioinformatic platform of TCGA databases. Data are mean ± s. d. Statistical analysis was evaluated with Student’s two-tailed unpaired *t-*test (**a**–**c, f**, **g**) and two-way ANOVA (**e**).

We investigated the infiltration of CD141^+^ (maker of cDC1s) and CD1c^+^ (maker of cDC2s) cells within the TME during tumour progression. Tissue microarray (TMA) analysis of human tumours revealed significantly reduced infiltration of both cells in late-stage breast cancer patients versus early-stage patients (Fig. 1a). This progressive depletion pattern was consistently observed in colorectal cancer and melanoma (Supplementary Fig. 2), indicating a pan-cancer mechanism of decreased DC infiltration following tumour progression. In parallel, we quantified tumour-infiltrating DC-like cells via gating strategy (CD11c^+^ MHC-II^+^) across murine 4T1 breast cancer, B16F10 melanoma and CT26 colorectal cancer models. We categorized tumours on day 7 post-inoculation (100 ~ 200 mm³) as early stage and those on day 12 post-inoculation (300 ~ 500 mm³) as advanced stage (Supplementary Fig. 3a, b). Decrease of CD11c^+^ MHC-II^+^ cells along with tumour progression was observed through gating CD103 versus CD11b or XCR1 versus SIRPα (Fig. 1b, c and Supplementary Fig. 3c). Given the pivotal role of DCs in antitumour immunity, we aimed to enhance intratumoural DC infiltration via adoptive DC transfer. Bone marrow-derived dendritic cells (BMDCs) were prepared using the GM-CSF/IL-4 protocol, which primarily yields monocyte-derived DCs (moDCs). The purity and function of BMDCs were also validated (Supplementary Figs. 4–7). To evaluate the tumour-homing capacity of adoptively transferred DCs from peripheral circulation, we transferred BMDCs through intravenous or subcutaneous administration (Fig. 1d). Unfortunately, both routines failed to exhibit significant tumour infiltration (Fig. 1d and Supplementary Fig. 8). Next, we evaluated the antitumour efficacy of adoptive BMDCs transfer in murine tumour models. Even with intratumoural injection, the BMDCs neither elicited detectable antitumour immune responses nor exerted tumour growth inhibition (Fig. 1 e and Supplementary Fig. 9), suggesting compromised immunocompetence of tumour-infiltrating DCs.

After that, we stained mature DCs that may have captured tumour antigens using typical DC markers CD11c and LAMP3^41,42^. The dual staining showed a low level of mature DCs in late-stage tumours compared with early-stage tumours in patients (Fig. 1f and Supplementary Fig. 10a, b). Besides, we observed that CD11c^+^CD83^+^ DCs remained chronically low regardless of tumour stage (Fig. 1g and Supplementary Fig. 10c, d). CD83 serves as a hallmark of DC activation and is highly expressed on mature DCs^43^ (Supplementary Fig. 10e). Critically, CD83 stabilizes MHC-II and CD86 expression on the cell membrane by inhibiting their endocytic degradation^44^. Gene Expression Profiling Interactive Analysis (GEPIA, based on the Cancer Genome Atlas/Genotype-Tissue Expression (TCGA/GTEx) cohorts) and Tumour Immune Estimation Resource (TIMER, based on the TCGA cohorts) database analysis confirmed that cDC1/cDC2 infiltration, LAMP3/CD83 expression and activated DC density significantly correlated with prognostic and survival of breast cancer patients (Fig. 1h-l). Taken together, it demonstrated consistent decline of DCs coupled with suppressed maturation across human samples and murine tumour models.

### Mitochondrial fragmentation and dysfunction in TADCs

To investigate the biological alterations in TADCs, we purified DC populations from peripheral blood, spleen and tumour tissues of tumour-bearing mice, followed by subcellular structure examination via transmission electron microscopy (TEM) (Supplementary Fig. 11). Small and fragmented mitochondria (red arrows) were observed in TADCs, contrasting with the large and tubular mitochondria in peripheral/splenic DCs (Supplementary Fig. 12a). Besides, we observed ER stress-induced vacuolization and LD accumulation in TADCs, which were downstream manifestations of mitochondrial damage^45,46^. Notably, mitochondrial mass was reduced in TADCs, accompanied by declined mitochondrial membrane potential and elevated mitochondrial reactive oxygen species (ROS) levels (Supplementary Fig. 12b–e), suggesting mitochondrial fragmentation as a hallmark of TADCs. Mitochondrial fragmentation impairs functional execution and increases electron leakage during oxidative phosphorylation (OXPHOS). Seahorse XF Mito Stress assays revealed TADCs exhibited lower basal oxygen consumption rates (OCR) and maximal respiration versus peripheral/splenic DCs (Supplementary Fig. 12f), suggesting the impaired metabolic outputs in TADCs. Besides, MHC-I-peptide complex staining revealed significantly impaired antigen presentation in TADCs (Supplementary Fig. 12g). We next profiled mitochondrial functions and TADC functional states at the transcriptomic level. We found that DCs maintained normal mitochondrial mass at 5% O₂, but exhibited a significant and progressive loss of mitochondrial mass at both 1% and 0.1% O₂ for over 4 h, confirming that hypoxia induced mitochondrial damage (Supplementary Fig. 13). Therefore, BMDCs were co-cultured with murine triple negative breast cancer (TNBC) cells under hypoxia (Supplementary Fig. 14a). Gene ontology (GO) annotation analysis showed that TME-induced organelle dysfunction in DCs (Supplementary Fig. 14b). Gene set enrichment analysis (GSEA) revealed down-regulated mitochondrial function (Supplementary Fig. 14c). Furthermore, mitochondrial fission-related genes, such as *FIS1, Bax, Ddhd1* and *Gabarapl1*, increased in TADCs (Supplementary Fig. 14d), which drove mitochondria into discrete fragments. Taken together, TADCs exhibited fragmented mitochondrial networks, deficient respiratory and impaired antigen presentation. DCs demonstrate inherently limited tumour-homing capacity along with tumour progression. Even upon successful infiltration, those rare TADCs acquire pathological mitochondrial fragmentation, which drives their transition into an immunosuppressive state that fails to elicit effective anti-tumour immunity (Supplementary Fig. 14e).

To investigate the cause of mitochondrial fragmentation in TADCs, we found significant enrichment of hypoxia-associated gene signatures in BMDCs co-cultured with tumour cells under hypoxia (Supplementary Fig. 15a). Mitochondrial function profiling revealed that hypoxia was a key inducer of DC mitochondrial dysfunction (Supplementary Fig. 15b–d). Both TEM and confocal imaging confirmed significant hypoxia-induced mitochondrial fragmentation (Supplementary Fig. 15e–g). Consistently, hypoxia reduced basal OCR and maximum respiration (Supplementary Fig. 15h, i), indicating impaired respiratory function. Besides, hypoxic stress suppressed DC activation markers (CD40/83) and antigen presentation (Supplementary Fig. 15j, k). Drp1 serves as the main regulator of mitochondrial fission, executing division at designated mitochondrial constriction sites^47,48^. Phosphorylation of Drp1^Ser616^ translocated to the mitochondrial outer membrane, assembling into multimeric spirals that drive membrane constriction and scission^49^. Flow cytometry analysis and confocal imaging showed abundant cytoplasmic accumulation of Drp1^ser616^ in hypoxic DCs Supplementary Fig. 15l, m). Genetic Drp1 knockdown rescued mitochondrial morphology in hypoxic DCs, demonstrating that hypoxia-induced mitochondrial fission in DCs was associated with the function of Drp1^ser616^ (Supplementary Fig. 15n, o).

### Fabrication and characterization of the plant vesicle-DC chimera

Recruitment and localization of immune cells in TME depends on migratory guidance signals of chemokines and corresponding receptors^50^. CCL2, known as monocyte chemoattractant protein-1 (MCP-1), is a key molecule to mediate monocyte infiltration into TME and facilitates cancer progression^51^. In murine models, CCL2 levels were significantly higher in different tumour types compared to non-tumour organs (heart, liver, spleen, lung and kidney) (Supplementary Fig. 16a). To validate the potential of CCL2 to recruit DCs, we performed correlative analyses using the TIMER databases, revealing a positive correlation between tumour CCL2 levels and DC infiltration (Supplementary Fig. 16b). Next, we evaluated CCL2 receptor (CCR2) expression on different lymphocytes and monocytes. High levels of CCR2 were detected in neutrophils and macrophages, whereas low levels were observed in DCs, T cells, B cells and natural killer (NK) cells (Supplementary Figs. 17 and 18). The CCL2/CCR2 axis has been proven to promote tumour tropism of diverse immune cells and leveraged to enhance tumour targeting in engineered macrophages and CAR-T therapies^52–54^. Therefore, genetic CCR2 overexpression would enhance the tumour-homing efficiency of DCs.

**Fig. 2:**
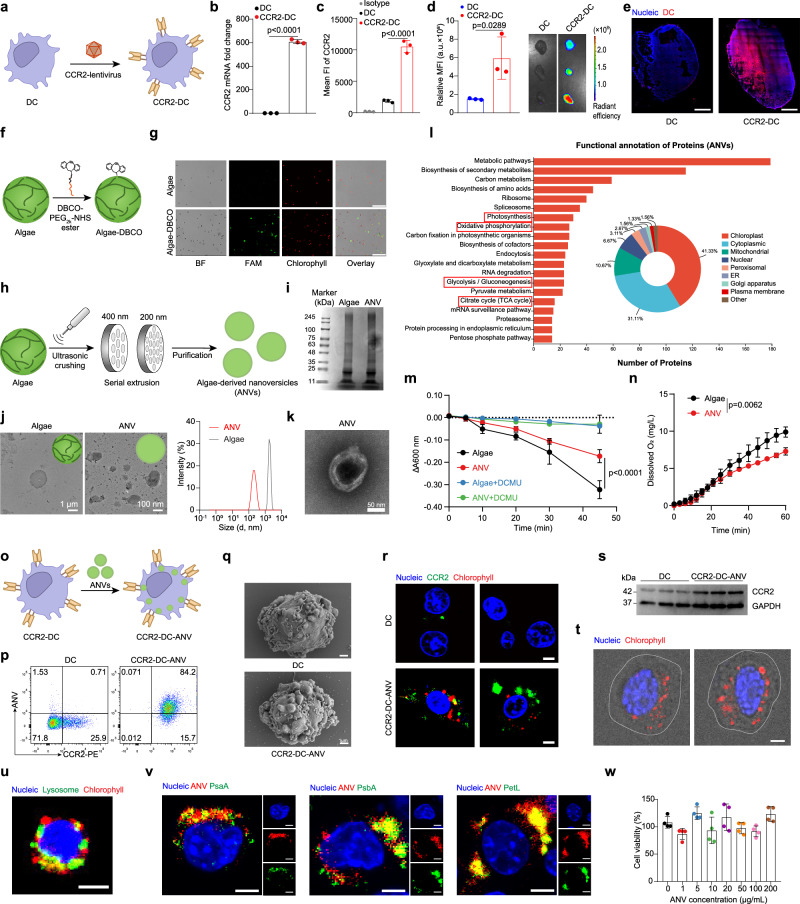
Fabrication and characterization of CCR2-DC-ANVs. **a** Scheme illustration of the preparation of CCR2-DC. RT-qPCR (**b**) and cytometry (**c**) analysis of CCR2 expression (*n* = 3). **d** Quantitative analysis and representative image of fluorescence in tumours after CCR2-DC administration (*n* = 3). DCs were labelled by DiR (red). **e** Representative immunofluorescence images of CCR2-DCs in tumour (*n*  =  3). Scar bar, 500 μm. **f** Scheme illustration of the preparation of algae-DBCO. **g** Representative immunofluorescence images of algae-DBCO (*n*  =  3). Algae-DBCO were labelled by FAM-N_3_ (green). Scar bar, 50 μm. **h** Scheme illustration of the preparation of algae-derived nanovesicles (ANVs). **i** Sodium dodecyl sulfate–polyacrylamide gel electrophoresis (SDS–PAGE) pattern of proteins from algae and ANVs (*n*  =  3). **j** Transmission electron microscopy (TEM) images and hydrodynamic size of algae and ANVs (*n*  =  3). Scar bar, 1 μm (left) and 100 nm (right). **k** Cyro-EM images of ANVs (*n*  =  3). Scale bar, 50 nm. **l** Proteomics analysis of ANVs. The identified proteins were classified according to their cellular components and biological processes (*n*  =  3)**. m** Absorption changes of DCPIP at 600 nm after treatment with algae and ANVs under red light irradiation (*n*  =  3). **n** Dissolved oxygen content of algae and ANVs under red light irradiation (*n*  =  3). **o** Scheme illustration of the preparation of CCR2-DC-ANVs. **p** Flow cytometry analysis of CCR2-DC-ANVs (*n*  =  3). **q** Scanning electron microscopy images of BMDC and CCR2-DC-ANVs (*n*  =  3). Scale bar, 1 μm. **r** Representative immunofluorescence images of CCR2-DC-ANVs (*n*  =  3). CCR2 was labelled by a secondary antibody (green) and ANVs were labelled by chlorophyll (red). Scale bar, 2.5 μm. **s** Western blotting analysis of CCR2 and GAPDH expression in BMDCs and CCR2-DC-ANVs (*n*  =  3). **t** Bright-field image of CCR2-DC-ANVs (*n*  =  3). The edge of the cell was labelled by white line. Scale bar, 2.5 μm. **u** Fluorescent visualization of ANVs localization in BMDCs 6 h after incubation (*n*  =  3). Scale bar, 5 μm. **v** Fluorescent visualization of key photosynthetic proteins (PsaA, PsbA, and PetL) in CCR2-DC-ANVs 8 h after incubation (*n*  =  3) (ANVs, red; nuclei, blue; photosynthetic proteins, green). Scale bar, 2.5 μm. **w** Cell viability of CCR2-DC-ANVs via CCK-8 assay. (*n* = 4). Data are mean ± s. d. Statistical analysis was evaluated with student’s two-tailed unpaired *t-*test (**b**, **d**) and one-way ANOVA (**c**).

Consistent with the above results, CCR2-overexpressed BMDCs (CCR2-DCs) were genetically engineered for testing the tropism towards tumours. The CCR2-DCs were prepared using a lentiviral vector (HBLV-m-CCR2-3xflag-ZsGreen-PURO) (Fig. 2a). CCR2 expression in BMDCs was validated by quantitative real-time PCR (qRT-PCR) and flow cytometry (Fig. 2b, c). Subsequently, DiR-labelled BMDCs and CCR2-DCs were administered subcutaneously. Results demonstrated that CCR2-DCs exhibited enhanced tumour-homing efficiency versus BMDCs (Fig. 2d). Fluorescence signals further confirmed the tropism migration of CCR2-DCs (Fig. 2e). To comprehensively clarify the role of CCR2 in mediating recruitment of CCR2-DCs, we employed *Ccl2*^*−/−*^ mice and *Ccl2* knockout 4T1 cell lines to establish tumour-bearing mouse models. CCR2-DCs accumulation was decreased in both *Ccl2* knockout 4T1-bearing wild type (WT) mice and 4T1-bearing *Ccl2*^*−/−*^ mice when compared with 4T1-bearing WT mice. A similar decrease in accumulation was observed in *Ccl2* knockout 4T1-bearing *Ccl2*^*−/−*^ mice. (Supplementary Fig. 19). Overall, the CCR2 expression would benefit the tropism migration of CCR2-DCs to tumours via CCR2-CCL2 axis.

Next, we obtained algae-derived nanovesicles (ANVs) by ultrasonic crushing. The algae (GY-D12 *Chlorella pyrenoidosa)* were cultivated in a tris-acetate-phosphate (TAP) medium. First, the algae were conjugated with DBCO-PEG_2k_-NHS ester (Fig. 2f). The optimal DBCO-PEG_2k_-NHS ester concentration was determined by flow cytometry (Supplementary Fig. 20). The DBCO-modification of algae was confirmed (Fig. 2g), and long-lasting DBCO modification was detected (Supplementary Fig. 21). Then, ANVs were generated as shown in Fig. 2h. The maintained proteins in ANVs and algae were comparable (Fig. 2i). The ANVs were characterized by TEM and dynamic light scattering (DLS), verifying the nanosized vesicle structure (Fig. 2j and Supplementary Fig. 22). Cryo-Electron Microscopy (Cryo-EM) confirmed vesicular morphology of ANVs (Fig. 2k). Proteomics results showed that the ANVs retained almost all the protein components required for photosynthesis (Fig. 2l). GO analysis indicated that ~41.3% proteins belonged to chloroplast. Functional annotation analysis suggested that the ANV components were involved in photosynthesis. Successful photosynthesis requires a complete electron transport chain (ETC). To validate the photosynthesis capability of ANVs, we added the artificial electron acceptor 2,6-dichlorophenolindophenol (DCPIP) to suspensions of both ANVs and algae, followed by light exposure. A decrease in the absorbance of DCPIP at 600 nm was detected in both suspensions (Fig. 2m). The ANVs, however, signalled a weaker electron-accepting capacity relative to the algae. The ETC process was progressively enhanced with prolonged illumination but was effectively abolished by the ETC inhibitor 3-(3,4-dichlorophenyl)-1,1-dimethylurea (DCMU) (Fig. 2m). Moreover, the oxygen production efficiency of ANV was also evaluated, proving the realization of photosynthesis via ANVs (Fig. 2n).

To fabricate the plant vesicle-DC chimera (CCR2-DC-ANV), Ac_4_ManNAz was incubated with CCR2-DCs to generate azido groups on cell membrane, and then DBCO-modified ANVs were incubated with the azido-modified CCR2-DCs (Fig. 2o). Approximately 84.3 % of BMDCs were modified with ANVs and almost all of them were CCR2^hi^ DCs (Fig. 2p). Similar spherical morphologies of BMDCs and CCR2-DC-ANVs were observed (Fig. 2q). The successful fabrication of CCR2-DC-ANVs was further confirmed using fluorescent staining (Fig. 2r and Supplementary Fig. 23). Obvious signals from chlorophyll on ANVs and CCR2 were detected. The CCR2 expression on CCR2-DC-ANVs was evaluated using western blot (Fig. 2s). Bright-field microscopy images of CCR2-DC-ANVs confirmed that ANVs exhibited exclusive intracellular localization without membrane integration (Fig. 2t). Quantitative analysis demonstrated that ANVs derived from DBCO-modified algae achieved a high conjugation efficiency of approximately 100 μg ANVs per million DCs (Supplementary Fig. 24). Flow cytometry confirmed unaltered surface CCR2 expression in CCR2-DC, CCR2-DC-N_3_ and CCR2-DC-ANVs (Supplementary Fig. 25). Besides, CCR2-DC-ANVs retained full CCL2-binding capacity (~98.7%) (Supplementary Fig. 25). To evaluate ANV internalization, we tracked subcellular localization of ANVs. After 6 h incubation, chlorophyll signals predominantly accumulated in the cytoplasm rather than cell membrane (Supplementary Fig. 26). Endocytosis blockade experiments demonstrated that ANVs were internalized through scavenger receptor class A- and clathrin-mediated endocytosis. Foreign substances internalized by endocytosis are mainly transported to the lysosome^55^, but we observed significant signals of chlorophyll in cytoplasm (Fig. 2u and Supplementary Fig. 27). This may attribute to the contents of ANVs, such as cytoderm or protoplast membrane fragments, which facilitate the rapid escape of ANVs from endosomes. Moreover, the co-localization of chlorophyll signals with key photosynthetic proteins (PsaA, PsbA, and PetL) in cytoplasm demonstrated that the photosynthetic proteins remained stay with the thylakoid membrane structure, maintaining their spatial localization to ensure photosynthetic functionality (Fig. 2v). The cell viability was not affected by ANV incubation and ~85 % of cells persisted ANV signals after 48 h (Fig. 2w and Supplementary Fig. 28). Moreover, the internalized ANVs did not increase the intracellular ROS levels of CCR2-DC-ANVs (Supplementary Fig. 29).

### Reversal of mitochondrial fragmentation in CCR2-DC-ANVs upon light exposure

**Fig. 3:**
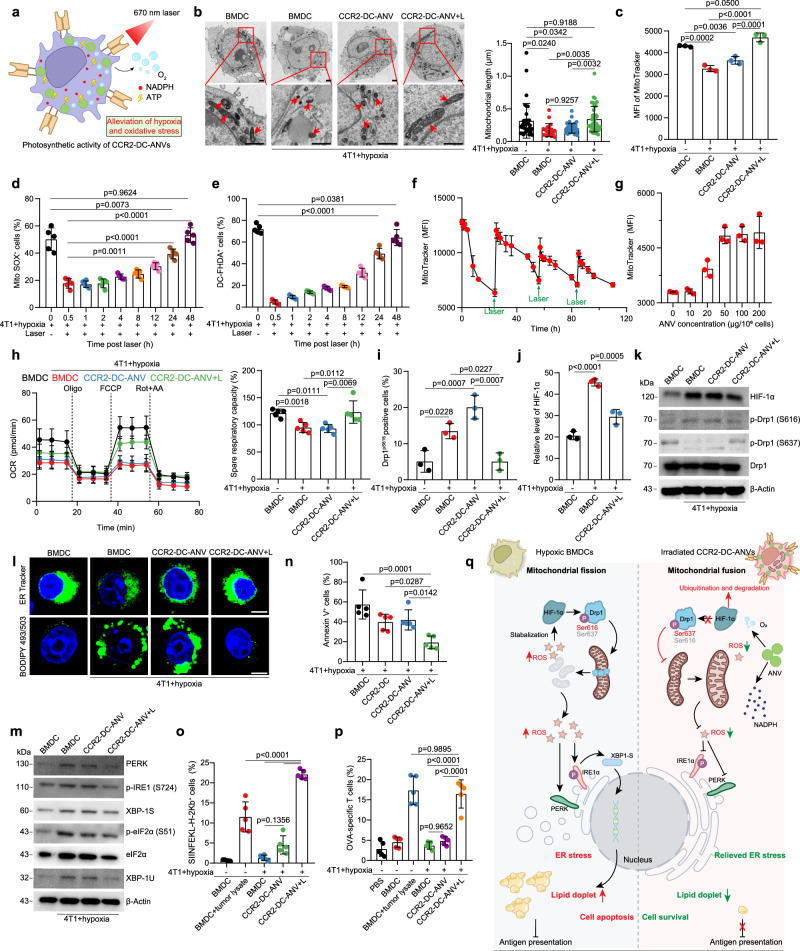
Reversal of mitochondrial fragmentation in CCR2-DC-ANVs upon light exposure. **a** Scheme illustration of ATP, NADPH and oxygen generation of CCR2-DC-ANVs. **b** Transmission electron microscopy images showing the mitochondrial morphology of DCs (*n*  =  3). The mitochondria were labelled by red arrows. Scale bar, 1 μm. Mitochondrial lengths were calculated from one cell in each group. **c** Quantification of MitoTracker in different groups (*n*  =  3). **d** Quantitative analysis of MitoSOX in CCR2-DC-ANVs at different time points post-irradiation (*n* = 5). **e** Quantitative analysis of intracellular reactive oxygen species (ROS) in CCR2-DC-ANVs at different time points post-irradiation (n = 5). **f** Quantitative analysis of MitoTracker at different time points. CCR2-DC-ANVs were cultured in hypoxia condition and received light exposure (670 nm, 10 min) at 24, 56 and 84 h (*n* = 3). **g** Quantitative analysis of MitoTracker after CCR2-DC-ANVs (loaded with different amounts of ANVs) were exposed to 670 nm light (*n* = 3). **h** Oxygen consumption rate (OCR) analysis of CCR2-DC-ANVs and the calculated spare respiratory capacity (*n* = 5). **i** Quantitative analysis of Drp1 phosphorylated at Ser616 (Drp1^pS616^) in different groups by flow cytometry (*n* = 3). **j** Relative levels of HIF-1α gene expression in different groups via transcriptomic analysis (n = 3). **k** Western blotting analysis of Drp1^pS616^, Drp1^pS637^ and HIF-1α in different groups (*n*  =  3). **l** Representative immunofluorescence images of ER Tracker and BODIPY 493/503 in different groups (*n*  =  3). Scale bar, 5 μm. **m** Western blotting analysis of PERK, e-IF2α^pS51^, IRE1α^pS724^ and XBP1 in different groups (*n*  =  3). **n** Quantification of apoptosis (annexin V^+^) in tumour-infiltrated CCR2-DC-ANVs after adaptive transfection (*n*  =  5). **o** Quantitative analysis of SIINFEKL-H-2Kb^+^ cells in different groups (*n* = 5). **p** Quantitative analysis of OVA-specific CD8^+^ T cells in different groups (*n* = 5). **q** Scheme illustration of repairment of mitochondrial dysfunction in irradiated CCR2-DC-ANVs. The oxygen and NADPH generated by ANVs alleviated hypoxic stress and scavenged intracellular ROS, thereby suppressing HIF-1α signalling-induced Drp1^pS616^ and ultimately promoting mitochondrial fusion in irradiated CCR2-DC-ANVs. Mitochondrial morphological restoration combined with reduced intracellular ROS levels suppresses PERK activation and eIF2α phosphorylation-induced ER stress and XBP1 activation-driven lipid droplet (LD) accumulation, ultimately prolonging cell survival and enhancing antigen presentation of irradiated CCR2-DC-ANVs. Data are mean ± s. d. Statistical analysis was evaluated with one-way ANOVA (**b**–**e**, **h**–**j**, **n**–**p**).

Given the successful fabrication of CCR2-DC-ANVs, the photosynthetic activity was assessed (Fig. 3a). Upon exposure to the 670 nm laser (600 mW/cm^2^), the intracellular ATP and NADPH levels gradually increased (Supplementary Fig. 4a–c). Fluorescence signal of oxygen probe ([Ru(dpp3)] Cl2) confirmed the intracellular oxygen generation of CCR2-DC-ANVs under hypoxia (Supplementary Figs. 30–32). Dissolved oxygen measurements revealed that CCR2-DC-ANVs performed higher oxygen yields compared to BMDCs, but lower oxygen yields than ANVs (Supplementary Figs. 33 and 34). Next, we evaluated the photosynthetic activity of CCR2-DC-ANVs under hypoxic TME. Upon irradiation, CCR2-DC-ANVs exhibited significantly increased ATP production and NADPH levels (Supplementary Fig. 35a, b). The intracellular oxygen level of CCR2-DC-ANVs was also confirmed (Supplementary Figs. 35c and 36). These findings demonstrated that the ANVs-mediated photosynthetic activity boosted intracellular NADPH and oxygen levels to relieve hypoxia stress.

To assess the effect of photosynthetic on mitochondria in CCR2-DC-ANVs, we co-cultured CCR2-DC-ANVs with murine TNBC cells under hypoxia. TEM imaging confirmed restoration of mitochondrial morphology in CCR2-DC-ANVs after light exposure (Fig. 3b). Increased mitochondria mass (Fig. 3c), enhanced mitochondrial membrane potential, reduced mitochondrial ROS level, remobilized mitochondria and cytoplasmic calcium ions were detected in irradiated CCR2-DC-ANVs (Supplementary Fig. 37). Light-stimulation failed to trigger photosynthetic effects or mitochondrial remodelling in BMDCs, confirming light-responsiveness is exclusive to CCR2-DC-ANVs (Supplementary Fig. 38). Upon 10-min light irradiation, CCR2-DC-ANVs maintained physiological oxygen levels for over 12 h (Supplementary Fig. 39, 40). Both mitochondrial and cytosolic ROS levels showed negligible increase during the period (Fig. 3d, e). Even at 24 h post-irradiation, ROS levels were lower than the unirradiated controls. In addition, mitochondrial content in CCR2-DC-ANVs was rapidly restored to normal levels within 1 h after 10-min light exposure, and was maintained at a high level in the following 24 h (Fig. 3f). Moreover, repeated light exposures enabled sustained activation of mitochondrial fusion process. Dose-correlation studies showed that 50 μg ANVs/10⁶ cells with 10-min light exposure effectively restored mitochondrial mass. CCR2-DC-ANVs (100 μg ANVs/10⁶ cells) performed a surplus of oxygen generation and mitochondrial repair capability (Fig. 3g). Thus, 10-min irradiation was sufficient and optimal for mitochondrial functional recovery. Notably, CCR2-DC-ANVs with 10-min irradiation achieved normalized basal OCR and exhibited higher maximum respiration than hypoxic BMDCs (Fig. 3h and Supplementary Fig. 41). GSEA showed that mitochondrial, respiratory chain complex and ATP synthesis-related gene expression was significantly upregulated due to the light-mediated mitochondrial fusion in CCR2-DC-ANVs (Supplementary Fig. 42). The restored level of respiratory chain complex components (NDUFB8, SDHB and ATP5A1) further confirmed the recovered mitochondrial morphology and function in CCR2-DC-ANVs (Supplementary Fig. 43).

Given the critical role of Drp1^ser616^ in hypoxia-induced mitochondrial fragmentation, we evaluated its status in CCR2-DC-ANVs post-irradiation. Notably, we observed a decreased Drp1^ser616^ phosphorylation and increased Drp1^ser637^ phosphorylation (Fig. 3i-k), Drp1^ser637^ was the inhibitory site of Drp1^56^. We also found an increase of mitochondrial fusion proteins, including mitofusin-1 (Mfn-1), Mfn-2 and optic atrophy protein 1 (OPA1) at the gene and protein levels^57^ (Supplementary Fig. 44). Transcriptional profiles revealed that irradiated CCR2-DC-ANVs significantly downregulated hypoxia response genes, such as *HIF1α*, which enhanced phosphorylation of Drp1 at ser616 site^58–60^ (Fig. 3j). Western blotting validated the decreased level of HIF-1α proteins in irradiated CCR2-DC-ANVs (Fig. 3k).

To further interrogate the mechanisms between photosynthetic activity and mitochondrial fusion in CCR2-DC-ANVs, we treated algae with Simazine (Photosystems I (PSI) inhibitor) or Diquate (Photosystems II (PSII) inhibitor) when preparing CCR2-DC-ANV. PSI and PSII operate synergistically through an interconnected electron transport chain, driving the light-dependent reactions of photosynthesis to generate oxygen and NADPH^61,62^ (Supplementary Fig. 45a). Both PSI and PSII are indispensable for functional photosynthesis. Inhibiting PSI or PSII prevented irradiated CCR2-DC-ANVs from restoring mitochondrial mass, membrane potential and ROS homeostasis, while also blocking recovery of basal and maximal respiratory capacity (Supplementary Fig. 45b-e). Confocal imaging confirmed that mitochondrial fragmentation, ER stress and LD accumulation were not well repaired after PSI or PSII inhibition (Supplementary Fig. 45f). Taken together, the 10-min light exposure improves the function of CCR2-DC-ANVs through following mechanisms. The photosynthetic activity of ANVs elevated intracellular oxygen and NADPH levels, which alleviated intracellular hypoxia and ROS stress. It facilitated HIF-1α degradation, and inhibited Drp1^Ser616^ phosphorylation while promoted Drp1^Ser637^ phosphorylation to shift mitochondrial state from fission to fusion, thereby restoring its morphology and function.

### Mitochondrial fusion in CCR2-DC-ANVs relieves ER stress to promote cell survival and antigen presentation

Given that photoactivated CCR2-DC-ANVs elevated intracellular O₂ and NADPH to restore mitochondrial function while suppressing ROS, we proposed that this intervention might relieve ER stress and induce LD degradation. Both TEM and confocal imaging demonstrated that hypoxia induced pathological ER vacuolization in BMDCs, while light exposure restored structured ER sheet formation in CCR2-DC-ANVs (Fig. 3l and Supplementary Fig. 46a). Quantification of triacylglycerol (TAG) levels and BODIPY fluorescence signal confirmed that light irradiation significantly reduced LD accumulation in CCR2-DC-ANVs (Supplementary Fig. 46b, c). The sustained activation of PERK-phospho-eIF2α axis at ER-mitochondria contact sites drove unresolved ER stress^63^. The aberrant stress further triggered IRE1α-XBP1 signalling, leading to pathological LD accumulation in TADCs by upregulating triglyceride biosynthesis^15^. Indeed, we observed that hypoxia increased the ER stress proteins PERK and phospho-eIF2α in BMDCs and CCR2-DC-ANVs, which was resolved by light exposure (Fig. 3m). Furthermore, irradiated CCR2-DC-ANVs exhibited significant downregulation of the IRE1α-XBP1 axis, evidenced by reduced phospho-IRE1α levels and diminished XBP1 splicing (Fig. 3m). Therefore, light-driven photosynthesis in CCR2-DC-ANVs relieved ER stress, suppressing pathological LD accumulation.

Pathological mitochondrial fragmentation provokes aberrant ROS leakage, driving unresolved ER stress that induces cell apoptosis. We found that TADCs showed higher levels of apoptosis than peripheral DCs and splenic DCs (Supplementary Fig. 47). During hypoxic culture in vitro, we also observed that the genes with increased expression were enriched for apoptosis signatures (Supplementary Fig. 48). Chimerism of ANVs could not affect the apoptosis type of CCR2-DC-ANVs, whereas irradiated CCR2-DC-ANVs rescued the pro-apoptotic effect (Supplementary Fig. 49a, b). Further investigations revealed that irradiated CCR2-DC-ANVs maintained low apoptosis rates under hypoxic culture for 72 h (Supplementary Fig. 49c). In contrast, BMDCs exhibited progressively increasing apoptosis, demonstrating that light exposure prolonged CCR2-DC-ANV survival under hypoxic conditions. RNA-seq analysis revealed significant downregulation of key apoptosis genes in irradiated CCR2-DC-ANVs, including apoptosis genes *Casp3*, *Casp6*, *Casp7* and *Casp8* (Supplementary Fig. 50a). Caspase-3 serves as an executioner protease in apoptosis, where its cleavage status directly reflects apoptotic flux. We observed that hypoxia plus tumour-conditioned medium increased caspase-3/7 activation in BMDCs, which was significantly reduced in irradiated CCR2-DC-ANVs (Supplementary Fig. 50b). To assess in vivo survival, CellTracker Blue-labelled CCR2-DC-ANVs were adoptively transferred into tumour-bearing mice (Supplementary Fig. 51). Flow cytometry analysis at 24 h post-irradiation demonstrated a decreased levels of apoptosis in irradiated CCR2-DC-ANVs (Fig. 3n). Together, light-driven mitochondrial metabolism contributes to CCR2-DC-ANV survival within TME.

The pathological LD accumulation in TADCs impaired antigen presentation capacity^15^. Given the reduced LD level in irradiated CCR2-DC-ANVs, we assessed the antigen presentation. Irradiated CCR2-DC-ANVs exhibited higher MHC-I-peptide complex expression and enhanced antigen-specific T cell activation (Fig. 3o, p). Extending the irradiation duration on the basis of 10 min does not significantly improve antigen presentation (Supplementary Fig. 52a). Furthermore, CCR2-DC-ANVs maintained durable antigen presentation over extended durations (Supplementary Fig. 52b). To validate LD as a pivotal regulator of antigen presentation, we inhibited LD formation using avasimibe (Acyl coenzyme A-cholesterol acyltransferase (ACAT) inhibitor) and lipofermata (fatty acid transport proteins (FATP) inhibitor). This achieved reduced LD accumulation and increased antigen presentation (~4.9-fold), which was much lower than that of irradiated CCR2-DC-ANVs (~16.8-fold) (Supplementary Fig. 53). These findings indicated that isolated reduction of LD accumulation was insufficient to fully restore antigen presentation. Critically, sole LD depletion failed to rescue mitochondrial fragmentation or ER stress in DCs, which suggested that light-driven restoration of mitochondrial and ER homeostasis in CCR2-DC-ANVs was essential for reconstituting antigen presentation functionality. To dissect the contributions of mitochondrial and ER functional restoration to antigen presentation, we employed targeted molecular interventions. Drp1 inhibitors (mdivi-1, si-Drp1) were used for mitochondrial fusion promotion, while ROS scavenger (glutathione (GSH)) and ER stress inhibitor (4-Phenylbutyric acid (4-PBA)) were used to relieve ER stress. Confocal imaging demonstrated that mitochondrial fusion partially attenuated ER stress and reduced LD accumulation (Supplementary Fig. 54). Although ER restoration diminished LD accumulation, it failed to rescue mitochondrial fragmentation (Supplementary Fig. 54). Quantitative analysis showed that mitochondrial repair induced higher LD reduction than ER restoration. Flow cytometry revealed a hierarchical improvement in antigen presentation. CCR2-DC-ANVs exhibited higher MHC-I-peptide complex expression (~10.4-fold) than repairing mitochondrial morphology alone (~6.0-fold) and ER stress alleviation alone (~3.4-fold). These results demonstrated that mitochondrial restoration outperformed ER repair in rescuing antigen presentation, as it concurrently alleviated ER stress. Light-driven CCR2-DC-ANVs achieved coordinated mitochondrial-ER rehabilitation, driving LD clearance and optimal antigen presentation. Together, mitochondrial fusion combined with NADPH generated by ANVs reduced intracellular ROS levels, suppressing PERK activation and eIF2α phosphorylation-induced ER stress and XBP1 activation-driven LD accumulation, thereby prolonging cell survival and enhancing antigen presentation of irradiated CCR2-DC-ANVs (Fig. 3q).

### CCR2-DC-ANVs enhance antitumour immune response

Given that CCR2-DC-ANVs restore mitochondrial function upon light irradiation, we investigated the immunological performance. We found that the levels of MHC complexes and co-stimulatory molecules (CD40, CD80 and CD86) of irradiated CCR2-DC-ANVs were significantly increased, indicating strong immune activity (Supplementary Fig. 55a). To evaluated the T cell activation ability, we incubated hypoxic BMDCs and CCR2-DC-ANVs with cells isolated from murine spleen. After light irradiation, CCR2-DC-ANVs contributed to proliferation of T cells from 31.8 ± 9.3% (BMDC group) to 84.4 ± 7.1% (Supplementary Fig. 55b). Furthermore, irradiated CCR2-DC-ANVs improved the effector functions of T cells, as revealed by the enhanced population of effective CD8^+^ T (IFN-γ^+^ and GzmB^+^), CD4^+^ T (IFN-γ^+^) cells and increased secretion of effector cytokines (IL-12p70, IFN-γ and TNF-α) (Supplementary Fig. 55c, d). Besides, the level of immunosuppressive molecules (TIM-3 and LAG-3) on CD8^+^ T cells and population of immunosuppressive Th2 (IL-4^+^CD4^+^) and regulatory T (Treg) cells reduced in CCR2-DC-ANV + L group (Supplementary Fig. 55e, f). Considering the adjuvant effect of polysaccharides on ANVs^64^, we incubated C29 (toll-like receptor (TLR) 2 inhibitor) resatorvid (TLR4 inhibitor) with CCR2-DC-ANVs. We found that CCR2-DC-ANVs exhibited higher expression of CD40, CD86 and MHC-II compared to BMDCs (Supplementary Fig. 55g). This enhancement was abolished by TLR2 inhibitor C29 but unaffected by TLR4 inhibitor resatorvid. Photoactivation further increased co-stimulatory molecule and MHC I/II expression in CCR2-DC-ANVs. Light-driven metabolic reprogramming in CCR2-DC-ANVs led to restored mitochondrial function and relieved ER stress, synergistically enhanced DC maturation. Notably, we found a significant increase in MHC I and SIINFEKL-H-2Kb expression in CCR2-DC-ANVs + L group, confirming the enhanced antigen presentation of CCR2-DC-ANVs after light exposure (Supplementary Fig. 55h, i). Western blotting confirmed TLR2 activation in CCR2-DC-ANVs + L group, comparable to Pam3CSK4 (TLR2 agonist)-treated BMDCs (Supplementary Fig. 55j). Taken together, the inherent TLR2-mediated adjuvant effect of ANVs initiates DC maturation, and photoactivation conducts metabolic reprogramming, which further enhanced DC maturation.

**Fig. 4:**
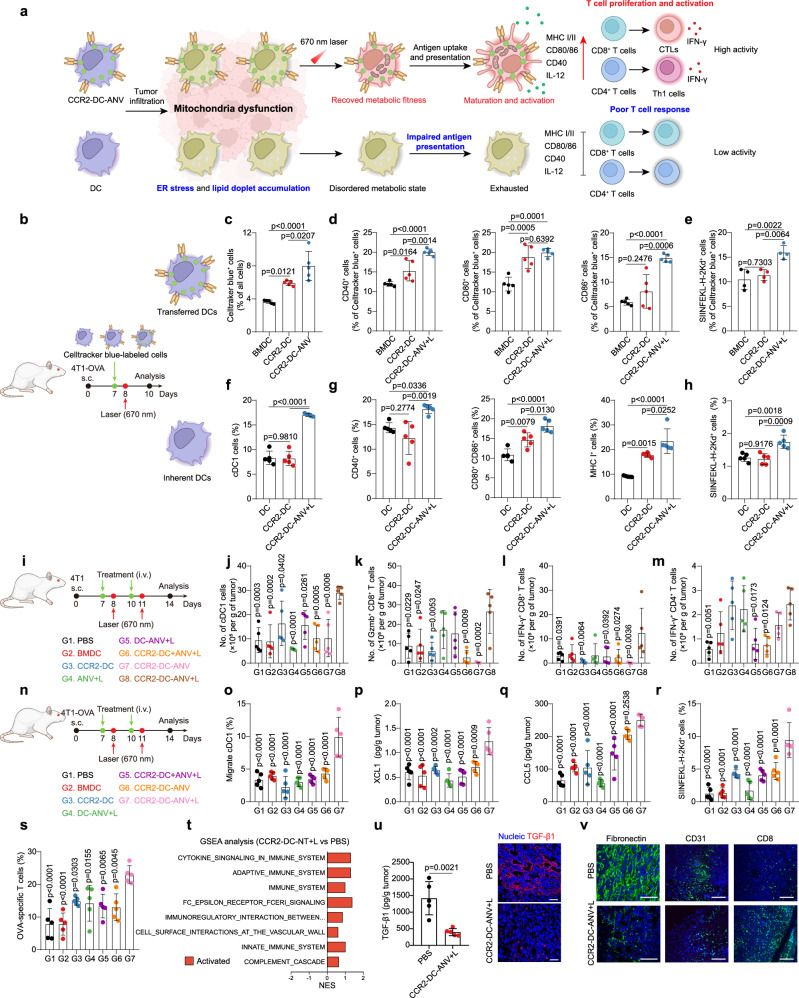
CCR2-DC-ANVs enhance anticancer immune response in vivo. **a** Scheme illustration of the immune activation of CCR2-DC-ANVs. BMDCs in TME show low activity and unable to induce sufficient T cell response. CCR-DC-ANVs perform enhanced activity after red light irradiation and significantly improve T cell proliferation and activation. **b** Procedure to study immune activation of CCR2-DC-ANVs and tumour-infiltrated DCs in vivo. BMDCs, CCR2-DCs and CCR2-DC-ANVs were labelled with celltracker blue and intravenously administrated. **c**, Quantitative analysis of celltracker blue^+^ cells in tumour (*n* = 5). Quantitative analysis of CD40, CD80 and CD86 expression (**d**) and SIINFEKL-H-2Kd^+^ cells (**e**) among celltracker blue^+^ cells (*n* = 5). **f** Quantitative analysis of cDC1s in tumour (*n* = 5). Quantitative analysis of CD40, CD80, CD86 and MHC-I expression (**g**) and SIINFEKL-H-2Kd^+^ cells (**h**) among tumour-infiltrated DCs (*n* = 5). **i** Scheme of the experimental design to study the in vivo immune response of CCR2-DC-ANVs. Quantitative number of cDC1s (**j**), GzmB^+^ CD8^+^ T cells (**k**), IFN-γ^+^ CD8^+^ T cells (**l**) and IFN-γ^+^ CD4^+^ T cells (**m**) in the tumour (*n* = 5). **n** Scheme of the experimental design to study cDC1 migration after different treatments. Quantitative analysis of mig-cDC1s (**o**), XCL1 level (**p**), CCL5 level (**q**), SIINFEKL-H-2Kd^+^ cells (**r**) and OVA-specific CD8^+^ T cells (**s**) in the tumour (*n* = 5). **t** GSEA enrichment analysis of biological processes between the PBS and CCR2-DC-ANV plus light exposure groups (*n*  =  3). **u** Quantitative analysis and representative immunofluorescence images of TGF-β1 level (*n* = 5). **v** Representative immunofluorescence images of fibronectin, CD31 and CD8 in the tumour (*n*  =  3). Scale bar, 75 μm. Data are mean ± s. d. Statistical analysis was evaluated with Student’s two-tailed unpaired *t-*test (**u**) and one-way ANOVA (**c**–**h, j**–**m**, **o**–**s**).

Encouraged by the strong capability of CCR2-DC-ANVs to boost immunity in vitro, we investigated the in vivo immunological effects in breast cancer-bearing mice (Fig. 4a). First, we studied the in vivo distribution of CCR2-DC-ANVs. We observed a strong chlorophyll (labelling ANVs) signal in the tumour area 24 h after the *i.v*. injection of CCR2-DC-ANVs, and the majority of BMDCs (labelled with DiI) in CCR2-DC-ANVs were also located in the tumour region (Supplementary Fig. 56a–c). To further evaluate the penetration capability, the vasculature was labelled with anti-CD31 antibody. The DiI and chlorophyll signals of CCR2-DC-ANVs were diffused out of the vasculature and penetrated to adjacent areas, whereas BMDCs barely infiltrate into tumours (Supplementary Fig. 56d and Supplementary Fig. 57). Mature tumour-infiltrating DCs migrate to the tumour-draining lymph node (tdLNs) in response to chemokines. According to the superior tumour tropism, CCR2-DC-ANVs exhibited significant tdLN accumulation while BMDCs showed negligible tdLN trafficking (Supplementary Fig. 58a). When we further compared the tdLN distribution of CCR2-DC-ANVs versus free ANVs using ANV fluorescence, we also observed superior accumulation of CCR2-DC-ANVs in tdLNs while ANVs exhibited negligible accumulation in tdLNs (Supplementary Fig. 58a). Immunofluorescence staining confirmed the migratory capacity of CCR2-DC-ANVs to tdLNs (Supplementary Fig. 58b, c). Furthermore, we dual-labelled CCR2-DC-ANVs with DiI (membrane) and ANV autofluorescence and observed both DiI and ANV signals simultaneously in tdLNs (Supplementary Fig. 59).

To evaluate the photosynthetic-boosted immune activation based on CCR2-DC-ANVs in vivo, we injected dye-labelled cells into 4T1-OVA tumour-bearing mice (Fig. 4b). We found an increased population of injected DCs in tumours from 3.6 ± 0.2% (BMDC group) to 8.0 ± 1.8% (CCR2-DC-ANV group) (Fig. 4c). CCR2-DC-ANV + L treatment improved the effector functions of injected DCs, as revealed by the enhanced expression of CD40, CD80, CD86 and MHC complexes (Fig. 4d). Notably, light exposure significantly increased SIINFEKL presented by CCR2-DC-ANVs (Fig. 4e). Except CCR2-DC-ANVs, endogenous DCs also showed enhanced immunological efficacy, characterized by increased population of tumour-infiltrating cDC1s, enhanced DC maturation and improved antigen cross-presentation (Fig. 4f-h and Supplementary Fig. 60).

Next, we investigated the ability of CCR2-DC-ANVs to ameliorate the immunosuppressive TME. After treatment with different suspensions for two times, the cells in tumour tissues were isolated and examined (Fig. 4i and Supplementary Fig. 61). We found an increased number of cDC1 and cDC2 and enhanced DC maturation in tumours, which resulted in activation of CD8^+^ T and CD4^+^ T cells (Fig. 4j and Supplementary Fig. 62). The significantly increased number of effective CD8^+^ T (IFN-γ^+^ and GzmB^+^) and CD4^+^ T (IFN-γ^+^) cells indicated the immune activation triggered by irradiated CCR2-DC-ANVs and inherent cDCs (Fig. 4k-m). The level of immunosuppressive molecules (PD-1, TIM-3 and LAG-3) also decreased in CD8^+^ T cells (Supplementary Fig. 63). Additionally, the level of M1-like tumour-associated macrophages (TAMs) increased while the levels of Th2, Treg and M2-like TAMs decreased in the tumour (Supplementary Fig. 64). After treated with CCR2-DC-ANV for 3 days, increased IL-12p70 which released mainly by DCs, along with high levels of IFN-γ and TNF-α were detected in the tumour tissues (Supplementary Fig. 64). Further evaluation showed that migratory cDC1s (mig-cDC1s) infiltrated into tumours after CCR2-DC-ANV + L treatment (Fig. 4n, o and Supplementary 65). Activated T cells and natural killer (NK) cells secreted XCL1 and CCL5 to guide mig-cDC1s into tumours^8,65,66^,^667^. We found an increased level of XCL1 and CCL5 in tumours after CCR2-DC-ANV + L treatment, which was associated with the tumour homing of mig-cDC1s (Fig. 4p, q and Supplementary Fig. 66). We also observed elicited antigen presentation in tumour-infiltrating cDCs and the expansion of OVA-specific CD8^+^ T cells in CCR2-DC-ANV + L group (Fig. 4r, s). To further elucidate the mechanisms underlying the immunological efficacy of CCR2-DC-ANVs, we performed intratumoural RNA sequencing. CCR2-DC-ANV + L group occupied a distinct area from control group and showed higher expression of *CD83* (Supplementary Fig. 67). GO analysis showed gene expression enrichment of innate immunity signals (c-type lectin, NOD-like and toll-like receptor signal pathways), T cell activation and cytokine-cytokine receptor interaction (Supplementary Fig. 68). Moreover, GSEA revealed the important role of major activation pathways including cytokine signalling as well as adaptive immune systems in CCR2-DC-ANV + L group (Fig. 4t). Furthermore, CCR2-DC-ANV + L treatment significantly downregulated intratumoural TGF-β1 (Fig. 4u), likely mediated by IFN-γ upregulation in TME^67^. TGF-β1 drives tumour fibrosis via the TGF-β/Smad signalling pathway, activating cancer-associated fibroblasts (CAFs) to overproduce fibronectin and impair immune cell infiltration^34,35^. Immunofluorescence staining showed intratumoural fibronectin disruption and large numbers of CD8^+^ T cell infiltration after CCR2-DC-ANV + L treatment (Fig. 4v). In addition, we also observed an anti-angiogenic effect and perivascular accumulation of macrophages following CCR2-DC-ANV + L treatment (Fig. 4v and Supplementary Fig. 69). It might involve a direct antiangiogenic effect of IFN-γ and an indirect mechanism of M1 macrophages^68,69^. Collectively, these results suggested that the plant vesicle-DC chimera increased recruitment of mig-cDC1s, activated multi-pathway anti-tumour immunity and reversed immunosuppressive TME.

### CCR2-DC-ANVs enhance therapeutic efficiency against multiple tumours

**Fig. 5:**
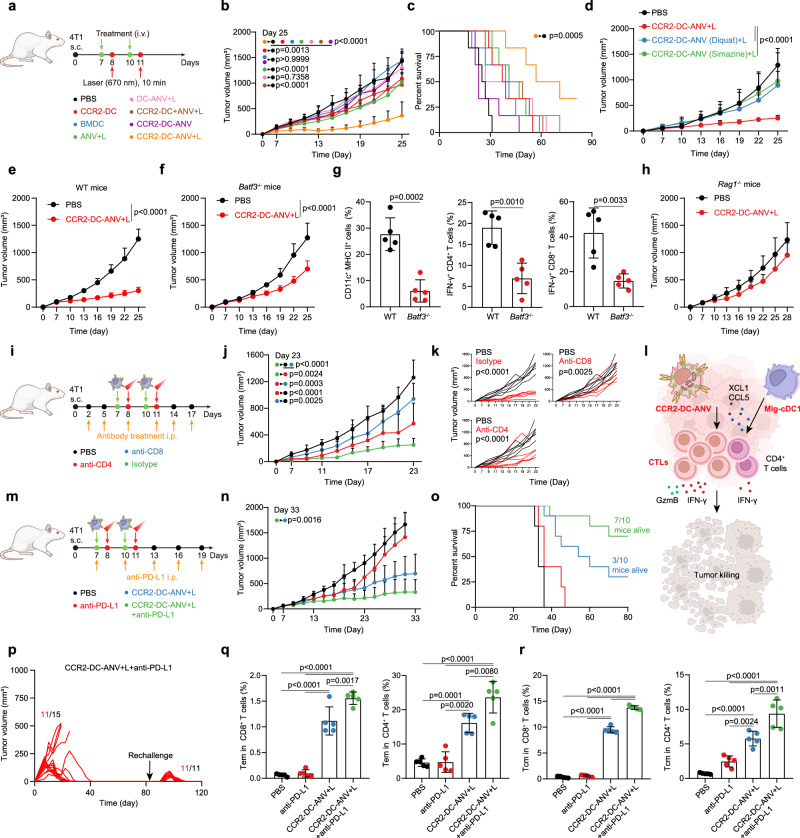
Antitumour efficacy of CCR2-DC-ANVs. **a** Scheme of the experimental design for treating primary 4T1 tumour using CCR2-DC-ANVs. **b** 4T1 tumour growth curves after treatment (*n* = 6 for PBS group and *n* = 5 for other groups). **c** Survival curves of mice after treatment (*n* = 6). **d** 4T1 tumour growth curves after macrophage depletion (*n* = 5). B16-OVA tumour growth curves in C57BL/6 mice (**e**) and *Batf3*^–/–^ (**f**) mice after CCR2-DC-ANV treatment (*n* = 6 for PBS and *n* = 8 for CCR2-DC-ANV + L). **g** Quantitative analysis of cDC1s, IFN-γ^+^ CD8^+^ T cells and IFN-γ^+^ CD4^+^ T cells in the tumour of C57BL/6 mice and *Batf3*^–/–^ mice after CCR2-DC-ANV treatment (*n* = 5). **h**, B16-OVA tumour growth curves in *Rag1*^–/–^ mice after CCR2-DC-ANV treatment (*n* = 5). **i** Scheme of the experimental design for CD8^+^ and CD4^+^ T cell depletion in primary 4T1 tumour. **j**, **k** 4T1 tumour growth curves after treatment (*n* = 6). **l** Scheme illustration of potential mechanisms of tumour response to CCR2-DC-ANVs in vivo. Adoptively transferred CCR2-DC-ANVs activate effector T cells, which secrete XCL1 and CCL5 to recruit endogenous migratory cDC1s. These recruited cDC1s synergistically amplify the activation of tumour-specific T cells with CCR2-DC-ANVs, thereby establishing potent anti-tumour immunity. **m** Scheme of the experimental design for treating primary 4T1 tumour using CCR2-DC-ANVs with anti-PD-L1 antibody. **n** 4T1 tumour growth curves after treatment (*n* = 5 for PBS and anti-PD-L1 group and *n* = 10 for other groups). **o** Survival curves of mice after treatment (*n* = 5 for PBS and anti-PD-L1 group and *n* = 10 for other groups). **p** 4T1 tumour growth curves after coordinated treatment (*n* = 15). Mice with a complete response (*n*  =  11) were re-challenged with 4T1 cells at day 82. Quantitative analysis of Tem (CD44^+^ CD62L^-^) (**q**) and Tcm (CD44^+^ CD62L^+^) (**r**) in spleen 30 days after treatment (*n* = 5). Data are mean ± s. d. Statistical analysis was evaluated with Student’s two-tailed unpaired *t-*test (**g**), one-way ANOVA (**q**, **r**), two-way ANOVA (**b**, **d**–**f**, **h**, **j**, **k**, **n**) and log-rank (Mantel-Cox) test (**c**, **o**).

Inspired by the remarkable capability to boost antitumour immunity, we next investigated the antitumour efficiency of CCR2-DC-ANVs in 4T1 tumour-bearing mice (Fig. 5a). All groups maintained stable body weights during the treatments (Supplementary Fig. 70). The BMDC group and the CCR2-DC-ANV group (without light exposure) could barely inhibit the tumour growth when compared to the PBS group (Fig. 5b). In contrast, the mice of the CCR2-DC-ANV + L group showed the slowest tumour growth rate over a period of 25 days. Accordingly, 33.3% of mice survived over 81 days when treated with CCR2-DC-ANV + L (Fig. 5c). Lung metastasis inhibition in the CCR2-DC-ANV + L group indicated that it inhibited spontaneous lung metastasis of breast cancer (Supplementary Fig. 71). To assess the impact of 670 nm light irradiation on tumour-infiltrating T cells, tumours were exposed to laser (600 mW/cm²) for different durations. Crucially, even 30-min irradiation failed to significantly alter the infiltration and function of CD8⁺ T cells in tumours or tumour-draining lymph nodes versus controls (Supplementary Fig. 72). Single light exposure alone did not inhibit tumour growth, contrasting sharply with CCR2-DC-ANV efficacy. Besides, inhibition of PSI or PSII abolished the anti-tumour efficacy of CCR2-DC-ANVs (Supplementary Fig. 73).

To investigate endogenous immune modulation, TAMs were selectively depleted using anti-CSF1R antibody (Supplementary Fig. 74). This intervention potentiated CCR2-DC-ANV efficacy, mechanistically linked to reduced immunosuppressive TME formation and restored T cell infiltration (Fig. 5d). Given the robust recruitment of mig-cDC1s and effector T cells following CCR2-DC-ANV + L treatment, we interrogated whether endogenous cDC1s and T cells are essential for mediating CCR2-DC-ANV-induced tumour suppression. Absence of endogenous cDC1s in *Batf3*^*−/−*^ recipient mice reduced the therapeutic activity of CCR2-DC-ANV and failed to elicit robust T-cell activation in tumours (Fig. 5e–g and Supplementary Figs. 75, 76), suggesting that CCR2-DC-ANVs engaged endogenous cDC1s to activate antitumour immunity. Besides, CCR2-DC-ANVs failed to suppress tumour growth in *Rag1*^−/−^ mice (Fig. 5h), suggesting that endogenous T cells were essential for CCR2-DC-ANV efficacy and CCR2-DC-ANV itself could not directly inhibit tumour growth. Furthermore, we conducted cell depletion studies in 4T1 tumour-bearing mice to gain further insight into the involvement of T cells (Fig. 5i). It revealed that tumour growth was significantly faster following anti-CD8 antibody treatment than following anti-CD4 treatment, indicating that antitumour efficacy is primarily mediated by CD8⁺ T cells (Fig. 5j, k). Taken together, these data established that the antitumour efficacy of CCR2-DC-ANVs required synergistic contributions from both adoptively transferred DCs and endogenous cDC1s, which cooperatively amplify CD8⁺ T cell responses through optimized antigen presentation and T cell priming (Fig. 5l).

To further boost the therapeutic efficacy, we combined CCR2-DC-ANV + L therapy with anti-PD-L1 antibody therapy and demonstrated that anti-PD-L1 could further enhance CCR2-DC-ANV efficacy, leading to sustained inhibition of tumour growth and prolonged survival rate from 30% to 70% (Fig. 5m–o). Tumour rechallenge experiments revealed durable protective immunity in 100% of cured mice (11/11), which rejected secondary tumour implants, demonstrating CCR2-DC-ANV-induced sterilizing immune memory (Fig. 5p). Further evaluation revealed that the combination therapy elicited potent long-term immune memory, characterized by significant expansion of splenic effector memory T cells (T_em_) and central memory T cells (T_cm_) in spleen (Fig. 5q, r and Supplementary Fig. 77).

To investigate the antitumour efficiency in disseminated tumour models, Balb/c mice were inoculated with CT26-Luc cells by *i.p*. injection (Supplementary Fig. 78a). We observed a strong DiR (labelling DCs) signal in the tumour area 24 h after the *i.v*. injection of CCR2-DC-ANVs, and it was highly overlapped with the biofluorescence of tumour tissues (Supplementary Fig. 78b, c). In contrast, BMDCs showed poor tumour-targeted accumulation. Immunofluorescence staining revealed that CCR2-DC-ANVs migrated to tumour foci through blood vessels (Supplementary Fig. 78d). These results suggest that CCR2-DC-ANVs not only infiltrate subcutaneous tumours, but also precisely target disseminated tumour lesions. The fluorescence signal of metastatic lesions was lower in mice receiving CCR2-DC-ANV + L than those receiving other suspensions (Supplementary Fig. 78e). Moreover, 60% of mice receiving CCR2-DC-ANV + L treatment survived over 60 days, while no mice in other groups survived longer than 50 days (Supplementary Fig. 78f). Accordingly, CCR2-DC-ANVs increased the population of effective CD8^+^ T (IFN-γ^+^ and GzmB^+^) and CD4^+^ T (IFN-γ^+^), and reprogrammed TME of abdominal dissemination colorectal cancer (Supplementary Fig. 78g–p and Supplementary Figs. 79 and 80).

### Immunogenicity and biosafety of CCR2-DC-ANVs

Due to the intrinsic immunogenicity of plant-derived nanovesicles in mammals, we evaluated the immunogenicity of ANVs and CCR2-DC-ANVs following initial administration in mice. Both types of particles induced transient splenomegaly within 24–48 h post intravenous injection (Supplementary Fig. 81a). Comprehensive assessment of humoral and cellular immune responses revealed significant expansion of activated CD4⁺ and CD8⁺ T cells (IFN-γ⁺) in the spleen in both treatment groups, with higher T cell activation observed in the CCR2-DC-ANV group (Supplementary Fig. 81b, c). Upon in vitro re-stimulation with ANVs, splenic T cells isolated 14 days post-injection showed selective reactivation of CD8⁺ T cells, but not CD4⁺ T cells (Supplementary Fig. 81d, e). These findings demonstrated that ANV-based formulations elicited transient cellular immunity, dominated by effector CD8⁺ T cell responses. The enhanced immunogenicity of CCR2-DC-ANVs may stem from DC-derived immunostimulatory molecules. Notably, immunopeptidomic profiling confirmed that CCR2-DC-ANVs present algal-derived peptides via both MHC class I and II pathways (Supplementary Data 1, 2), without compromising tumour antigen presentation or impairing tumour-specific T cell activation.

To evaluate anti-ANV humoral immunity, we monitored serum antibody dynamics. Both ANVs and CCR2-DC-ANVs triggered transient IgG1 production, returning to baseline by day 4. CCR2-DC-ANVs induced stronger IgG3 responses than ANVs throughout the study (Supplementary Fig. 82a–f). Given the algal origin of ANVs, we assessed IgG-mediated allergen binding by incubating post-immunization sera with algae. Flow cytometry revealed peak IgG binding (3.9 ± 1.7%) at 24 h after ANV administration, which correlated strongly with serum IgG1 levels (Supplementary Fig. 82g). No significant binding was observed with CCR2-DC-ANV sera. Binding returned to baseline by 48 h, indicating mild and transient immunogenicity.

We next assessed the in vivo biosafety of ANVs and CCR2-DC-ANVs following a single intravenous dose in healthy mice. Systemic inflammation was monitored by measuring serum IL-6, a key cytokine indicative of cytokine release syndrome (CRS). Neither formulation significantly elevated IL-6 levels, indicating absence of systemic CRS (Supplementary Fig. 82h). Histopathological evaluation of liver and lung tissues via hematoxylin & eosin (H&E) staining further confirmed the lack of pathological inflammation or tissue injury (Supplementary Fig. 83). In addition, CCR2-DC-ANV + L therapy showed a favourable safety profile without causing damage to major organs (Supplementary Fig. 84). These results demonstrated that while ANVs triggered only mild and transient immune activation, CCR2-DC-ANVs elicited moderately stronger immunogenicity-likely due to immunostimulatory molecules derived from the adoptive DCs. Crucially, both formulations avoided inducing cytokine storm or organ toxicity. Although anti-ANV immune responses were observed, ANVs remained encapsulated within the cytoplasm of CCR2-DC-ANVs rather than being exposed on the cell surface via fusion or membrane insertion. ANV-derived peptides were processed and presented strictly through MHC molecules. Thus, even if host cytotoxic T cells recognized ANV antigens, they would not target CCR2-DC-ANVs in the absence of membrane-displayed epitopes. Together, these findings indicate that ANV-based formulations cause minimal disruption to host physiological homeostasis.

### CCR2-DC-ANVs perform superior therapeutic efficiency than DC vaccines

**Fig. 6:**
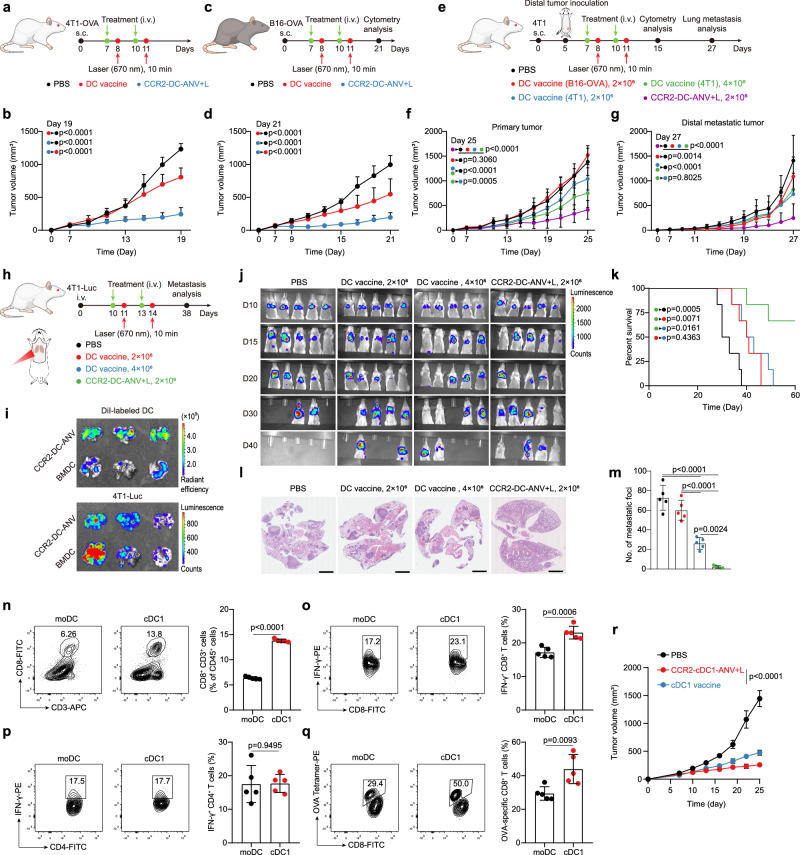
Antitumour efficacy of CCR2-DC-ANVs compare to lysate-pulsed autologous DC vaccines. **a** Scheme of the experimental design for treating primary 4T1-OVA tumour using CCR2-DC-ANVs and DC vaccines. **b** 4T1-OVA tumour growth curves after treatment (*n* = 6 for PBS group and *n* = 5 for other groups). **c** Scheme of the experimental design for treating primary B16-OVA tumour using CCR2-DC-ANVs and DC vaccines. **d** 4T1 tumour growth curves after treatment (*n* = 7). **e** Scheme of the experimental design and light exposure method for treating distal metastatic tumour using CCR2-DC-ANVs and DC vaccines. **f**, **g** Tumour growth curves of primary tumour and distal metastatic tumour after treatment (*n* = 7). **h** Scheme of the experimental design and light exposure method for treating lung metastatic 4T1-Luc tumour using CCR2-DC-ANVs and DC vaccines. **i** Representative images of fluorescence and bioluminescence in lung after treatment. **j** In vivo bioluminescence images of lung metastatic 4T1-Luc tumour-bearing mice. **k** Survival curves of 4T1-Luc tumour-bearing mice after treatment (*n* = 6). **l** Representative H&E staining images of lung metastasis. Scale bar, 2.5 mm. **m** Total number of lung surface metastases (*n* = 5). Representative flow cytometry plots and quantification of CD8^+^ T cells (**n**), IFN-γ^+^ CD8^+^ T cells (**o**), IFN-γ^+^ CD4^+^ T cells (**p**) and OVA-specific CD8^+^ T cells (**q**) induced by borrow-derived DCs and cDC1s (*n* = 5). **r** 4T1-OVA tumour growth curves after treating with cDC1 vaccine and CCR2-cDC1-ANVs (*n* = 6). Data are mean ± s. d. Statistical analysis was evaluated with Student’s two-tailed unpaired *t-*test (**n**–**q**), one-way ANOVA (**m**), two-way ANOVA (**b**, **d**, **f**, **g**, **r**) and log-rank (Mantel–Cox) test (**k**).

Considering that tumour lysate-pulsed autologous DC vaccines have been used in clinical trials^18,70^, we compared CCR2-DC-ANVs with DC vaccines to explore the clinical translation potential. The successful construction of DC vaccines was validated by flow cytometry (Supplementary Fig. 85). In both breast cancer and melanoma mouse models, CCR2-DC-ANVs showed better therapeutic effects than DC vaccines (Fig. 6a–d). We found that CCR2-DC-ANV + L treatment showed enhanced DC maturation in tdLNs and an increased number of effector T cells and OVA-specific CD8^+^ T cells in tumours than that of DC vaccine (Supplementary Fig. 86a–d). These data suggest that CCR2-DC-ANVs generate stronger antitumour immune activation than DC vaccines, resulting in enhanced therapeutic efficacy. Subsequently, we compared therapeutic efficiency of CCR2-DC-ANVs and DC vaccines with autologous and allogeneic tumour antigens in primary breast tumours and distal tumours (Fig. 6e). Allografts from 4T1 and B16-OVA were used as autologous and allogeneic antigen sources for the production of DC vaccines. We also evaluated the efficacy of CCR2-DC-ANVs and double-dosed DC vaccines (Fig. 6f, g). DC vaccine (B16-OVA) could barely inhibit 4T1 breast cancer. The double-dosed DC vaccines slowed down primary tumour growth compared to the single-dosed DC vaccines, but did not show efficient suppression of distal tumours. Notably, both primary and distal metastatic tumours exhibited the slowest growth rates compared to the other groups when treatment with CCR2-DC-ANVs + L. Furthermore, we evaluated the infiltration of immune cells in the primary and distal tumours after treatment. Compared with the other groups, the intratumoural cDC1 population was significantly increased after CCR2-DC-ANV + L treatment, resulting in enhanced infiltration of endogenous effective CD8^+^ T (IFN-γ^+^ and GzmB^+^) and CD4^+^ T (IFN-γ^+^) in both primary and distal metastatic tumours (Supplementary Fig. 86e, f). Besides, we found increased numbers of T_em_ and T_cm_ cells in the spleen, indicating that CCR2-DC-ANVs initiated a strong immune memory response (Supplementary Fig. 86g–i). More importantly, CCR2-DC-ANVs performed more effectively in inhibiting spontaneous metastasis, and few visible metastatic nodules were observed in the lungs (Supplementary Fig. 86j, k).

To evaluate the efficacy of CCR2-DC-ANVs and DC vaccines in lung metastatic breast cancer model, Balb/c mice were inoculated with 4T1-Luc cells by *i.v*. injection (Fig. 6h). Strong DiI (labelling DCs) signals were observed in the tumour area 24 h after the *i.v*. injection of CCR2-DC-ANVs, and it was highly overlapped with the biofluorescence of lung metastatic lesion (Fig. 6i and Supplementary Fig. 87). In contrast, BMDCs showed lower lung accumulation and poor tumour-targeting ability. Immunofluorescence staining revealed that CCR2-DC-ANVs migrated to lung through blood vessels and mainly localized in the tumour, rather than normal lung tissue (Supplementary Fig. 88). Mice receiving CCR2-DC-ANV + L therapy showed much weaker bioluminescence of metastatic tumours compared to those receiving other treatments (Fig. 6j). 60% of mice receiving CCR2-DC-ANV treatment survived over 60 days, while mice receiving DC vaccines had a median survival time of 40.5 and 41.5 days, respectively (Fig. 6k). Besides, CCR2-DC-ANV therapy led to significant metastasis inhibition with no visible metastatic tumour nodules in the lungs, while some metastatic foci were not cleared with DC vaccine therapy (Fig. 6l, m). These results indicate that CCR2-DC-ANVs, which are independent of antigen pulsation, have stronger anti-metastasis effects compared to DC vaccines in treating systemic metastatic tumours.

cDC1s are the core executors of anti-tumour immunity. Given recent evidence that cDC1-based vaccines exhibit enhanced cross-presentation capacity and show excellent performance in pancreatic cancer^71^, we generated bone marrow-derived cDC1s. Compared to GM-CSF/IL-4-induced moDCs, cDC1s showed higher CD103 and Clec9A expression (Supplementary Fig. 89), the latter of which is outstanding in presenting dead cell-associated antigens and enhancing T cell responses^72^. We found that cDC1s enhanced CD8^+^ T cell activation and induced higher proportion of antigen-specific CD8^+^ T cells than moDCs (Fig. 6n–q). Furthermore, we explored the therapeutic efficacy of the cDC1s-based plant vesicle-DC chimera (CCR2-cDC1-ANVs) and found a superior tumour control than cDC1-based vaccines (Fig. 6r). Together, cDC1s outperformed GM-CSF/IL-4-induced moDCs via enhanced T cell priming capability. The therapeutic platform of plant nanovesicle-DC chimeras has now been validated in cDC1s, confirming that CCR2-DC-ANVs represent a promising clinically translatable platform for DC-based immunotherapy.

### Manufacturing CCR2-DC-ANVs with human peripheral blood mononuclear cells

**Fig. 7:**
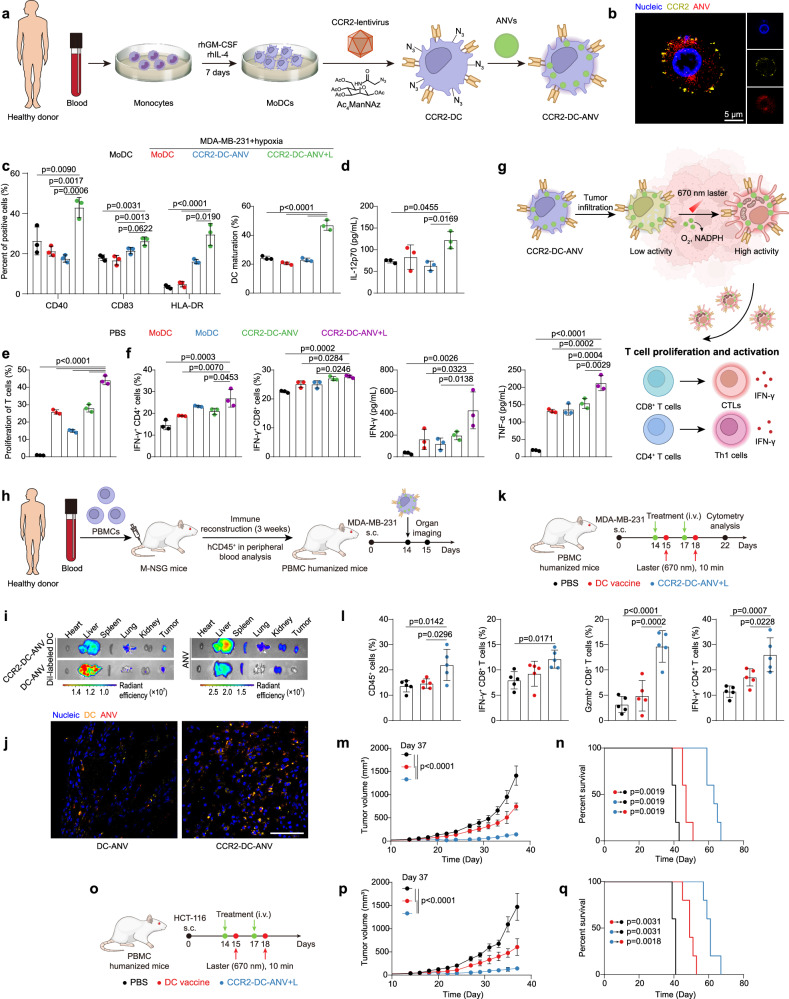
Fabrication and characterization of CCR2-DC-ANVs derived from human moDCs and their antitumour efficacy against humanized CDX models. **a** Scheme illustration of the construction of CCR2-DC-ANVs using moDCs. **b** Representative immunofluorescence images of CCR2-DC-ANVs (*n*  =  3). Scar bar, 5 μm. **c** Quantitative analysis of CD40, CD83 and HLA-DR expression and matured cells (CD80^+^ CD86^+^) among moDCs and CCR2-DC-ANVs (*n* = 3). **d** IL-12p70 level in cell culture medium measured by ELISA (*n* = 3). **e** Quantitative analysis of the proliferation rate of T cells isolated from peripheral blood (*n* = 3). **f** Quantitative analysis of IFN-γ^+^ CD8^+^ T cells, IFN-γ^+^ CD4^+^ T cells and levels of IFN-γ and TNF-α after CCR2-DC-ANV incubation (*n* = 3). **g** Scheme illustration of the immune activation of CCR2-DC-ANVs. CCR-DC-ANVs in TME perform enhanced activity after red light irradiation and significantly improve T cell proliferation and activation. **h** Scheme of the preparation of PBMC-humanized CDX models. **i** Representative images of fluorescence and bioluminescence in different organs after treatment (*n*  =  3). **j** Representative immunofluorescence images of BMDCs and CCR2-DC-ANVs in tumour (*n*  =  3). Scale bar, 75 μm. **k** Scheme of the experimental design of moDC-derived CCR2-DC-ANV therapy against a PBMC-humanized MDA-MB-231 tumour-bearing model. **l** Quantitative analysis of CD45^+^ cells, IFN-γ^+^ CD8^+^ T cells, GzmB^+^ CD8^+^ T cells and IFN-γ^+^ CD4^+^ T cells in tumour (*n* = 5). **m** Tumour growth curves after treatment (*n* = 5). **n** Survival curves of mice after treatment (*n* = 5). **o** Scheme of the experimental design of moDC-derived CCR2-DC-ANV therapy against a PBMC-humanized HCT-116 tumour-bearing model. **p** P Tumour growth curves after treatment (*n* = 5). **q** Survival curves of mice after treatment (*n* = 5). Data are mean ± s. d. Statistical analysis was evaluated with one-way ANOVA (**c**–**f**, **l**), two-way ANOVA (**m**, **p**) and log-rank (Mantel–Cox) test (**n**, **q**). CDX, cell derived xenograft.

Most preclinical and clinical experimentations with antigen-loaded DCs used moDCs^73^. Therefore, we tested whether human moDCs enable CCR2-DC-ANV manufacturing. Human moDCs were differentiated from peripheral blood monocytes, successively engineered via lentiviral transduction to express CCR2 (Supplementary Fig. 90), and loaded with algae-derived nanovesicles (ANVs) (Fig. 7a). This process yielded CCR2-DC-ANVs with high efficiency, as 91.1% of moDCs successfully incorporated ANVs and expressed CCR2 (Supplementary Fig. 91), confirmed by fluorescent staining (Fig. 7b). We then evaluated the activity of CCR2-DC-ANVs when incubated with tumour cells in hypoxia. After 670 nm laser irradiation, the levels of CD83, co-stimulatory molecules (CD40, CD80 and CD86) and human leukocyte antigen (HLA-DR) of CCR2-DC-ANVs were significantly increased, indicating strong immune activity (Fig. 7c). Accordingly, the IL-12p70 secretion also increased in the CCR2-DC-ANV + L group (Fig. 7d). To evaluated the ability to activate T cells, we incubated tumour-treated CCR2-DC-ANVs with T cells isolated from human peripheral blood. After light exposure, CCR2-DC-ANVs enhanced proliferation of T cells from 25.8 ± 1.2% (moDC group) to 44.1 ± 2.5% (Fig. 7e and Supplementary Fig. 92). Furthermore, irradiated CCR2-DC-ANVs improved the effector functions of T cells, as revealed by the enhanced population of effective CD8^+^ T (IFN-γ^+^), CD4^+^ T (IFN-γ^+^) cells and increased secretion of effector cytokines (IFN-γ and TNF-α) (Fig. 7f). These results suggest that the cross-species engineering strategy is applicable to moDCs, which repairs immune dysfunction of moDCs in TME and promote T cell proliferation and activation (Fig. 7g).

### Immunotherapy by CCR2-DC-ANVs in humanized CDX model

To further explore the potential of CCR2-DC-ANV therapy in clinically relevant animal models, we constructed a humanized cell line-derived xenograft (CDX) model in humanized NOD-Prkdc^scid^Il2rg^em1^/Smoc (NSG) mice and administrated moDC-derived CCR2-DC-ANVs (Fig. 7h). After treatment with CCR2-DC-ANVs, we observed accumulation of ANVs (labelled with chlorophyll) and moDCs (labelled with DiI) in the tumour area (Fig. 7i and Supplementary Fig. 93), where a widespread distribution within the tumour was detected as compared to DC-ANV therapy (Fig. 7j). We next investigated the in vivo immunological effects of CCR2-DC-ANVs in the MDA-MB-231 breast tumour tissues (Fig. 7k and Supplementary Fig. 94). Compared to DC vaccines, we found higher levels of CD45^+^ immune cell infiltration in tumour tissues and increased population of effective CD8^+^ T (IFN-γ^+^ and GzmB^+^), CD4^+^ T (IFN-γ^+^) cells after CCR-DC-ANV + L treatment (Supplementary Fig. 95). We also observed increased cDC1, NK (IFN-γ and GzmB) and M1-like TAM populations and decreased Th2, Treg and M2-like TAM populations after CCR2-DC-ANV therapy (Supplementary Fig. 95). As a result, CCR2-DC-ANV + L therapy strongly inhibited tumour growth over 35 days and effectively prolonged the survival of tumour-bearing mice, which achieved a median survival time of 63 days (Fig. 7m, n). Furthermore, we investigated the therapeutic efficacy in a subcutaneous established HCT116 tumour model in humanized NSG mice (Fig. 7o). Similarly, CCR2-DC-ANV + L therapy demonstrated strong therapeutic effects against HCT116 tumours and prolonged the survival of tumour-bearing mice (Fig. 7p, q). These results indicate that human moDCs are suitable for manufacturing CCR2-DC-ANVs to inhibit the growth of multiple cancers in clinically relevant humanized mouse models.

## Discussion

In this study, we developed a plant vesicle-DC chimera by incorporating algae-derived vesicles as photoactivatable metabolic modulators to reverse mitochondrial dysfunction of DCs within immunosuppressive TME. Insufficient DC infiltration and activation often lead to inadequate T cell activation^29^. By leveraging CCL2-mediated recruitment, CCR2-DCs exhibit enhanced tumour homing in both subcutaneous and metastatic models. Although STAT3 inhibitors and similar agents are under clinical investigation to alleviate DC suppression^74^, persistent mitochondrial dysfunction drives ER stress and LD accumulation. ANVs, stably integrated within the plant vesicle-DC chimera, generate oxygen and NADPH upon 670 nm light exposure. These metabolites concurrently resolve hypoxic/oxidative stress and inhibit HIF-1α-dependent Drp1^Ser616^ phosphorylation, preventing pathological mitochondrial fission. Restoring mitochondrial integrity and redox homeostasis reduces ER stress and lipotoxic LD, thereby enhancing CCR2-DC-ANV survival in hypoxic TME. Mitochondrial-ER restoration initiates metabolic reprogramming in CCR2-DC-ANVs, restoring antigen presentation potency and T cell priming efficacy. A 10-min illumination is both sufficient and optimal for sustained and long-lasting metabolic repair. Unlike free ANVs, which poorly infiltrate tumours and show limited uptake by TADCs, the engineered chimera achieves ~90% ANV loading efficiency, explaining its superior antitumour efficacy over physically mixed CCR2-DC+ANV controls. Different from the mitochondrial transplantation technique^37^, our platform represents a cross-species strategy that uses photosynthesis to reprogram metabolism and rescue mitochondrial dysfunction in immune cells. Furthermore, it bypasses ex vivo antigen loading by enabling DCs to natively capture, process, and present tumour-associated antigens via endogenous MHC pathways within the TME, streamlining in situ immunity. Compared to conventional DC vaccines, CCR2-DC-ANVs offer simplified production and potent antitumour immunity against both primary and metastatic tumours.

The clinical translation of the plant vesicle-DC chimera is highly feasible. The validated efficacy and safety profiles of approved DC vaccines provide a robust clinical foundation for this cellular approach. This strategy employs lentiviral transduction, a routine method in CAR-T manufacturing, seamlessly integrated into DC differentiation, ensuring robust manufacturability. It utilizes vesicles derived from *Chlorella pyrenoidosa*, a scalable and safe source backed by its history as an edible supplement. Preclinical data have confirmed the safety and specific tumour-homing capability of the chimera. A key translational challenge, optimizing light delivery for deep-seated tumours, is addressable via emerging interventional strategies like fiber-optic devices^75,76^, which are particularly viable given the predominant residence of DCs at the tumour periphery (0–250 μm)^77,78^. Future efforts will focus on refining irradiation parameters and expanding the chimera approach to other DC subsets (cDC1 and cDC2) and therapeutic cells such as T and NK cells, underscoring its broad potential as a universal strategy to empower cell therapies against immunosuppressive TME. Overall, this study provides a promising plant vesicle-DC chimera for next-generation cell therapies.

## Methods

### Materials

Dibenzocyclooctyne-polyethylene glycol 2000-N-hydroxylsuccinimide (DBCO-PEG_2000_-NHS ester) was purchased from RuixiBiotech Co., Ltd. (Xi’an, China). N-azidoacetylmannosamine (Ac_4_ManNAz), Pam3CSK4, Rapamycin, Mdivi-1, Dynasore, 4-PBA, L-Glutathione reduced (GSH), Simazine, Diquat dibromide, TMRM Perchlorate, Avasimibe, Lipofermata and BODIPY 493/503 were purchased from MedChemExpress (New Jersey, USA). DAPI, DiR and DiI were purchased from Meilun Biotechnology (Dalian, China). [Ru(dpp3)] Cl2, ER-Tracker Green and MitoTracker Green were purchased from Maokang Biotechnology Co., Ltd. (Shanghai, China). BCA Protein Assay Kit, MitoSO™ Red and Hoechst 33342 were purchased from Beyotime Biotechnology Co., Ltd. (Shanghai, China). Cell culture-grade dimethyl sulfoxide (DMSO), RIPA lysis buffer, DNase I, Collagenase IV, Rhodamine 123, Fluo-4/AM, Rhod-2/AM, Annexin V-FITC Apoptosis Detection Kit and Cell Counting Kit-8 were purchased from Yeasen Biotech Co., Ltd. (Shanghai, China). ELISA kits for mouse and human CCL2/MCP-1 (cat. no. EMC113.48), IFN-γ (cat. no. EMC101g.48 and EHC102g.48), IL-12p70 (cat. no. EMC006.48 and EHC010.48), IL-12p40 (cat. no. EMC109.48), CCL4 (cat. no. EMC137b.48), CCL5 (cat. no. EMC106.96), IL-6 (cat. no. EMC004.48), IgG (cat. no. EMC116.48), IgG1 (cat. no. EMC130.48), IgG2a (cat. no. EMC131.48), IgG2b (cat. no. EMC132.48), IgG3 (cat. no. EMC133.48), IgM (cat. no. EMC129.48) and TNF-α (cat. no. EMC102a.48 and EHC103a.48) were bought from Neobioscience Technology Co., Ltd. (Shenzhen, China). ELISA kits for mouse XCL1 (cat. no. USEA795Mu) were purchased from Uscn Life Science Inc. (Wuhan, China). ELISA kits for mouse IgG4 (cat. no. KTE4014) were purchased from Abbkine Scientific Co., Ltd (Wuhan, China). Recombinant murine IL-4 and GM-CSF and human IL-4 and GM-CSF were purchased from PeproTech (USA). CellTrace™ Blue (cat. no. C34568) was purchased from Invitrogen (California, USA). ATP content (cat. no. BC0300) and NADP (H) (cat. no. BC1100) content detection kits were purchased from Solarbio Technology Co., Ltd. (Beijing, China). Coldronate Liposomes (cat. no. C-005) were purchased from Target Technology Co. Ltd. (Beijing, China). All other reagents were used as received. Antibodies used in the experiments are provided in Supplementary Table 1.

### Cell lines and animals

DC2.4, HCT116 and B16-OVA cell lines were purchased from Jinyuan Biotechnology Co., Ltd. (Shanghai, China). 4T1-OVA cell line was purchased from Fuheng Biotechnology Co., Ltd. (Shanghai, China). MDA-MB-231, CT26, CT26-Luc, B16F10, 4T1, 4T1-Luc and 4T1-GFP cell lines were purchased from the Cell Bank of Shanghai, Chinese Academy of Sciences. *Ccl2*-KO 4T1 cell line was purchased from Hanbio Biotechnology Co., Ltd. (Shanghai, China). The cells were tested mycoplasma-negative before use. All cell lines were cultured in RPMI 1640 medium containing 10% fetal bovine serum (FBS, Gibco, USA), 2.5 g/L of glucose, 0.11 g/L of sodium pyruvate and 1% penicillin/streptomycin at 37 °C in a humidified atmosphere of 95% air and 5% CO_2_. Cells were stored at −80 °C in CELLSAVING freezing medium (NCM Biotech, Suzhou, China). To construct the hypoxic environment, cells were put into the hypoxia cell incubator chamber (StemCell, Canada). In brief, 0.1% O_2_ gas, 5% CO_2_, and N_2_ (as a balance) were purged into the chamber at 20 L/min for 5 min, then the inlet and outlet ports were sealed. They were put into the incubator at 37 °C.

Female Balb/c mice (20–22 g) and female C57BL/6 (CD45.2) mice (20–22 g) were purchased from Shanghai Experimental Animal Center. Female C57BL/6 (CD45.1) mice, female M-NSG (NOD-PrkdcscidIl2rgem1/Smoc) mice, female *Ccl2−/−* mice, female *Batf3−/−* mice and female *Rag3−/−* mice were purchased from Shanghai Model Organisms Center, Inc. Mice were housed in groups of 5 mice per cage, maintained at a temperature of ~25 °C in a humidity-controlled environment with a 12 h light/dark cycle. All animal procedures were performed under the guidelines approved by the Institutional Animal Care and Use Committee of the Shanghai Institute of Materia Medica, Chinese Academy of Sciences (2024-06-LYP-46 and 2025-05-LYP-47).

### Tissue microarrays

Tissue microarrays (TMAs) were purchased from Yanteng Biotechnology Co., Ltd. (Shanghai, China) and Wknowbio Technology Co., Ltd. (Shanghai, China) with approval number SHYJS-CP-240103. Breast carcinoma TMAs (AF-BrcSur2201: array point diameter, 1.5 mm, containing 80 breast carcinomas; BRC1603: array point diameter, 1.5 mm, containing 163 breast carcinomas), Melanoma TMAs (K063Me01: array point diameter, 1.5 mm, containing 61 melanoma samples; K983501: array point diameter, 1.5 mm, containing 49 melanoma samples) and Colorectal cancer TMAs (ZM6.27H-1: array point diameter, 1.5 mm, containing 78 colorectal cancer samples; ZM6.27H-2: array point diameter, 1.5 mm, containing 71 colorectal cancer samples) were applied to measure the expression of CD1c, CD141, LAMP3, CD83 and CD11c. The samples were incubated with primary antibodies against CD1c, CD141, LAMP3, CD83 and CD11c for immumohistochemical and immunofluorescent staining. They were imaged with a slide scanner (Pannoramic DESK, 3D HISTECH, Hungary) and analyzed via Image J software.

### DC infiltration and activation in tumour-bearing mouse models

To build the subcutaneous 4T1 breast cancer models, 4T1 cells (1×10^6^ cells) were seeded on the subcutaneous second mammary glands on day 0. As for the subcutaneous B16F10 melanoma and CT26 colorectal cancer models, 1 × 10^6^ cells were seeded on the subcutaneous on day 0. On day 7 and 12, when tumour volume was up to 100–200 mm^3^ and 300–500 mm^3^, all mice were euthanized, and tumour tissues from each group of mice were collected and weighed. Then, the tumours were minced and digested for 2 h at 37 °C in 1640 medium containing hyaluronidase (0.2 mg/ml), collagenase (0.2 mg/ml), and DNAase (0.1 mg/ml). The single cell suspension was collected after filtering the digests with 70 μm cell filters, stained with Fixable Viability Dye eFluor 455 (eBioscience) and blocked with the Fc shield. Then, APC-eFlour 780-anti-Ly-6G/Ly-6C (Gr-1), Alexa Flour 700-anti-F4/80 and PE-Cy7-anti-CD326 (EpCAM) antibodies were added to exclude granulocytes, macrophages and epithelial cells; PE-anti-CD11c, APC-anti-MHC II, FITC-anti-CD172a, PerCP-Cy5.5-anti-XCR1, PerCP-Cy5.5-anti-CD11b, PerCP-Cy5.5-anti-LAMP3 and FITC-anti-CD103 antibodies were added to stain cDCs. All of the antibodies were used at a dilution of 1:200 unless otherwise noted. After staining at 4 °C for 30 min, the cells were gated on flow cytometry using a BD LSRFortessa Cell Analyzer (BD Biosciences), and data were analyzed using FlowJo V10 (TreeStar).

### Bioinformatics analysis

The Cancer Genome Atlas (TCGA) data, including breast invasive carcinoma (BRCA), colon adenocarcinoma (COAD) and uveal melanoma (UVM), were used. The survival analysis and Kaplan-Meier curve of the overall survival probability of breast cancer (BRCA) patients with high or low activated dendritic cell infiltration were analyzed using Tumour Immune Estimation Resource (TIMER). DC activity was quantified using the myeloid DC-activated scores derived from the xCell algorithm using the TIMER2.0 web platform. Patients in the top 50% of DC activity scores (*n * =  1100) were selected for all analyses. The relationship of CCL2 level and DC infiltration level was quantified using the myeloid DC scores derived from the xCell algorithm using the TIMER2.0 web platform (https://compbio.cn/timer2/). BRCA (*n* = 1100), COAD (*n* = 458) and UVM (*n* = 80) were selected for all analyses. The Survival analysis and Kaplan-Meier curve of the overall survival probability of BRCA patients with high or low cDC1 infiltration (characterized by *CD11c*, *CD141*, *XCR1* and *Clec9A*), cDC2 infiltration (characterized by *CD11c*, *CD1c*, *CD11b* and *CD172a*), *CD11c*^*+*^*LAMP3*^*+*^ and *CD83* expression, derived from TCGA and Genotype-Tissue Expression (GTEx) databases, were obtained from Gene Expression Profiling Interactive Analysis (GEPIA; http://gepia2.cancer-pku.cn/#analysis). Methods: Overall survival; Group Cutoff: Median. *p*-value was calculated by the log-rank test. The data that support these findings of this study are available.

### Cell isolation and culture

Murine BMDCs were obtained by isolating bone marrow cells from femurs and tibias of C57BL/6 or Balb/c mice, centrifuged at 300 g for 4 min and cultured in the RPMI 1640 complete growth medium containing 10% FBS, 20 ng/mL GM-CSF and 10 ng/mL IL-4. Half of the medium was replaced every 2 days. On day 6 of culture, the immature BMDCs were collected. BMDCs were purified using mouse CD11c Positive Selection Kit (Stemcell, cat. no. 18780).

DCs from peripheral blood, spleen and tumour tissues were isolated using FACS-assisted cell sorting using a BD Influx FACS. For tumour tissue, the protocol involved sequentially gating on live cells using a viability dye, identifying immune cells via CD45 expression, and excluding CD88^+^ and F4/80^+^ cells. Finally, the CD11c^+^MHC II^+^ cell population was sorted for analysis. For the spleen and blood, after excluding F4/80^+^ cells, the CD11c^+^MHC II^+^ population was isolated. CD8^+^ T cells were purified from spleen using mouse CD8^+^ T Cell Isolation Kit (Stemcell, cat. no. 19853). NK cells were purified from spleen mouse NK Cell Isolation Kit (Stemcell, cat. no. 19855).

Murine cDC1s were obtained by isolating bone marrow cells from femurs and tibias of C57BL/6 or Balb/c mice, centrifuged at 300 g for 4 min and cultured in the RPMI 1640 complete growth medium containing 10% FBS, 50 µM 2-mercaptoethanol, 200 ng/mL FTL3L and 2 ng/mL GM-CSF. The media was refreshed on days 3 and 6. Non adherent cells were collected on day 9, counted, and replated. Non adherent cells were harvested on days 12-15. Prior to use, cells were purified using mouse CD11c Positive Selection Kit (Stemcell, cat. no. 18780) and washed three times with PBS.

BMDCs were incubated in normal conditions or hypoxic (1% or 0.1% O_2_) conditions, followed by co-culturing with 4T1 cells or 4T1 cell supernatant for 24 h. To stimulate hypoxic tumour microenvironment (TME) in vitro, 4T1 cell lines (5×10^5^ cells) were seeded into 6-well plates and cultured for 24 h. Then, different DC formulations (5×10^5^ cells) were added to the upper chamber of the transwell cell culture system and co-cultured with 4T1 cells at 37 °C in hypoxia (0.1% O_2_).

Human PBMCs were isolated using human peripheral blood monocyte isolation kit (TBD2011H05, tbdscience) and cultured in the RPMI 1640 complete growth medium containing 10% FBS (Gibco), 2 mM glutamine, 100 U/ml penicillin and 100 μg/ml streptomycin, 50 ng/mL recombinant human GM-CSF (PeproTech, 300-03) and 50 ng/mL recombinant human IL-4 (PeproTech, 200-04), at a density of 2×10^6^ cells/mL for 7 days. Half of the medium was replaced every 2 days. After culturing for 6 days, immature MoDCs were obtained. Human blood T cells were isolated from peripheral blood using EasySep™ Direct Human T Cell Isolation Kit (Stemcell, cat.no. 19661).

### BMDC purity assay

To evaluate the purity of GM-CSF/IL-4- and GM-CSF/FLT3L-induced DCs, cells were resuspended in PBS and incubated with PE-anti-CD88, Pacific Blue™ anti-mouse Siglec H, APC-anti-CD370 (Clec9A), PerCP-Cy5.5-anti-XCR1, FITC-anti-CD172a (SIRP1α), APC-anti-MHC II and PE anti-CD11c at 4 °C for 30 min. The cells were collected for flow cytometry using a BD LSRFortessa Cell Analyzer (BD Biosciences) and data were analysed using FlowJo V10 (TreeStar). To evaluate the immunostimulatory capacity, GM-CSF/IL-4- and GM-CSF/FLT3L-induced DCs were incubated with OVA (10 μg/mL) and LPS (100 ng/mL) for 48 h. Then, the cells were incubated with APC anti-H-2Kb bound to SIINFEKL, APC-anti-CD80, PerCP-Cy5.5-anti-CD86, PE-anti-CD103 and PE-eFlour 610-anti-CCR7 at 4 °C for 30 min and collected for flow cytometry. To test T cell activation, spleen tissues were collected from female C57BL/6 or Balb/c mice, grinded with syringe handle, and filtered with cell strainers (70 μm, Falcon, USA). Cells were collected (400 g, 10 min), and the red blood cells were removed using lysis buffer (Yeasen, cat.no. 40401ES76) to obtain the purified splenocytes. They were seeded into the 6-well plates (1.5×10^6^ cells/well) and incubated with matured GM-CSF/IL-4- and GM-CSF/FLT3L-induced DCs (3×10^5^ cells/well). the cells were pre-coated with APC-anti-CD3, PerCP-Cy5.5-anti-CD3, FITC-anti-CD8, PE-anti-IFN-γ, PE-Cy7-anti-Gzmb, FITC-anti-CD4 and PE-anti-OVA Tertramer-SIINFEKL. The cells were then washed and resuspended in PBS for flow cytometry analysis. The secretion of IL-12p70, IL-12p40, CCL4 and CCL5 in cell culture medium or serum were assessed by ELISA kits.

### Adoptive DC transfer studies

For adoptive transfer of BMDCs to tumour-bearing mice, 4T1-OVA cells (1×10^6^ cells) were seeded on the subcutaneous second mammary glands of CD45.2 mice (*n* = 5). BMDCs were prepared from CD45.1 mice and were injected intravenously, subcutaneously or intratumourally to congenic CD45.2 tumour-bearing mice (2×10^6^ cells per mice). Recipient mice were killed 2 days after the second cell dose, and major organs and the tumour were removed to analyze donor-derived cells.

To evaluate tumour-infiltrating immune cells and the distribution of transferred BMDCs, CellTrace™ Blue-labelled BMDCs (2×10^6^ cells per mouse) were injected intravenously, subcutaneously or intratumourally. 48 h later, all mice were euthanized, and tumour tissues from each group of mice were ground into single-cell suspension with the concentration of 10^7^ cells/mL. The cells were blocked with the Fc shield according to the manufacture’s protocol. The single cell suspension was stained with PE-Cy7-anti-CD45, PerCP-Cy5.5-anti-CD3, APC-anti-CD49b, APC-anti-CD11b, PE-anti-F4/80, PE-anti-CD103, FITC-anti-CD19 and FITC-anti-CD11c. After staining at 4°C for 30 min, the cells were collected for flow cytometry. The major organs were minced and digested into single-cell suspensions and were collected to analysis CellTracker blue^+^ cells using flow cytometry.

### CCR2 expression on immune cells

The blood from BALB/c mice were collected and the red blood cells were removed using lysis buffer (Yeasen, cat.no. 40401ES76). The spleen tissues were collected, grinded and filtered to obtain singel-cell suspension. The single cell suspension was stained with PE-anti-CCR2, FITC-anti-CD8, FITC-anti-CD4, PerCP-Cy5.5-anti-CD3, FITC-anti-CD49b, FITC-anti-CD11b, PE-Cy7-anti-Ly-6G, APC-anti-F4/80, FITC-anti-CD19 and APC-anti-CD11c. After staining at 4°C for 30 min, the cells were collected for flow cytometry. CCR2-T and CCR2-NK cells were infected with recombinant CCR2 lentivirus (Hanbio Biotechnology, China) (MOI = 25) or blank lentivirus control for 48 h at 37 °C.

### Preparation and characterization of algae-derived nanovesicles (ANVs)

The algae (GY-D12 *Chlorella pyrenoidosa*) were purchased from Guangyu Biological Technology Co., Ltd. (Shanghai, China). The algae were washed with ultrapure water three times to remove any residual medium and resuspended in ultrapure water. Then, 1 × 10^7^ algae cells were treated with 50 μM DBCO-PEG_2000_-NHS ester for 45 min at room temperature (RT). To examine the effective binding of DBCO groups on the algae surface, the resulting algae were incubated with 6-Carboxyfluorescein-azide (6-FAM-N_3_, aladdin co. ltd.) for 30 min and then subjected to flow cytometry analysis and confocal imaging. The algae were then sonicated and homogenized with a probe for 5 min to prepare algae-derived nanovesicles (ANVs). The ANVs were extruded through the membrane filters with a pore size of 400 nm and 200 nm to reduce their size. For ANV purification, Amicon ultra-centrifugal filter (molecular weight cut-off=100 kDa; Millipore, Billerica, MA, USA) (10000 rpm, 15 min) was used for primary isolation and 0.22 μm filter membrane was used for secondary purification to remove vesicular debris over 200 nm. The protein content was quantified by BCA Protein Assay Kit. The particle size of algae and ANVs were measured by dynamic light scanning (DLS) (Malvern, Britain). For morphology investigation, the algae and ANVs were imaged by TEM (Tecnai F20, FEI, USA). ANVs were further imaged using cyto-TEM performed by Yanteng Biotechnology Co., Ltd. (Shanghai, China). To investigate the electron transport, algae and ANVs were mixed with DCIP (MCE, HY-D0018, 60 μM) for 1 min. After light exposure (670 nm, 600 mW/cm^2^) for specific durations (0, 5, 10, 20, 30, 45 min), the absorbance of the solution at 600 nm was measured. The chlorophyll content of ANVs is determined by UV spectrophotometry using the following formulas:

$${{{\rm{Chlorophyll\; a}}}}({{{\rm{\mu }}}}{{{\rm{g}}}}/{{{\rm{mL}}}})=1{3.95{{{\rm{A}}}}}_{665}-{6.88{{{\rm{A}}}}}_{649}$$

$${{{\rm{Chlorophyll\; b}}}}({{{\rm{\mu }}}}{{{\rm{g}}}}/{{{\rm{mL}}}})={24.96{{{\rm{A}}}}}_{649}-{7.32{{{\rm{A}}}}}_{665}$$

### Proteomics analysis

To study whether the ANVs have an independent photosynthetic function of the thylakoid organelle, we analyzed the ANVs using proteomics. Briefly, the proteins were isolated using the protein lysis buffer (8 M urea,1% SDS) and quantified by BCA Protein Assay Kit (Thermo Scientific). The proteins were then digested, and the obtained peptides were desalted and quantified. Next, the peptides were redissolved in spectrometry loading buffer (2% ACN with 0.1% formic acid), which included appropriate iRT peptide that was used to calibrate retention time and analyzed by an EASY-nLC system (Thermo, USA) coupled with a timsTOF Pro2 mass spectrometer (Bruker, German) at Majorbio Bio-Pharm Technology Co. Ltd. (Shanghai, China). Data-independent acquisition (DIA) data were acquired using a timsTOF Pro2 mass spectrometer operated in DIA-PASEF mode. Spectronaut software (Version 14) was used to search the DIA-PASEF raw data. Bioinformatic analysis of proteomic data was performed with the Majorbio Cloud platform (https://cloud.majorbio.com). The identified protein sequences were analyzed on the basis of GO terms.

### Cellular uptake of ANVs

Murine BMDCs were obtained as the aforementioned method. BMDCs were incubated in 1640 PRMI containing Ac_4_ManNAz (50 μM) for 3 days in 6-well plates (1×10^6^ cells per well). Then, ANVs (100 μg protein) were added to the medium and co-cultured with BMDCs for 30 min at 37°C. After centrifugal removal of free ANVs, CCR2-DC-ANVs were resuspended in medium. The cell samples were washed three times with PBS after an additional 0 or 6 h incubation and stained with Hoechst 33342 for 30 min. iFluor® 488-Wheat Germ Agglutinin (WGA) Conjugate (ATT Bioquest, cat.no. 25530) was used to label cell membrane after incubation for 20 min at 37 °C and LysoTracker Green DND-26 (Yeasen, cat.no. 40738ES50) was used to label lysosomes after incubation for 20 min at RT. To evaluate the location of photosynthetic proteins, the cells were incubated with anti-PsbA Dl protein (Agrisera, cat. no. AS05 084, 1:200), anti-PsaA PSI-A core protein (Agrisera, cat. no. AS06 172, 1:200) and anti-PetL Cytochrome b6-f complex subunit 6 (Agrisera, cat. no. AS22 4883, 1:200) for 1 h at 4°C and the labelled by Alexa Fluor 488-labelled goat anti-rabbit IgG (H + L) for 1 h at 4°C. Next, the cell samples were fixed with 4% paraformaldehyde (PFA) for 15 min. Finally, the cells were observed using TCS SP8 confocal microscope (Leica, Germany) and analyzed via Image J.

For the ANV uptake inhibition studies, BMDCs were pre-treated for 30 min with 20 µL of pharmacological inhibitors, including dextran sulfate (4 mg/mL), chlorpromazine (50 µM), cytochalasin D (2.5 µM) and methyl-β-cyclodextrin (Mβ-CD) (32 µM). In particular, dextran sulfate, chlorpromazine, cytochalasin D Mβ-CD are inhibitors of scavenger receptor class A-mediated endocytosis, clathrin-mediated endocytosis, micropinocytosis/phagocytosis and lipid rafts/cholesterol-enriched microdomains/caveolae. Then, ANVs (100 μg protein) were incubated with BMDCs for an additional 6 h in the absence of inhibitors. Finally, the cells were collected for flow cytometry using a BD LSRFortessa Cell Analyzer (BD Biosciences), and data were analysed using FlowJo V10 (TreeStar).

### Preparation and characterization of CCR2-DC-ANVs

Murine BMDCs were obtained as the aforementioned method. BMDCs were infected with recombinant CCR2 lentivirus (Hanbio Biotechnology, China) (MOI = 20) or blank lentivirus control 5 days after BMDC isolation for 48 h at 37 °C. Next, CCR2-BMDCs (1×10^6^ cells) were incubated in 1640 PRMI containing Ac_4_ManNAz (50 μM) for 3 days and incubated with ANVs (100 μg protein) at 37 °C for 6 h. To remove free ANVs, CCR2-DC-ANVs were centrifugated for three times (1500 rpm, 3 min) at room temperature. The final supernatant was collected and ANV-specific fluorescence (650 nm) was measured using microplate reader to evaluate the successful separation of free ANVs and CCR2-DC-ANVs. CCR2-DC-ANVs were resuspended in medium and detected by scanning electron microscope (SEM). For flow cytometry analysis, BMDCs, CCR2-DCs, CCR2-DC-N_3_ and CCR2-DC-ANVs were resuspended in PBS and incubated with PE-anti-CCR2 at 4 °C for 30 min. For confocal imaging, CCR2-DC-ANVs were incubated with CCR2 antibody, followed by staining with secondary antibody (goat anti-rabbit IgG (H + L) Alexa Fluor 488; 1:100; Yeasen). Next, CCR2-DC-ANVs were stained with Hoechst 33342, fixed with 4% paraformaldehyde (PFA), and used immediately for confocal imaging analysis. CCR2-DC-ANVs were also imaged under bright field. To test CCR2 protein on CCR2-DC-ANVs, RIPA lysis buffer was used for protein extraction. Then, SDS-PAGE gels were used to separate the extracted protein. After electrophoresis, polyvinylidene difluoride membranes were used for protein transfer. The proteins were then blocked with nonfat milk. After incubation with primary anti-CCR2 antibody, anti-GAPDH antibody and HRP-labelled goat anti-rabbit IgG (H + L), a chemiluminescent signal was achieved using detection reagents (enhanced chemiluminescence, Beyotime). To test CCL2/CCR2 interaction, CCL2 (1 ng/mL, 1 mL) was added to the culture medium of BMDCs and CCR2-DC-ANVs. 24 h later, the suspension was collected and evaluated using CCL2 ELISA kit.

### Cell viability assay

BMDCs were seeded into 96-well plates at a density of 10^4^ cells per well and incubated overnight followed by the addition of ANVs at different concentrations. After an additional 24 h of incubation, cell viability was measured using a cell counting kit (CCK-8) assay following the manufacturer’s instructions.

To clarify whether ANVs cause a cellular stress response, the intracellular reactive oxygen species (ROS) were measured. CCR2-DC-ANVs were cultured for different times (0, 4, 24, 48, 72 h) and then incubated with DCFH-DA (10 μM) for 30 min at 37°C. The cell samples were collected and washed for cytometry analysis. The stability of ANVs in CCR2-DC-ANVs were also detected using flow cytometry after culturing for different times (0, 1, 2, 4, 6, 8, 10, 24, 48 h)

### O_2_, ATP and NADPH generation of CCR2-DC-ANVs

To investigate the oxygen release profile in real time, an equal volume of BMDCs, ANVs (isolated from 10^8^ algae cells) and CCR2-DC-ANVs with a dose of 10 ml separately at a final concentration of 10^7^ cells/mL, was added into the cuvette, which was then subjected to 670 nm red light sources at a power density of 600 mW/cm^2^. Besides, the oxygen release profile of BMDCs, algae and CCR2-DC-ANVs (5 mL, 10^7^ cells/mL) was also evaluated. A portable dissolved oxygen meter was applied to record the oxygen concentrations at selected time intervals after irradiation. To test the concentration-dependent effects, BMDCs were incubated with [Ru(dpp3)] Cl2 (10 mg/L, 10 µL) for 12 h. Then, ANVs with different protein contents (0, 1, 5, 10, 20, 50, 100, 200 µg) were added to the medium and cultured with BMDCs for 6 h. After centrifugal removal of free ANVs, CCR2-DC-ANVs was resuspended in medium and subjected to red light sources at a power density of 600 mW/cm^2^ for 10 min. To test the intracellular oxygen level in hypoxic TME, [Ru(dpp3)] Cl2-labelled BMDCs and CCR2-DC-ANVs were incubated with 4T1 cells in hypoxic condition for 24 h. CCR2-DC-ANVs were exposed to 670 nm red light at a power density of 600 mW/cm^2^ for 10 min. To evaluate the effect of light duration, hypoxic CCR2-DC-ANVs were irradiated for different times (0, 10, 20, 30, 45 min). To evaluate the duration of intracellular hypoxia recovery in CCR2-DC-ANVs, irradiated CCR2-DC-ANVs (600 mW/cm^2^, 10 min) were incubated in TME for different times (0.5, 1, 2, 4, 8, 12, 24, 48 h). The cells were then observed using TCS SP8 confocal microscope (Leica, Germany). To evaluate the intracellular ROS level of CCR2-DC-ANVs, irradiated CCR2-DC-ANVs (600 mW/cm^2^, 10 min) were incubated in TME for different times (0.5, 1, 2, 4, 8, 12, 24, 48 h) and further incubated with DCFH-DA (10 μM) for 30 min at 37°C. The cell samples were collected and washed for cytometry analysis.

To test ATP and NADPH generation profile in real time, CCR2-DC-ANVs were subjected to red light sources at a power density of 600 mW/cm^2^ for different times (0, 10, 20, 30, 45 min). To test the ATP and NADPH generation in hypoxic TME, [Ru(dpp3)] Cl2-labelled BMDCs and CCR2-DC-ANVs were incubated with 4T1 cells in hypoxic condition for 24 h. CCR2-DC-ANVs were exposed to 670 nm red light at a power density of 600 mW/cm^2^ for 10 min. The intracellular ATP and NADPH concentrations were measured using assay kits.

### Transmission electron microscope (TEM) assay

Purified peripheral DCs, splenic DCs and tumour-associated DCs (TADCs) were fixed with electron microscope fixation fluid at 4 °C for 12 h. After adequate washing, samples were stained with 1% aqueous uranyl acetate. Samples were dehydrated with sequential washes in 50, 70, 90, 95 and 100% ethanol and immersed in Eponate 12 Resin. Samples were then cut into ultrathin sections and counterstained with uranyl acetate and lead citrate. Images were acquired with a transmission electron microscope (120 kV, FEI). The long sides of mitochondria in each cell were analyzed using ImageJ. The long side represents the length of a mitochondrion. The same experimental method was also applicable to BMDCs and CCR2-DC-ANVs. The endoplasmic reticulum (ER) and lipid droplets (LDs) were also observed and labelled.

### Mitochondrial assay

Purified peripheral DCs, splenic DCs and tumour-associated DCs (TADCs) were collected and resuspended to single cell suspension. Hypoxic BMDCs and CCR2-DC-ANVs (cells co-cultured with 4T1 cells in hypoxic condition) were subjected to red light (600 mW/cm^2^, 10 min) and cultured for another 24 h. Mitochondrial mass and membrane potential were analyzed using MitoTracker Green (10 nM) and TMRM (50 nM) for 30 min at 37 °C before flow cytometry analysis, respectively. For measurement of mitochondrial superoxide production, fresh cells were incubated with 10 μM MitoSOX. After washing twice with PBS, cells were processed for flow cytometry. For real-time analysis of the oxygen consumption rate (OCR), fresh cells (2×10^5^ cells/well) were plated on poly-lysine-pretreated Seahorse plates in XF media (25 mM glucose, 2 mM glutamine and 1 mM pyruvate) and analyzed using an XFe 24 Extracellular Flux Analyzer (Agilent Technologies). Basal OCR and ECAR were measured for 30 min. Cells were treated with 2 mM oligomycin, 1.5 mM FCCP, and 1 mM rotenone and 1 mM antimycin A (XFCell Mito Stress Test Kit, Agilent Technologies, cat. no. 103015-100), to measure maximum respiration. To detect cytosolic and mitochondrial calcium ion concentration, the cells were collected and incubated with Fluo-4/AM (5 µM) or Rhod-2/AM (10 µM) for at 37 °C for 30 min. Then, the cells were washed for flow cytometry analysis. To analyze intracellular phosphorylation of Drp1 Ser616, BMDCs and hypoxic BMDCs were collected and stained with anti-phospho-DRP1^S616^ at 4 °C for 1 h. Then, the cells were washed and incubated with Multi-rAb CoraLite Plus 555-Goat Anti-Rabbit Recombinant Secondary Antibody (H + L). Then, the cells were washed for flow cytometry analysis and confocal imaging. For Drp1 knockdown, siRNA-Drp1 (Santa Cruz Biotechnology, cat. no. sc-43732, 10 μM) and siRNA-mock were transfected by Lipofectamine 2000 (Invitrogen, cat. no. 11668-027) into BMDCs. After 6 h incubation, the transfected BMDCs were collected and cultured under hypoxic or normoxic systems for 24 h and used for immunofluorescence assay and flow cytometry analysis. To analysis the role of PSI and PSII in CCR2-DC-ANVs, algae cells (1 × 10^7^ cells/mL, 1 mL) were incubated with Simazine (400 µM) or Diquat (1 mM) for 12 h at room temperature. Then, the cells were collected and sonicated to ANVs. Finally, CCR2-DC-ANVs were fabricated and evaluated as described above. To measure the expression of mitochondrial functional protein, the protein of BMDCs, hypoxic BMDCs and CCR2-DC-ANVs were extracted and separated on polyvinylidene difluoride membranes. The proteins were then blocked with nonfat milk. After incubation with primary anti-OPA1, anti-ATP5A1, anti-UQCRC2, anti-SDHB, anti-NDUFB, anti-β-actin antibodies and HRP-labelled goat anti-rabbit IgG (H + L), a chemiluminescent signal was achieved using detection reagents (enhanced chemiluminescence, Beyotime). To measure the expression of mitochondrial fusion and fission protein, primary anti-Drp1, anti-phospho-DRP1^S616^, anti-phospho-DRP1^S637^, anti-MFN1, anti-MFN2 and anti-β-actin antibodies were used. To measure the expression of protein related to Akt1-mTOR-HIF-1α signalling, anti-phospho-mTOR^S2448^, anti-phospho-AKT1^S473^, HIF-1α and anti-β-actin antibodies were used.

### ER and LD assay

Purified peripheral DCs, splenic DCs and tumour-associated DCs (TADCs) were collected and resuspended to single cell suspension. Hypoxic BMDCs and CCR2-DC-ANVs were subjected to red light (600 mW/cm^2^, 10 min) and cultured for another 24 h. ER stress and LD accumulation were analyzed using ER-Tracker Green (10 μM) and BODIPY 493/503 (2 µg/mL) for 30 min at 37 °C before flow cytometry analysis and confocal imaging, respectively. For measurement of TAG content, the cells were sonicated and homogenized in PBS. The cell lysate was mixed with CHCl_3_-CH_3_OH (2:1, v/v) for the extraction of organic phase. The organic phase was air-dried overnight and re-suspended in ethanol containing 1% TritonX-100. The Triacylglycerol (TAG) concentration was quantified with triglyceride reagent (Sigma-Aldrich, cat. no. TR0100). To measure the expression of ER stress-related protein, primary anti-PERK, anti-phospho-eIF2α^S51^, anti-eIF2α, anti-phospho-IRE1^S724^, anti-XBP-1S, anti-XBP-1U and anti-β-actin antibodies were used.

### Cell apoptosis assay

Purified peripheral DCs, splenic DCs and TADCs were collected and resuspended to single cell suspension. Hypoxic BMDCs and CCR2-DC-ANVs were subjected to red light (600 mW/cm^2^, 10 min) and cultured for another 24 h. The cells were stained using Annexin V-FITC Apoptosis Detection Kit for 20 min at room temperature for flow cytometry analysis. To evaluated the apoptosis of irradiated CCR2-DC-ANVs in vivo, CellTrace™ Blue-labelled BMDCs, CCR2-DCs and CCR2-DC-ANVs (2×10^6^ cells per mouse) were administrated via *i.v*. injection. After 24 h, continuous 2% isoflurane at a flow rate of 2 liters/min was delivered to the mice, followed by red-light irradiation (670 nm, 10 min). 24 h later, all mice were euthanized, and tumour tissues were ground into single-cell suspension and stained using Annexin V-FITC Apoptosis Detection Kit for flow cytometry analysis. To measure the expression of apoptosis-related protein, primary anti-cleaved caspase 3, anti-caspase 7, anti-eIF2α and anti-β-actin antibodies were used.

### Antigen presentation capacity of CCR2-DC-ANV

Hypoxic BMDCs and CCR2-DC-ANVs (cells co-cultured with 4T1-OVA cells in hypoxic condition) were subjected to red light (600 mW/cm^2^, 10 min) and cultured for another 24 h. The cells were then collected and stained using APC/PE-anti-H-2Kb bound to SIINFEKL for flow cytometry analysis. To evaluate the effect of light duration, hypoxic CCR2-DC-ANVs were irradiated for different times (0, 10, 20, 30 min). The cells were collected at different times (1, 3, 5 days) post-irradiation. To evaluate the impact of LD accumulation on antigen presentation, CCR2-DC-ANVs were incubated with Avasimibe (100 nM) or Lipofermata (50 nM) for 24 h. To evaluate the role of mitochondria and ER on antigen presentation, CCR2-DC-ANVs were incubated with Mdivi-1 (10 nM), si-Drp1 (10 μM), Rapmycin (20 nM), 4-PBA (1 mM) and GSH (1 mM) for 24 h. Then, the cells were incubated in hypoxic TME for 24 h and irradiated for 10 min, followed by another 24 h incubation. MitoTracker Green, ER-Tracker Green and BODIPY 493/503 were used to analyze the mass of mitochondria, ER and LD. The cells were then collected and stained using APC/PE-anti-H-2Kb bound to SIINFEKL for flow cytometry analysis.

### Transcriptomics study

For the transcriptomics study, total RNA of BMDCs and CCR2-DC-ANVs was extracted from tissues using TRIzol® Reagent according the manufacturer’s instructions. The RNA-seq transcriptome library was prepared following Illumina® Stranded mRNA Prep, Ligation (San Diego, CA) using 1 μg of total RNA. The sequencing library was performed on DNBSEQ-T7 platform (PE510) using DNBSEQ-T7RS Reagent Kit (FCL, PE510). The differently expressed genes were detected and analyzed by Shanghai Majorbio Bio-pharm Technology Co., Ltd. (https://www.majorbio.com). Genes with *p*  <  0.05 and absolute log_2_(fold changes) ≥ 1 were identified as differentially expressed genes. GO enrichment analysis of differentially expressed genes was implemented using the hypergeometric test. To determine the level of mitochondrial metabolic pathway enrichment, we used gene set enrichment analysis (GSEA) to compare the pathways between different groups.

### Immune activation effect of CCR2-DC-ANV in vitro

To evaluate CD83 expression in matured BMDCs, BMDCs were treated with LPS (10 μg/mL) for 48 h and collected for flow cytometry analysis. APC-anti-CD83 antibody was used to label activated BMDCs.

To test the maturation of CCR2-DC-ANV after incubation with 4T1 cell lines, Hypoxic BMDCs and CCR2-DC-ANVs (cells co-cultured with 4T1-OVA cells in hypoxic condition) were subjected to red light (600 mW/cm^2^, 10 min) and cultured for another 24 h. The BMDCs or CCR2-DC-ANVs on the upper chamber and medium were collected and incubated with PE-anti-CD80, APC-anti-CD86, PE-anti-CD40, APC-anti-MHC-I and PE-anti-MHC-II antibodies respectively at 4 °C for 30 min for flow cytometry analysis.

To test T cell proliferation and activation, purified splenocytes were collected and stained with CFSE as described above. They were seeded into the 6-well plates (1.5×10^6^ cells/well) and incubated with BMDCs and CCR2-DC-ANVs (3×10^5^ cells) after incubation with 4T1-OVA and red-light irradiation. After 48 h incubation, the cells were collected and stained with PerCP-Cy5.5-anti-CD3. Then, the cells were evaluated by the CFSE dilution analysis in flow cytometry. For T cell activation, the cells were pre-coated with APC-anti-CD3, PerCP-Cy5.5-anti-CD3, PE-anti-CD8, FITC-anti-IFN-γ, PE-Cy7-anti-GzmB, PE-anti-OVA Tertramer SIIFEKL, APC-anti-CD366, APC-anti-CD223, APC-anti-CD279, FITC-anti-CD4, PE-anti-IFN-γ, PE-anti-IL-4, PE-anti-IL-17A and PE-anti-Foxp3. The cells were then washed and resuspended in PBS for flow cytometry analysis. The secretion of IL-12p70, IFN-γ and TNF-α in cell culture medium or serum were assessed by ELISA kits.

To evaluate the role of toll-like receptor (TLR) in CCR2-DC-ANV activation, CCR2-BMDCs and BMDCs were treated with C29 (50 μM, TLR-2 inhibitor, MedChemExpress, cat.no. HY-100461) and resatorvid (100 nM, TLR-4 inhibitor, MedChemExpress, cat.no. HY-11109) for 4 h at 37°C. Then, CCR2-BMDCs were incubated with ANVs to construct CCR2-DC-ANVs and co-cultured with 4T1-OVA as the aforementioned method. For western blot analysis, primary anti-IRAK1, anti-NF-κB p65, anti-MAPK p38, anti-MyD88, anti-β-actin antibodies and HRP-labelled goat anti-rabbit IgG (H + L) were used and analyzed as the aforementioned method.

### In vivo biodistribution assay

To build the subcutaneous 4T1 breast cancer models, 4T1 cells (1×10^6^ cells) were seeded on the subcutaneous second mammary glands. Purified CD8^+^ T cells and NK cells were infected with recombinant CCR2 lentivirus (Hanbio Biotechnology, China) (MOI = 30) or blank lentivirus for 48 h at 37 °C. Then, the cells were labelled using DiR dye and injected intravenously into 4T1 tumour-bearing mice. The tumour tissues were collected for determining ex vivo at 24 h after the injection (*n* = 3). To evaluate the distribution of CCR2-DCs, DiR-labelled BMDCs and CCR2-DCs (DiR, 1 mg/kg, 2×10^6^ cells per mouse) were administrated to mice via *i.v*. injection. The tumour tissues were collected for determining ex vivo at 24 h after the injection (*n* = 3). To analysis the role of CCL2 in CCR2-DC recruitment, 4T1-bearing wild type (WT) mice, *Ccl2* knockout 4T1-bearing WT mice, 4T1-bearing *Ccl2*^*−/−*^ mice and *Ccl2* knockout 4T1-bearing *Ccl2*^*−/−*^ mice models were established. DiR-labelled CCR2-DCs (2 × 10^6^ cells per mouse) were administrated to mice via *i.v*. injection. The tumour tissues were collected for determining ex vivo at 24 h after the injection (*n* = 3). To analysis the distribution of CCR2-DC-ANVs, DiI-labelled BMDCs and CCR2-DC-ANVs (DiI, 1 mg/kg, 2 × 10^6^ cells per mouse) were administrated to mice via *i.v*. injection. The tumour tissues, lymph nodes and major organs were collected for determining ex vivo at 24, 48, 96 h after the injection (*n* = 3). Fluorescence images were carried out by IVIS (Perkinelmer, America). Tumour tissues and tumour-derived lymph nodes were sectioned into 10 μm slices. The nuclei were stained with DAPI. The blood vessels were stained with anti-CD31 antibody. The sections were observed using TCS SP8 confocal microscope (Leica, Germany). To explore the distribution of ANVs, DiI-labelled CCR2-DC-ANVs (DiI, 1 mg/kg, 2 × 10^6^ cells per mouse) were administrated and analyzed as the aforementioned method.

### Immunological efficacy of CCR2-DC-ANV in vivo

To test the activation of CCR2-DC-ANV in vivo, 4T1-OVA cells (1 × 10^6^ cells) were seeded on the subcutaneous second mammary glands. When tumour volumes were up to ~100 mm^3^, CellTrace™ Blue-labelled BMDCs, CCR2-DCs and CCR2-DC-ANVs (2×10^6^ cells per mouse) were administrated via *i.v*. injection. After 24 h, continuous 2% isoflurane at a flow rate of 2 liters/min was delivered to the mice followed by red-light irradiation (670 nm, 10 min). 48 h later, all mice were euthanized, and tumour tissues from each group of mice were ground into single-cell suspension with the concentration of 10^7^ cells/mL. The cells were blocked with the Fc shield according to the manufacture’s protocol. The different antibodies (PE-anti-CD40, PerCP-Cy5.5-anti-CD86, PE-anti-CD80, PE-anti-MHC-II, FITC-anti-MHC-I and APC-anti-SIINFEKL H-2Kd) were added to the cell suspension to stain CellTrace™ Blue-labelled cells. Besides, the inherent DCs in tumour tissues were also evaluated.

To test the immunological efficacy of CCR2-DC-ANV, subcutaneous 4T1 breast cancer models were built as before. When tumour volumes were up to ~100 mm^3^, mice were equally divided into 8 groups: PBS, CCR2-DC (2 × 10^6^ cells per mouse), BMDCs (2 × 10^6^ cells per mouse), ANVs + L (200 μg per mouse, 670 nm laser for 10 min), DC-ANVs + L (2 × 10^6^ cells per mouse, 670 nm laser for 10 min), CCR2-DC + ANVs + L (2 × 10^6^ cells per mouse, 670 nm laser for 10 min), CCR2-DC-ANVs (2 × 10^6^ cells per mouse) and CCR2-DC-ANVs + L (2×10^6^ cells per mouse, 670 nm laser for 10 min). In brief, they were administrated via *i.v*. injection on day 7 and day 10 with red laser (670 nm) for 10 min on day 8 and day 11. At the 14th day of therapy, all mice were euthanized, and tumour tissues from each group of mice were minced and digested to a single cell suspension as described above and stained with Fixable Viability Dye eFluor 455 (eBioscience) and blocked with the Fc shield. Then, APC-anti-CD45, PE-Cy7-anti-Ly-6C, Alexa Flour 700-anti-CD11c, FITC-anti-MHC II, PE-anti-CD103 and PerCP-Cy5.5-anti-CD11b antibodies were added to stain cDC1 and cDC2 cells; APC-anti-CD45, PE-anti-CD11c, PE-Cy7-anti-CD80 and APC-Cy7-anti-CD86 antibodies were added to stain matured DCs; APC-anti-CD45, PerCP-Cy5.5-anti-CD3, PE-anti-CD8, FITC-anti-IFN-γ and PE-Cy7-anti-GzmB antibodies were added to stain CD8^+^ T cells; APC-anti-CD45, PerCP-Cy5.5-anti-CD3, FITC-anti-CD4, PE-anti-IFN-γ, PE-anti-IL-4, PE-anti-IL-17A and PE-anti-Foxp3 antibodies were added to stain CD4^+^ T cells; APC-anti-CD45, PerCP-Cy5.5-anti-CD11b, PE-anti-F4/80, FITC-anti-CD86 and PE-Cy7-anti-CD206 antibodies were added to stain tumour-associated macrophages. For cytokine staining, cells were restimulated with 50 ng/mL phorbol 12-myristate 13-acetate (yeasen), 1 μg/mL ionomycin (yeasen) and 5 mg/mL brefeldin A solution (yeasen) in RPMI 1640 medium supplemented with 10% FBS and penicillin-streptomycin for 4 h. All of the antibodies were used at a dilution of 1:200 unless otherwise noted. Specifically, IFN-γ, IL-4, IL-17A, Foxp3 and CD206 staining required rupture of the cell membrane first. After staining at 4 °C for 30 min, the cells were gated on flow cytometry using a BD LSRFortessa Cell Analyzer (BD Biosciences) and data were analysed using FlowJo V10 (TreeStar).

To test the migration and antigen presentation of migratory cDCs, experiments were performed as described above. PE-Cy7-anti-CD45, APC-anti-CD3, PerCP-Cy5.5-anti-CD8, PE-anti-OVA Tertramer SIINFEKL, FITC-anti-SIINFEKL H-2Kd, PE-anti-CD11c, APC-anti-MHC II, BV605-anti-XCR1, PerCP-Cy5.5-anti-CCR7 and FITC-anti-CD172a antibodies were used.

For cytokine analysis, tumours were weighed and homogenized with 1 mL of PBS. Then, the homogenate was centrifuged, and the supernatant was obtained. The content of IL-12, TNF-α and IFN-γ in tumours was measured with the corresponding ELISA kits.

For immunostaining, the tumours were cyto-sectioned, stained with anti-fibronectin, anti-CD31 and anti-CD8 antibodies, followed by staining with secondary antibody (goat anti-rabbit IgG (H + L) Alexa Fluor 488; 1:100; Yeasen). The sections were observed using TCS SP8 confocal microscope (Leica, Germany).

For RNA-Seq analysis, 4T1 tumour-bearing mice were treated and euthanized on day 14, and tumours were isolated and sent for RNA-Seq analysis. The differently expressed genes were detected and analyzed by Shanghai Majorbio Bio-pharm Technology Co., Ltd. (https://www.majorbio.com). Genes with *p*  <  0.05 and absolute log_2_(fold changes) ≥ 1 were identified as differentially expressed genes. GO enrichment analysis of differentially expressed genes was implemented using the hypergeometric test. GSEA was used to compare the immune activation between different groups.

### Therapeutic efficacy of CCR2-DC-ANV in vivo

To test the therapeutic effect of matured GM-CSF/IL-4- and GM-CSF/FLT3L-induced DCs, 4T1 cells (1×10^6^ cells) were seeded on the subcutaneous second mammary glands at day 0. Mice were equally divided into 3 groups (*n* = 6): PBS, GM-CSF/IL-4-DC (2 × 10^6^ cells per mouse) and GM-CSF/FLT3L DC (2 × 10^6^ cells per mouse. In brief, they were administrated via *s.c*. injection on day 7 and day 10. Later, the tumour size (L: length of longest axis; W: length of shortest axis) and weight were monitored every 3 days. Tumour volumes were calculated by the following formulas:

$${{{\rm{Tumour\; volume}}}}={{{\rm{L}}}}\times {{{\rm{W}}}}\times {{{\rm{W}}}}/2$$

To test the therapeutic effect of CCR2-DC-ANVs in the breast cancer models, 4T1 cells (1 × 10^6^ cells) were seeded on the subcutaneous second mammary glands at day 0. Mice were equally divided into 8 groups (*n* = 6): PBS, CCR2-DC (2 × 10^6^ cells per mouse), BMDCs (2 × 10^6^ cells per mouse), ANVs + L (200 μg per mouse, 670 nm laser for 10 min), DC-ANVs + L (2 × 10^6^ cells per mouse, 670 nm laser for 10 min), CCR2-DC+ANVs + L (2 × 10^6^ cells per mouse, 670 nm laser for 10 min), CCR2-DC-ANVs (2 × 10^6^ cells per mouse) and CCR2-DC-ANVs + L (2 × 10^6^ cells per mouse, 670 nm laser for 10 min). In brief, they were administrated via *i.v*. injection on day 7 and day 10 with red laser (670 nm) for 10 min on day 8 and day 11. Later, the tumour size (L: length of longest axis; W: length of shortest axis) and weight were monitored every two days.

The maximal tumour volume allowed was 1500 mm^3^. In some cases, this limit has been exceeded on the last day of measurement, and the mice were immediately euthanized. Mouse lung tissues were then collected and washed with PBS, and then fixed with 4% PFA. Metastatic nodules in fixed lung tissue were counted, and the lung tissues were then embedded and sectioned for H&E staining.

To evaluate the contribution of inherent cDC1s to the therapeutic efficacy of CCR2-DC-ANVs, B16F10 cells (1 × 10^6^ cells) were seeded on the subcutaneous of C57BL/6 or *Batf3*^*−/−*^ mice at day 0. CCR2-DC-ANVs + L (2 × 10^6^ cells per mouse) were intravenously injected on day 7 and day 10 with red laser (670 nm) for 10 min on day 8 and day 11 (*n* = 5 for PBS group and *n* = 8 for CCR2-DC-ANV + L group). The tumour size was monitored every three days. The tumour tissues were collected and digested to a single-cell suspension for cell-surface staining and flow cytometry analysis.

To evaluate the contribution of T cells and macrophages to the therapeutic efficacy of CCR2-DC-ANVs, mice were injected *i.p*. with 200 μg (in 100 μl PBS) of anti-mouse CD4 (GK1.5, BioXcell, cat.no. BE0003), anti-mouse CD8α (53-6.7, BioXcell, cat.no. BE0004) and anti-mouse CSF1R (AFS98, BioXcell, cat.no. BE0213) antibodies to deplete CD4^+^ T, CD8^+^ T cells and macrophages, respectively. The rat IgG2b (LTF-2, BioXcell, cat. no. BE0009) was used as an isotype control. In the subcutaneous 4T1 model, depletion antibodies (CD4, CD8α, CSF1R) were injected 2 days after tumour inoculation and then every 3 days for a total of 6 times. (*n* = 6). Besides, B16F10 cells (1×10^6^ cells) were seeded on the subcutaneous of *Rag3*^*−/−*^ mice at day 0. CCR2-DC-ANVs + L (2 × 10^6^ cells per mouse) were intravenously injected on day 7 and day 10 with red laser (670 nm) for 10 min on day 8 and day 11 (*n* = 5). The tumour size was monitored every three days.

To evaluate the combination therapeutic efficacy of CCR2-DC-ANV with anti-mouse PD-L1 antibody, mice were injected *i.p*. with 200 μg (in 100 μl PBS) anti-mouse PD-L1 (10 F.9G2, BioXcell, cat.no. BE0101). Rat IgG2b (LTF-2, BioXcell, cat. no. BE0009) was used as an isotype control. Antibodies were injected at day 7 and then every 3 days for a total of 4 times (*n* = 5 for PBS and anti-PD-L1 group and *n* = 10 for CCR2-DC-ANV and CCR2-DC-ANV + anti-PD-L1 group). In another study, the 11 mice in the CCR2-DC-ANV + anti-PD-L1 group with a complete response (absence of detectable tumour) were re-challenged with 4T1 cells (5 × 10^5^ cells, subcutaneously) and tumour volume was recorded every three days. To evaluate the immune memory effect of the combined therapy, all mice were euthanized 30 days post treatment and spleen tissues from each group of mice were minced and digested into single cell suspension for central memory T cell (CD44^+^CD62L^+^) and effect memory T cell (CD44^+^CD62L^-^) analysis using flow cytometry as the aforementioned method.

To evaluate the antitumour efficacy of CCR2-DC-ANV in abdominal dissemination colorectal cancer model, Balb/c mice were inoculated with CT26-Luc cells (5 × 10^5^ cells) by *i.p*. injection at day 0. Mice were equally divided into 5 groups (*n* = 6): PBS, CCR2-DC (2 × 10^6^ cells per mouse), ANVs + L (200 μg per mouse, 670 nm laser for 10 min), CCR2-DC + ANVs + L (2 × 10^6^ cells per mouse, 670 nm laser for 10 min), CCR2-DC-ANVs + L (2 × 10^6^ cells per mouse, 670 nm laser for 10 min). In brief, they were administrated via *i.v*. injection on day 7 and day 10 with red laser (670 nm) for 10 min on day 8 and day 11. Tumour growth was monitored by bioluminescence imaging on an IVIS Spectrum Imaging System. Mice were considered dead when the mice had become moribund. In another study, all mice were euthanized at day 15 and tumour tissues from each group of mice were minced and digested into single cell suspension for flow cytometry analysis as the aforementioned method.

### Therapeutic efficacy of CCR2-DC-ANV compared to DC vaccine

To manufacture DC vaccine in vitro, Monocytes derived from mouse bone marrow were plated on 6-well cell culture plates at 1 × 10^6^ cells per well to generate BMDCs as the aforementioned method. Then, BMDCs were co-incubated with autologous tumour cell lysates (4T1, 4T1-OVA or B16-OVA) for 24 h and further matured with LPS (10 μg/mL) for another 24 h to obtain the DC vaccines. The antigen presentation of DC vaccines was evaluated using flow cytometry analysis.

To evaluate the therapeutic efficacy of CCR2-DC-ANV and DC vaccine, 4T1-OVA or B16-OVA cells (1 × 10^6^ cells) were seeded on the subcutaneous at day 0. Mice were equally divided into 3 groups (*n* = 5 or 6): PBS, DC vaccine (2 × 10^6^ cells per mouse) and CCR2-DC-ANVs + L (2 × 10^6^ cells per mouse). They were administrated via *i.v*. injection on day 7 and day 10 with red laser (670 nm) for 10 min on day 8 and day 11. In another study, B16-OVA cells (1 × 10^6^ cells) were injected subcutaneously into C57BL/6 mice at day 0 to establish subcutaneous melanoma model. The tumour size was monitored every two days.

To evaluate the therapeutic efficacy against distal metastatic tumours, primary tumours (4T1, 1 × 10^6^ cells) were inoculated on the right abdomen on day 0, and on day 5, distal metastatic tumours (4T1, 5 × 10^5^ cells) were subcutaneously injected into the left abdomen of Balb/c mice. Mice were randomly divided into 5 groups (*n*  =  7): PBS, DC vaccine (B16-OVA, 2 × 10^6^ cells per mouse), DC vaccine (4T1, 2 × 10^6^ cells per mouse), DC vaccine (4T1, 4 × 10^6^ cells per mouse) and CCR2-DC-ANVs + L (2 × 10^6^ cells per mouse) and the primary tumours were treated as described above. The tumour size was monitored every two days. The maximal tumour volume allowed was 1500 mm^3^. In some cases, this limit has been exceeded on the last day of measurement, and the mice were immediately euthanized.

To evaluate the systemic anti-metastasis effect, a disseminated 4T1 tumour-bearing mouse model was established. Briefly, 4T1 cells (1 × 10^6^) were injected subcutaneously into the right abdomen at day 0. The mice were randomly divided into 5 groups (*n*  =  5), and the primary tumours were treated as described above. Meanwhile, 4T1-GFP cells (5 × 10^5^) were injected intravenously into the mice at day 5. On day 27, mouse lung tissues were collected and washed with PBS and then fixed with 4% PFA. Metastatic nodules in fixed lung tissue were counted, and the lung tissues were then embedded and sectioned for H&E staining. In another study, all mice were euthanized at day 15 and spleen tissues from each group of mice were minced and digested into a single cell suspension for central memory T cell (CD44^+^CD62L^+^) and effector memory T cell (CD44^+^CD62L^-^) analysis using flow cytometry as the aforementioned method.

To evaluate the therapeutic efficacy against metastatic tumours, Balb/c mice were inoculated with 4T1-Luc cells (5 × 10^5^ cells) by *i.v*. injection at day 0. Mice were equally divided into 4 groups (*n* = 6): PBS, DC vaccine (2 × 10^6^ cells per mouse), DC vaccine (4 × 10^6^ cells per mouse) and CCR2-DC-ANVs + L (2 × 10^6^ cells per mouse, 670 nm red laser for 10 min). They were administrated via *i.v*. injection on day 10 and day 13 day with red laser (670 nm) for 10 min on day 11 and day 14. Tumour growth was monitored by bioluminescence imaging on an IVIS Spectrum Imaging System. Mice were considered dead when the mice had become moribund. Mouse lung tissues were collected, and the metastatic nodules were counted. The lung tissues were then embedded and sectioned for H&E staining.

To compare the therapeutic efficacy of CCR2-DC-ANVs and cDC1-based vaccines, cDC1s were generated as described above. The obtained cDC1s were characterized by surface marker expression, including CD11c, B220, MHC II, CD103 and Clec9A via antibody staining followed by flow cytometry. The T cell activation effect of moDCs and cDC1s was evaluated by flow cytometry analysis. CCR2-cDC1-ANVs were fabricated as described above. 4T1-OVA cells (1 × 10^6^ cells) were seeded on the subcutaneous at day 0. Mice were equally divided into 4 groups (*n* = 6): PBS, cDC1 vaccine (2 × 10^6^ cells per mouse), CCR2-DC-ANVs + L (2 × 10^6^ cells per mouse) and CCR2-cDC1-ANVs + L (2 × 10^6^ cells per mouse). They were administrated via *i.v*. injection on day 7 and day 10 with red laser (670 nm) for 10 min on day 8 and day 11. The tumour size was monitored every three days.

### In vivo biosafety study

Healthy mice were randomized into 8 groups and administrated as the same methods of the in-vivo antitumour effect study. At day 20, the mice were sacrificed. Hearts, livers, spleens, lungs, and kidneys were harvested, fixed with paraformaldehyde, dehydrated, and sliced for the H&E staining assay.

To evaluate the immunogenicity of ANVs and CCR2-DC-ANVs, ANVs and CCR2-cDC1-ANVs were administrated via *i.v*. injection. The mice were euthanized at different times post-treatment (0, 8, 24, 48, 96, 168, 336 h) (*n* = 3). The livers and lungs were harvested, fixed with paraformaldehyde, dehydrated, and sliced for the H&E staining assay. The spleen was collected and weighed. IFN-γ^+^ CD4^+^ T cells and IFN-γ^+^ CD8^+^ T cells were evaluated by flow cytometry analysis. The serum from different groups was collected and used for IL-6, IgG and IgM measurement via ELISA kits. To test the binding effect of anti-ANV antibodies in serum, 50 μL serum was added to algae cells (2 × 10^6^ cells) and incubated for 1 h at 4 °C. Then, algae cells were collected and stained using FITC-anti-IgG antibody before flow cytometry analysis. To test the immune memory of anti-ANV effect, mice were treated with ANV and CCR2-DC-ANV for 14 days, and the splenic T cells were isolated and seeded in 6-well plates (1 × 10^6^ cells per well). 50 μL ANVs were added to the medium and incubated with T cells for 24 h. Then, T cells were isolated and analyzed using flow cytometry. To perform high-resolution immunopeptidomics, CCR2-DC-ANVs (2 × 10^7^ cells) were prepared and cultured at 37 °C for 48 h. The cells were collected and frozen at −80 °C. Finally, the samples were sent to Biotech Pack Scientific Co., Ltd. (Beijing, China) for the following analysis.

### Manufacturing CCR2-DC-ANV from human PBMCs

Peripheral blood samples from healthy donor were obtained with written informed consent from all participants and ethical approval from the Ethics Committee of Shanghai General Hospital (approval number 2025SQ066). Human PBMC-derived MoDCs were generated as described above. To construct CCR2-DC-ANV, immature MoDCs were infected with recombinant CCR2 lentivirus (Hanbio Biotechnology, MOI = 20) for 48 h at 37 °C. Next, CCR2-MoDCs (1 × 10^6^ cells) were incubated in 1640 PRMI containing Ac_4_ManNAz (50 μM) for 3 days and incubated with ANVs (100 μg protein) at 37 °C for 6 h. After centrifugal removal of free ANVs, CCR2-DC-ANVs were resuspended in medium for in vitro and in vivo use. CCR2-DC-ANVs were incubated with anti-CCR2 (Affinity: cat. no. DF7507, 1:200) antibody and secondary antibody (donkey anti-rabbit IgG (H + L) Alexa Fluor 594; 1:200; Yeasen) for confocal imaging.

To evaluate the immune activation of CCR2-DC-ANV, MDA-MB-231 cell lines (5 × 10^5^ cells) were seeded into 6-well plates and cultured for 24 h. Then, MoDCs and CCR2-DC-ANVs (5 × 10^5^ cells) were added to the upper chamber of the transwell cell culture system and co-cultured with MDA-MB-231 cells at 37 °C in hypoxia (0.1% O_2_) for 24 h. Then, the cells were subjected to red light sources at a power density of 600 mW/cm^2^ for 10 min and cultured for another 24 h. The MoDCs or CCR2-DC-ANVs on the upper chamber and medium were collected for flow cytometry analysis. For T cell proliferation and activation analysis, T cells were seeded into the 6-well plates (1.5 × 10^6^ cells/well) and incubated with MoDCs and CCR2-DC-ANVs (3 × 10^5^ cells) after incubation with MDA-MB-231 cells and red-light irradiation. T cells were stained with CFSE. After 48 h incubation, T cells were collected and stained with antibodies for flow cytometry analysis as the aforementioned method.

### Therapeutic efficacy of CCR2-DC-ANV in humanized mice model

Humanized NSG mice were generated by PBMCs (1 × 10^7^ cells) were *i.v*. injected in NSG mice at day -21 to reconstruct the human immune system. The immune reconstruction efficiency (hCD45 and hCD3) was evaluated every 7 days after PBMC injection by cytometry.

To evaluate the antitumour efficacy of CCR2-DC-ANV in orthotopic breast cancer-bearing humanized mice, MDA-MB-231 cells (2 × 10^6^ cells) were seeded on the subcutaneous second mammary glands of humanized NSG mice at day 0. Mice were randomly divided into 3 groups (*n* = 5): PBS, DC vaccine (2 × 10^6^ cells per mouse) and CCR2-DC-ANV + L (2 × 10^6^ cells per mouse, 670 nm laser for 10 min). In brief, they were administrated via *i.v*. injection on day 14 and day 17 day with red laser (670 nm) for 10 min on day 15 and day 18. Later, the tumour size and weight were monitored every two days. The maximal tumour volume allowed was 1500 mm^3^. In some cases, this limit was exceeded on the last day of measurement, and the mice were immediately euthanized. In another study, all mice were euthanized at day 22 and tumour tissues from each group of mice were minced and digested into single cell suspension for flow cytometry analysis as the aforementioned method.

To evaluate the antitumour efficacy of CCR2-DC-ANV in colorectal cancer-bearing humanized mice, HCT-116 cells (2×10^6^ cells) were seeded on the subcutaneous of humanized NSG mice at day 0. Mice were randomly divided into 3 groups (*n* = 5): PBS, DC vaccine (1 × 10^6^ cells per mouse) and CCR2-DC-ANV + L (1 × 10^6^ cells per mouse, 670 nm laser for 10 min). In brief, they were administrated via *i.v*. injection on day 14 and day 17 day with red laser (670 nm) for 10 min on day 15 and day 18. Later, the tumour size and weight were monitored every two days. The maximal tumour volume allowed was 1500 mm^3^. In some cases, this limit was exceeded on the last day of measurement, and the mice were immediately euthanized.

### Statistical analysis

Proteomics analysis and transcriptomics study were performed using the free online platform of majorbio choud platform (cloud.majorbio.com). For quantitative analysis, a minimum of three biological replicates were analyzed. Statistical analysis was conducted using GraphPad Prism 8.0.1 statistical analysis software. All values and error bars are the mean ± s.d. unless otherwise indicated. Comparisons of two groups were performed using unpaired or paired two-tailed Student’s *t*-tests. For multiple comparisons, one-way ANOVA with a Tukey’s post hoc test was used when more than two groups were analyzed, and two-way ANOVA with a Bonferroni post hoc test was used when two parameters were considered. Survival curves were analyzed using a log-rank (Mantel–Cox) test. A *P* value less than 0.05 was considered significant.

### Reporting summary

Further information on research design is available in the Nature Portfolio Reporting Summary linked to this article.

## Supplementary information

Supplementary Information

Description of Additional Supplementary Files

Supplementary Data 1-2

Reporting Summary

Transparent Peer Review file

## Source data

Source Data

## Data Availability

The raw transcriptomic and proteomic data used in this study are available under accession codes CRA022538, CRA022540 and OMIX016859. The remaining data supporting the findings of this study are available within the paper, its Supplementary Information and Source data and/or available from the corresponding authors upon request. Source data are provided with this paper.
