# Supplementary material for: A plant vesicle-dendritic cell chimera for enhancing cancer immunotherapy
Source: Nat Commun. 2026 May 28;17:6944. doi: 10.1038/s41467-026-73788-5 (PMC13388912; doi:10.1038/s41467-026-73788-5)
Supplement: Supplementary file 1 — Supplementary Information [file 41467_2026_73788_MOESM1_ESM.pdf]

Supplementary Information

A plant vesicle-dendritic cell chimera for enhancing cancer immunotherapy

Wenzhe Yi[1, 2], Xindi Qian[1], Wenlu Yan[1, 2], Dan Yan[1], Zhiwen Zhao[1, 2], Fang Sun[3], Qi Zhao[4, *], Dangge Wang[5, *], Yaping Li[1, 2, 6, *]

[1]State Key Laboratory of Drug Research & Center of Pharmaceutics, Shanghai Institute of Materia Medica, Chinese Academy of Sciences, Shanghai 201203, China;

[2]University of Chinese Academy of Sciences, Beijing 100049, China;

[3]Department of Gastroenterology, Xinhua Hospital, Shanghai Jiaotong University School of Medicine, Shanghai, 200092, China;

[4]Cancer Centre, Institute of Translational Medicine, Department of Biomedical Sciences, Faculty of Health Sciences, University of Macau, Taipa, Macau SAR, China;

[5]Precision Research Center for Refractory Diseases, Shanghai General Hospital, Shanghai Jiao Tong University School of Medicine, Shanghai, 201620, China;

[6]Shandong Laboratory of Yantai Drug Discovery & Bohai Rim Advanced Research Institute for Drug Discovery, Yantai, 264000, China.

[*] Corresponding authors: Prof. Qi Zhao (qizhao@um.edu.mo); Prof. Dangge Wang (dg_wang@sjtu.edu.cn); Prof. Yaping Li (ypli@simm.ac.cn).

Supplementary Figure s

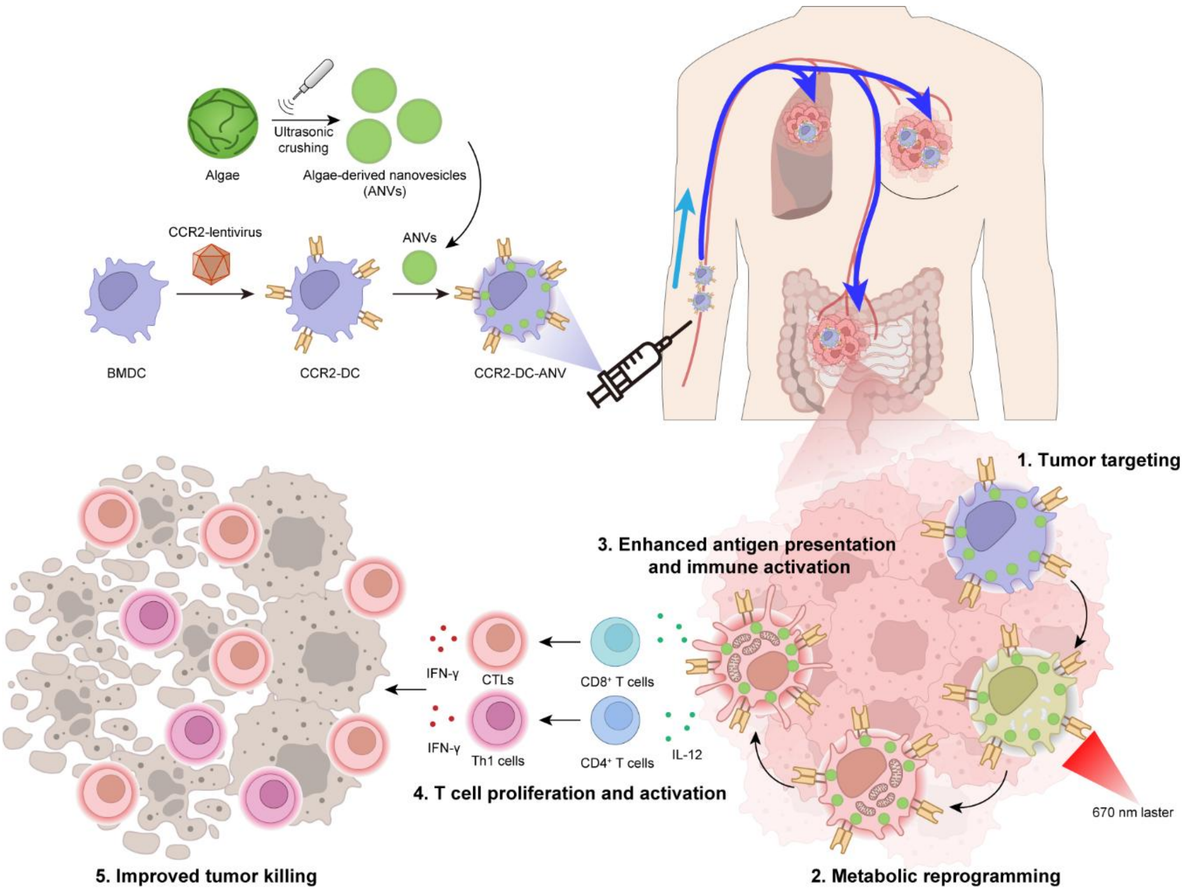

Supplementary Figure 1. Scheme of photosynthetic-boosted DC therapy with enhanced immune activity for cancer immunotherapy. First, algae-derived nanovesicles (ANVs) were obtained and incubated with CCR2^{hi} DCs to construct CCR2-DC-ANVs. By overexpressing CCR2 via lentivirus transfection, CCR2-DC-ANVs could infiltrate tumour following the systemic administration and ANVs would generate NADPH and oxygen upon red light irradiation, thus restoring mitochondrial morphology and function and relieving ER stress to reprogram cell metabolism in TME. CCR2-DC-ANVs performed enhanced immune activation and promoted the production of effector T cells, leading to killing effect against solid tumours.

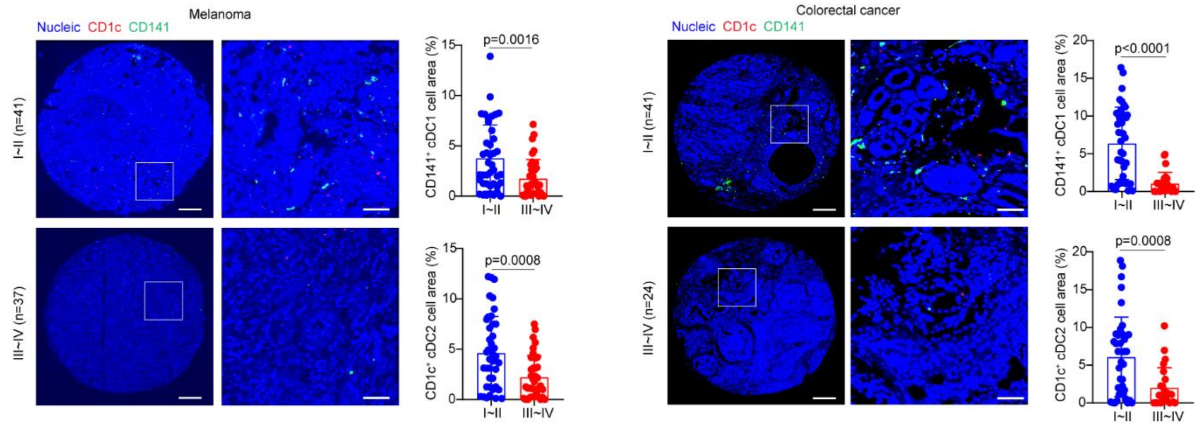

Supplementary Figure 2. Immunofluorescence staining of CD1c (cDC2s) and CD141 (cDC1s) in TMA of early (I-II)- or late (III-IV)-stage melanoma and colorectal cancer patients and quantification of CD1c and CD141 positive area out of the total tumour area (%). $n = 41$ (early) and $n = 37$ (late) patients for melanoma and $n = 41$ (early) and $n = 24$ (late) patients for colorectal cancer. Scale bars, 200 µm (left) and 50 µm (right). Data are mean ± s.d. Statistical analysis was evaluated with student's two-tailed unpaired *t*-test.

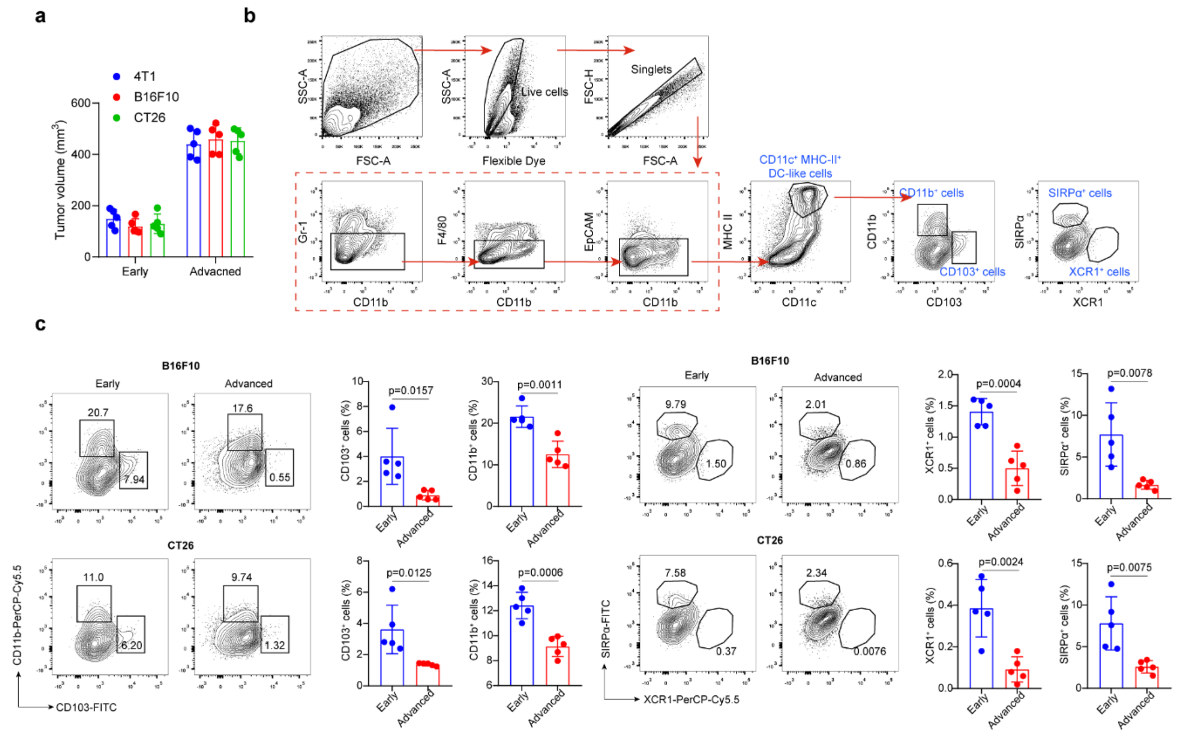

Supplementary Figure 3. a-b, Quantifications (**a**) of tumour volume at day 7 (early) and 12 (late) and flow cytometric gating strategies (**b**) correspond to Figure 1b-c and Supplementary Figure 3c to analyze CD11c^{+}MHC-II^{+} cells in tumours ($n = 5$). **c**, Representative flow cytometry plots and quantification of CD103^{+} and CD11b^{+} cells or XCR1^{+} and SIRPα^{+} cells (gated on CD11c^{+}MHC II^{+} cells) in B16F10 or CT26 tumour-bearing mice (n=5). Data are mean ± s.d. Statistical analysis was evaluated with student's two-tailed unpaired *t*-test.

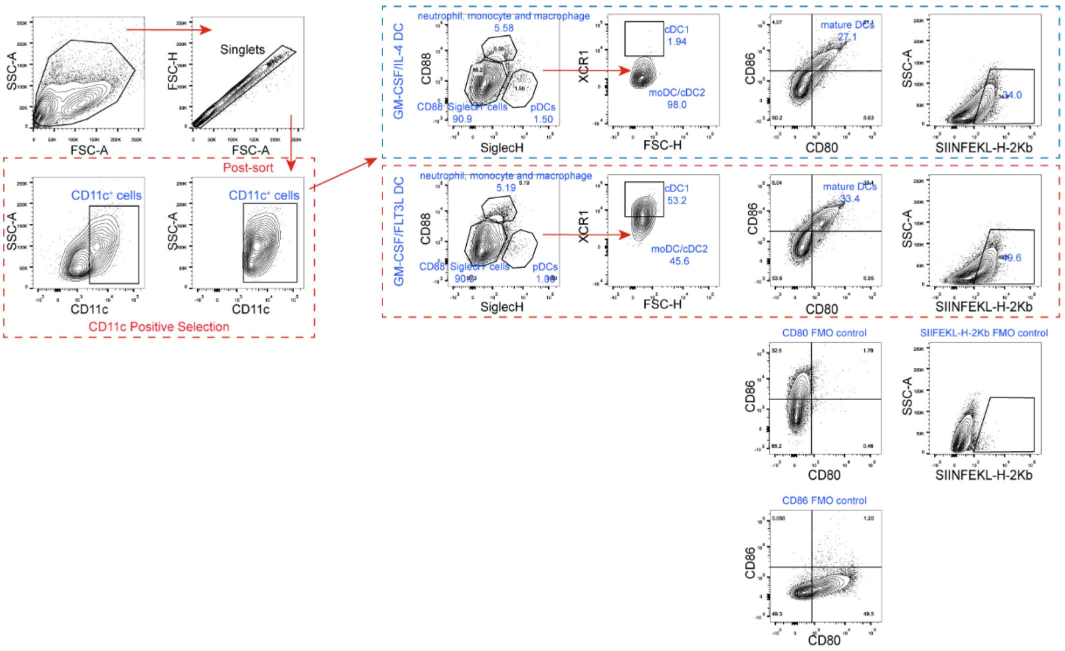

Supplementary Figure 4. Flow cytometric gating strategies correspond to Supplementary Figure 5-6 to analyze GM-CSF/IL-4- and GM-CSF/FLT3L-induced BMDCs. First, the generated BMDCs were sorted using CD11c positive selection kit. Then, the purified CD11c^{+} cells were labeled with different antibodies for further gating analysis.

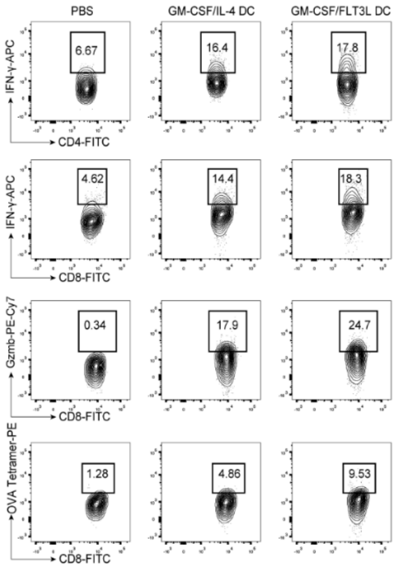

Supplementary Figure 5. Representative flow cytometry plots correspond to Supplementary Figure 6 of IFN-γ^{+} CD4^{+} T cells, IFN-γ^{+} CD8^{+} T cells, Gzmb^{+} CD8^{+} T cells and OVA-specific CD8^{+} T cells ($n = 5$).

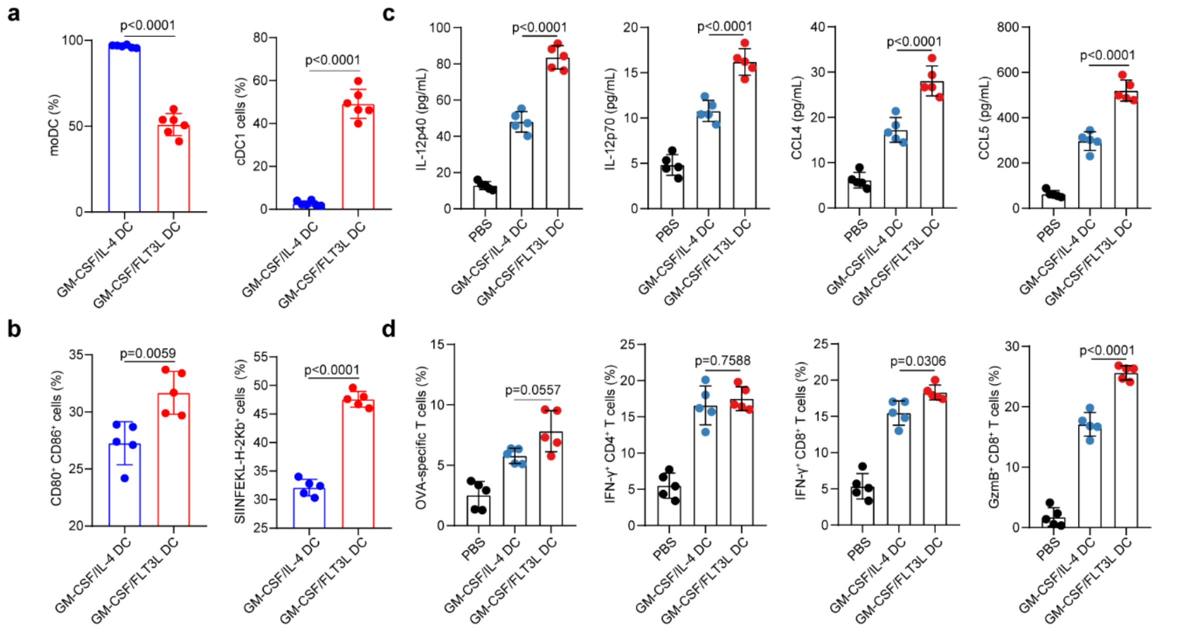

Supplementary Figure 6. Percentage of cDC1s and moDCs in GM-CSF/IL-4- and GM-CSF/FLT3L-induced BMDCs (**a**) and quantification of co-stimulatory molecule expression and antigen presentation of GM-CSF/IL-4- and GM-CSF/FLT3L-induced BMDCs (**b**) (n=5). **c**, Quantification of IL-12p40, IL-12p70, CCL4 and CCL5 secretion (n=5). **d**, Quantification of IFN-γ^+ $CD4^+$ T cells, IFN-γ^+ $CD8^+$ T cells, $Gzmb^+$ $CD8^+$ T cells and OVA-specific $CD8^+$ T cells (n=5). Data are mean ± s. d. Statistical analysis was evaluated with one-way ANOVA.

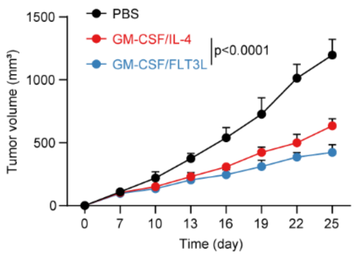

Supplementary Figure 7. 4T1-OVA tumour growth curves after treating with GM-CSF/IL-4- and GM-CSF/FLT3L-induced BMDCs (n=6). Data are mean ± s. d. Statistical analysis was evaluated with two-way ANOVA.

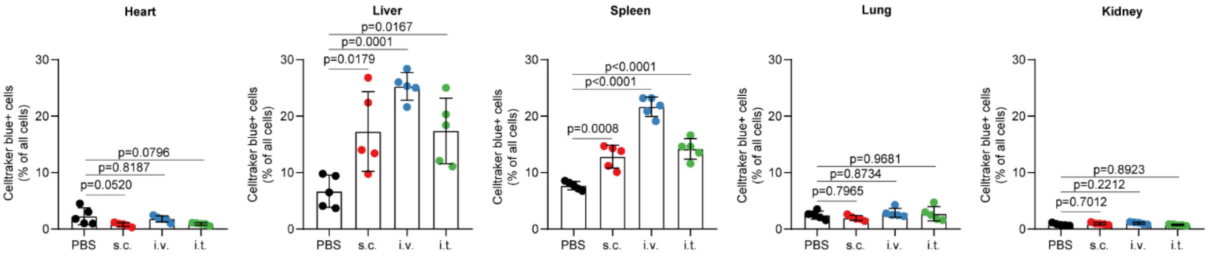

Supplementary Figure 8. Quantitative analysis of Celltracker $blue^+$ cells in major organs ($n = 5$). Data are mean ± s. d. Statistical analysis was evaluated with one-way ANOVA.

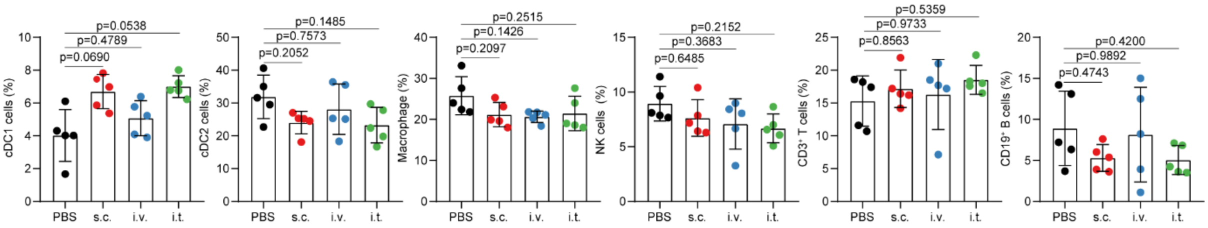

Supplementary Figure 9. Quantitative analysis of B cells, cDCs, macrophages, NK cells and T cells in tumours after adaptive transferring BMDCs through different routines ($n = 5$). Data are mean ± s. d. Statistical analysis was evaluated with one-way ANOVA.

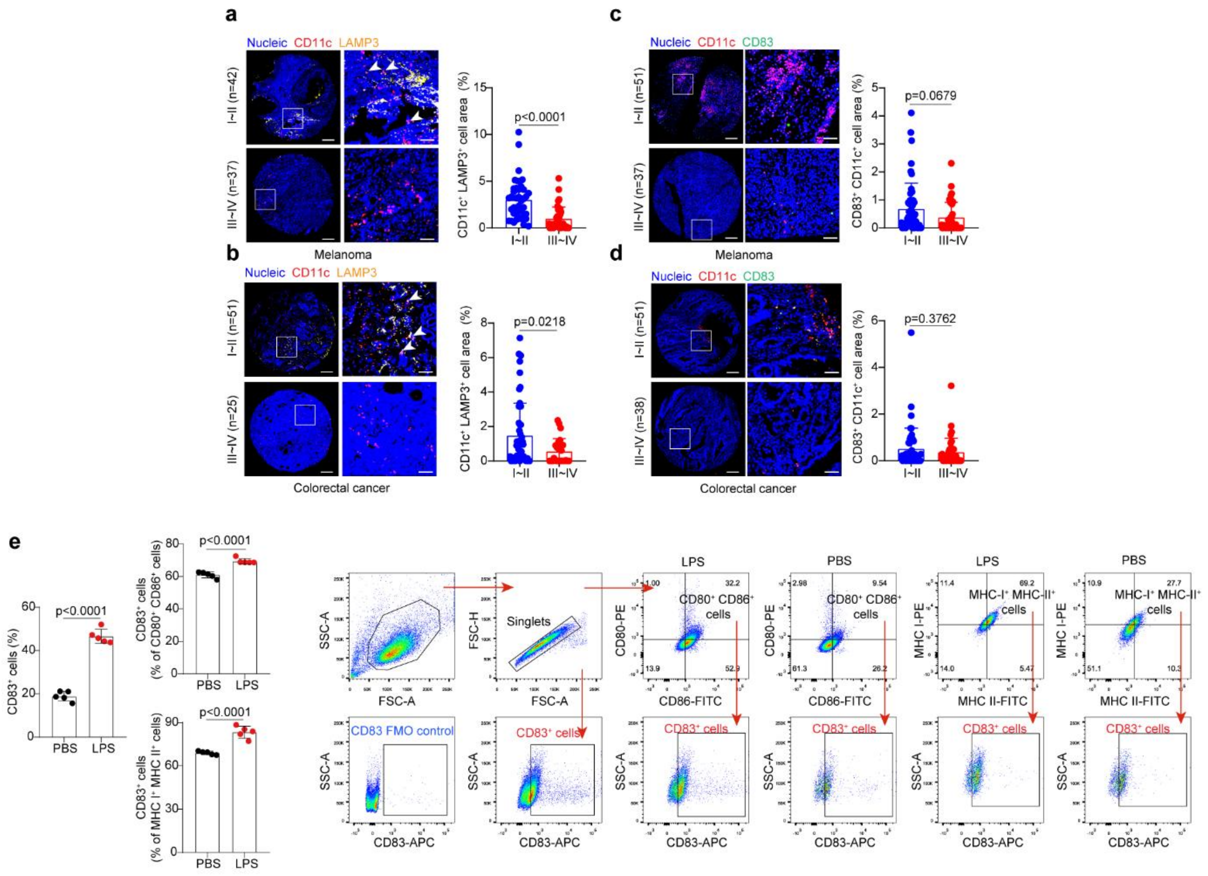

Supplementary Figure 10. a-b, Immunofluorescence staining of mature cDCs (CD11c and LAMP3) in melanoma (**a**) and colorectal cancer (**b**) patients and quantification of the $CD11c^{+}LAMP3^{+}$ double-positive area out of the total tumour area (%). $n = 42$ (early) and $n = 37$ (late) patients for melanoma and $n = 51$ (early) and $n = 25$ (late) patients for colorectal cancer. Scale bars, 200 μm (left) and 50 μm (right). **c-d,** Immunofluorescence staining of activated DCs (CD11c and CD83) in melanoma (**c**) and colorectal cancer (**d**) patients and quantification of the $CD11c^{+}CD83^{+}$ double-positive area out of the total tumour area (%). $n = 51$ (early) and $n = 37$ (late) patients for melanoma and $n = 51$ (early) and $n = 36$ (late) patients for colorectal cancer. Scale bars, 200 μm (left) and 50 μm (right). **e,** Quantitative analysis of CD83 expression

and flow cytometric gating strategies correspond to Supplementary Figure 10 to analyze CD83 expression in matured DCs (n=5). Data are mean ± s. d. Statistical analysis was evaluated with student's two-tailed unpaired *t*-test.

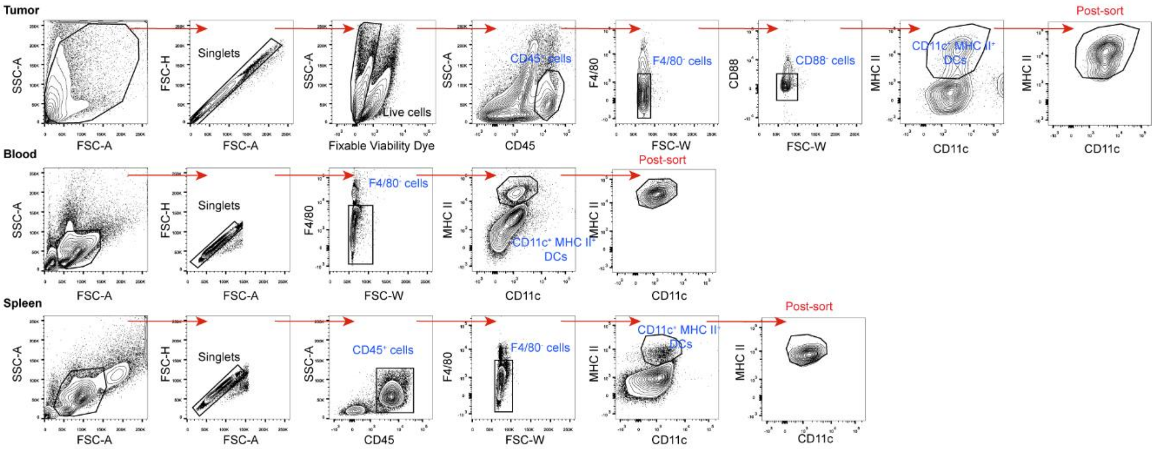

Supplementary Figure 11. Flow cytometric gating strategies correspond to Supplementary Figure 12 to sort DCs from tumour tissues, blood and spleen.

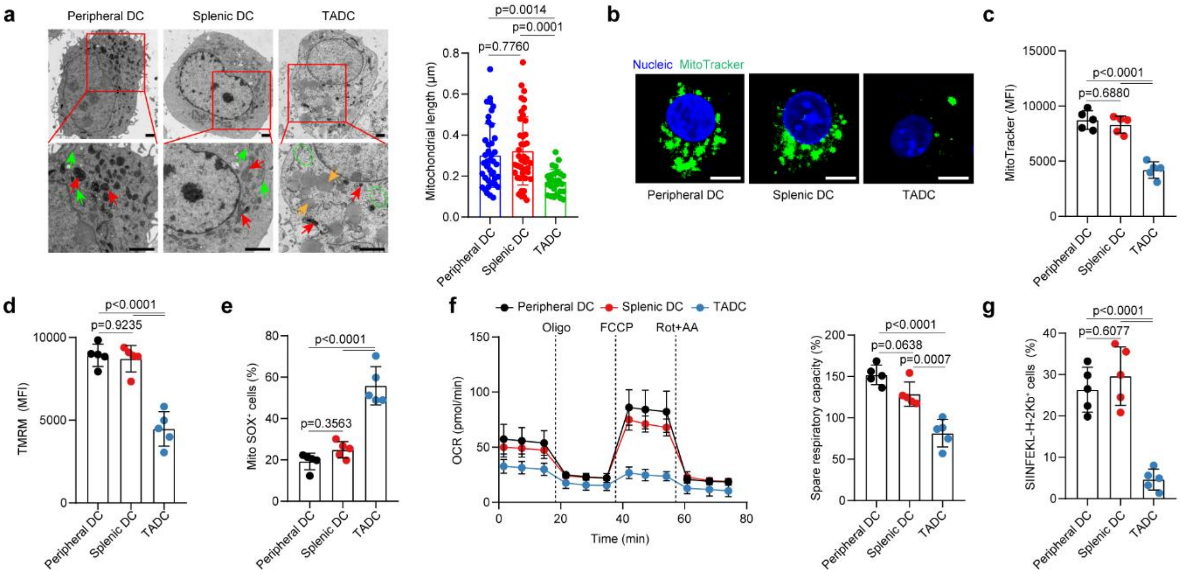

Supplementary Figure 12. Mitochondrial fragmentation and dysfunction in tumour-associated DCs. **a**, Transmission electron microscopy images showing the mitochondrial morphology of peripheral DCs ($n = 3$), Splenic DCs ($n = 3$) and TADCs ($n = 3$). The mitochondria were labeled by red arrows. The green and yellow arrows refer to ER and lipid droplets, respectively. Scale bar, 1 μm. Mitochondrial lengths were calculated in each group. **b-c**, Representative immunofluorescence images (**b**) and quantification (**c**) of MitoTracker in peripheral DCs and TADCs ($n = 3$). Scale bar, 10 μm. **d-e**, Quantitative analysis of mitochondrial membrane potential (labeled by TMRM) (**d**) and MitoSOX (**e**) in peripheral DCs, splenic DCs

and TADCs (n=5). **f**, Oxygen consumption rate (OCR) analysis of DCs and the calculated spare respiratory capacity (n=5). **g**, Quantitative analysis of SIINFEKL-H-2Kb^{+} cells in peripheral DCs, splenic DCs and TADCs (n=5). Data are mean ± s. d. Statistical analysis was evaluated with one-way ANOVA.

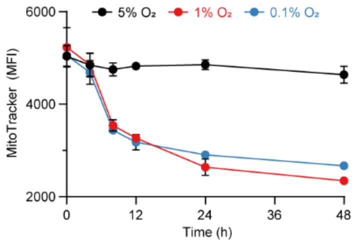

Supplementary Figure 13. Quantitative analysis of MitoTracker at different time points when DCs were cultured under different oxygen tensions (n=3).

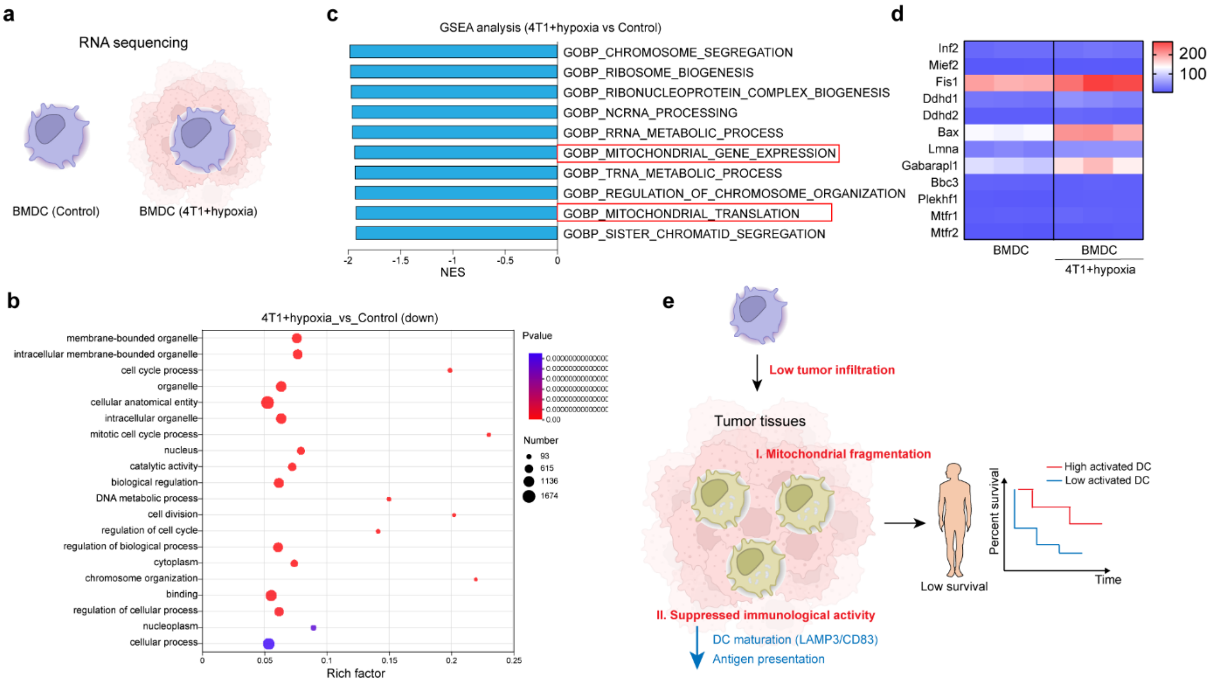

Supplementary Figure 14. a, Scheme of BMDCs for RNA sequencing. **b**, GO enrichment analysis of downregulated genes between control and BMDC (4T1 incubation+0.1% O_2) groups ($n = 3$). **c**, GSEA enrichment analysis of biological processes between control and BMDC (4T1 incubation+0.1% O_2) groups ($n = 3$). **d**, Heatmap showing expression of mitochondrial fission-related genes in control and BMDC (4T1 incubation+0.1% O_2) groups (n=3). **e**, Scheme of the relationship of DC infiltration and activity to patient prognosis and survival.

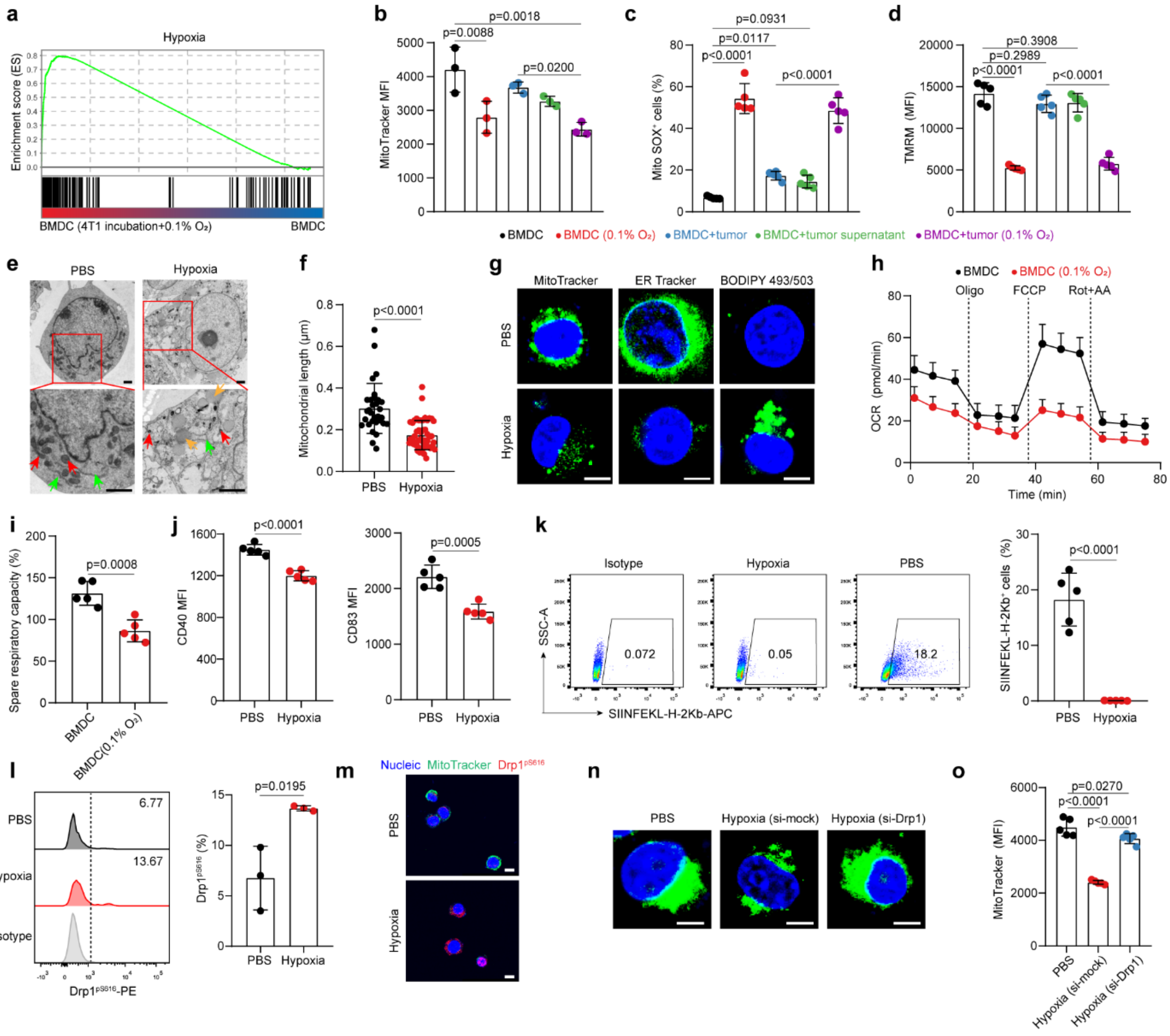

Supplementary Figure 15. Hypoxia induces mitochondrial fragmentation in DCs. a, GSEA showing the enrichment of PI3K-Akt-mTOR signaling in CCR2-DC-ANV+L (4T1 incubation + 0.1% O_2) group compared with BMDC (4T1 incubation + 0.1% O_2) group ($n = 3$). **b-d**, Quantitative analysis of MitoTracker (**b**) (n = 3), MitoSOX (**c**) (n = 5) and mitochondrial membrane potential (labeled by TMRM) (**d**) (n = 5) of BMDCs after incubated with tumour cells or in hypoxia. **e-f**, Transmission electron microscopy images (**e**) showing the mitochondrial morphology of BMDCs and hypoxic BMDCs ($n = 3$). The mitochondria were labeled by red arrows. The green and yellow arrows refer to ER and lipid droplets, respectively. Scale bar, 1 μm. Mitochondrial lengths in Supplementary Figure 15e (**f**) were calculated from one cell in each group. **g**, Representative immunofluorescence images of MitoTracker, ER Tracker and BODIPY493/503 in BMDCs and hypoxic BMDCs ($n = 3$). Scale bar, 10 μm. **h-i**, Oxygen consumption rate (OCR) (**h**) analysis of BMDCs and hypoxic BMDCs and the calculated spare respiratory capacity (**i**) (n=5). **j**, Quantitative analysis of CD40 and CD83 of BMDCs and hypoxic BMDCs (n=5). **k**, Representative flow cytometry plots and quantification

of SIINFEKL-H-2Kb^{+} cells in BMDCs and hypoxic BMDCs (n=5). **l**, Representative flow cytometry plots and quantification of Drp1 phosphorylated at Ser616 (Drp1^{pS616}) in BMDCs and hypoxic BMDCs (n=5). **m**, Representative immunofluorescence images of MitoTracker and Drp1^{pS616} in BMDCs and hypoxic BMDCs (n = 3). **n-o**, Representative immunofluorescence images (**n**) and quantification (**o**) of MitoTracker in BMDCs and hypoxic BMDCs after knocking down Drp1 expression (si-Drp1) (n = 3). Data are mean ± s. d. Statistical analysis was evaluated with student's two-tailed unpaired t-test (**f**, **i**, **j**, **k, l**) and one-way ANOVA (**b**, **c**, **d**, **o**).

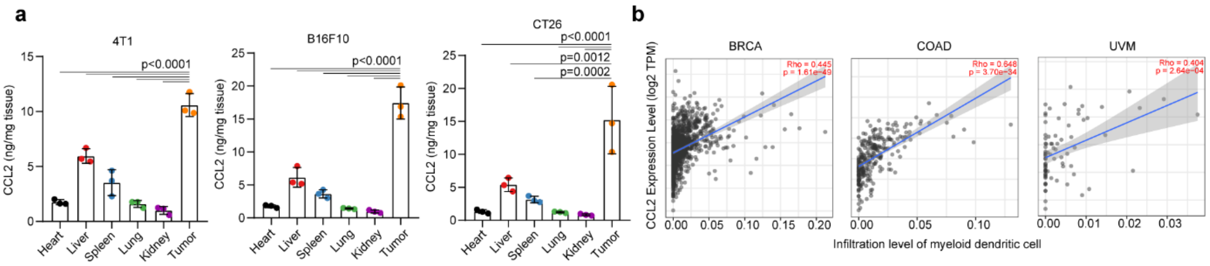

Supplementary Figure 16. a, CCL2 level in tumours and different organs in 4T1-, B16F10- and CT26-bearing mice (n=3). **b**, Kaplan–Meier curve of the linear correlation of CCL2 and DC infiltration calculated by TIMER bioinformatic platform of TCGA databases. BRCA (n=1100), COAD (n=458) and UVM (n=80). Data are mean ± s. d. Statistical analysis was evaluated with one-way ANOVA.

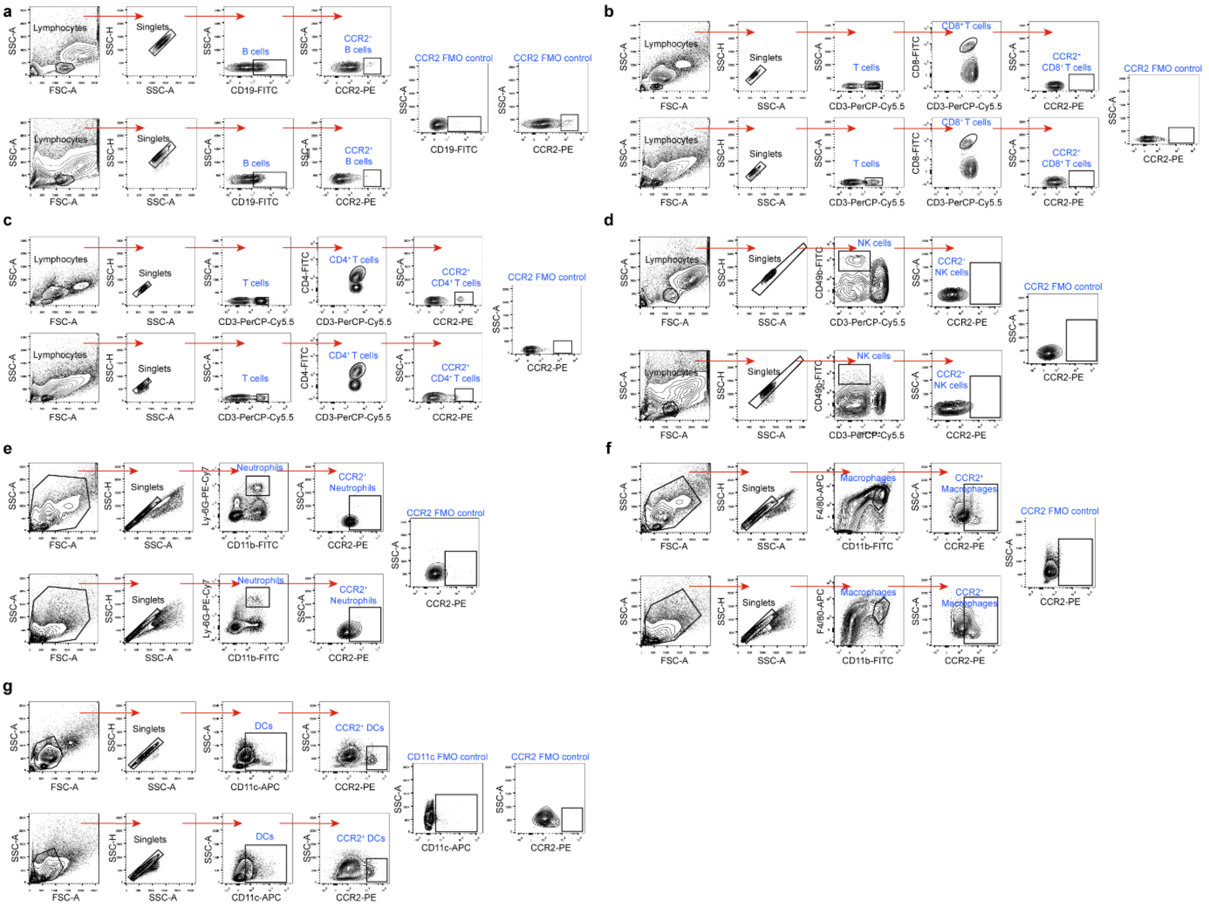

Supplementary Figure 17. Flow cytometric gating strategies correspond to Supplementary Figure 18 to analyze CCR2$^+$ B cells (**a**), CCR2$^+$ CD8$^+$ T cells (**b**), CCR2$^+$ CD4$^+$ T cells (**c**), CCR2$^+$ NK cells (**d**), CCR2$^+$ neutrophils (**e**) CCR2$^+$ macrophages (**f**) and CCR2$^+$ DCs (**g**) in blood (up) and spleen (down).

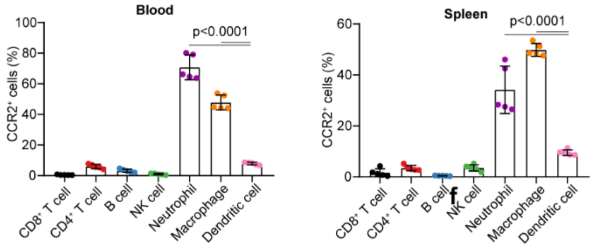

Supplementary Figure 18. CCR2 expression of different immune cells in blood and spleen (n=5). Data are mean ± s. d. Statistical analysis was evaluated with one-way ANOVA.

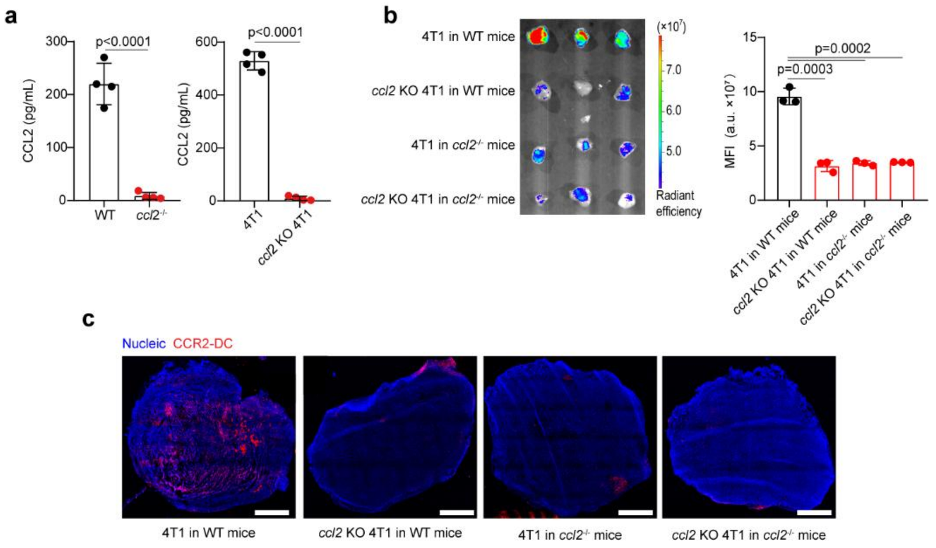

Supplementary Figure 19. **a**, Quantitative analysis of blood CCL2 level in WT and *ccl2*$^{-/-}$ mice (left) and supernatant CCL2 level in 4T1 and *ccl2* knockout 4T1 cells ($n = 4$). **b,** Representative fluorescence images and quantitative analysis of tumours in tumour-bearing mice after i.v. administration of CCR2-DCs (CCR2-DCs were labeled with DiI) (n=3). **c**, Representative immunofluorescence images of CCR2-DCs in tumours ($n = 3$). Scale bar, 1 mm. Data are mean ± s. d. Statistical analysis was evaluated with student's two-tailed unpaired *t*-test (**a**) and one-way ANOVA (**b**).

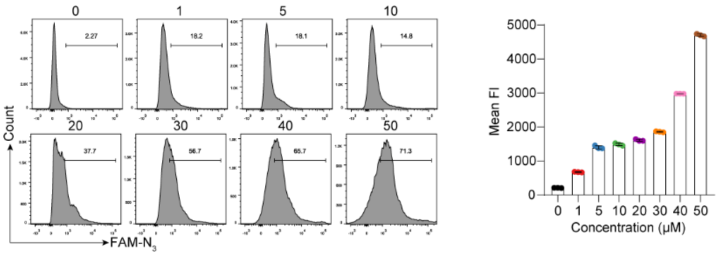

Supplementary Figure 20. Representative cytometry histograms and quantitative analysis of DBCO conjugation on algae (n=3). 1×10^7 algae cells were treated with different concentration (0, 1, 5, 10, 20, 30, 40, 50 μM) of DBCO-PEG$_{2000}$-NHS ester for 45 min at room temperature RT.

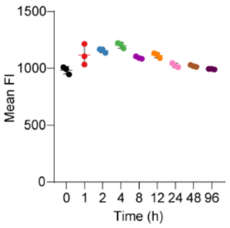

Supplementary Figure 21. Quantitative analysis of DBCO modification on algae at different time points post-incubation (n=3).

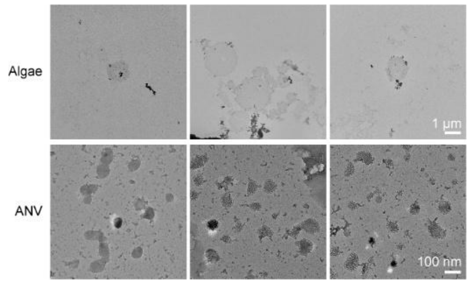

Supplementary Figure 22. Representative transmission electron microscopy images of algae and ANVs ($n = 3$). Scar bar, 1 μm (up) and 100 nm (down).

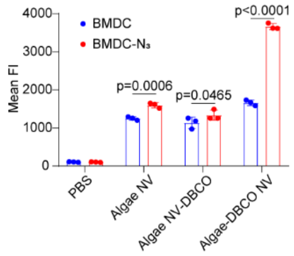

Supplementary Figure 23. Quantitative analysis of ANV conjugation on BMDCs (n=3). Data are mean ± s. d. Statistical analysis was evaluated with two-way ANOVA.

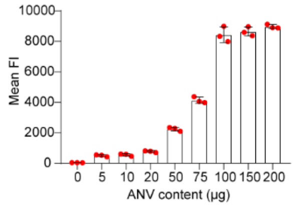

Supplementary Figure 24. Quantitative analysis of appropriate ANV concentration when incubated with BMDCs (n=3).

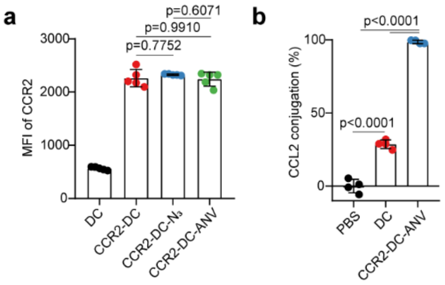

Supplementary Figure 25. Quantitative analysis of CCR2 expression on CCR2-DC-ANVs ($n = 5$) and the CCL2-CCR2 interaction of CCR2-DC-ANVs using ELISA analysis ($n = 4$). Data are mean ± s. d. Statistical analysis was evaluated with one-way ANOVA.

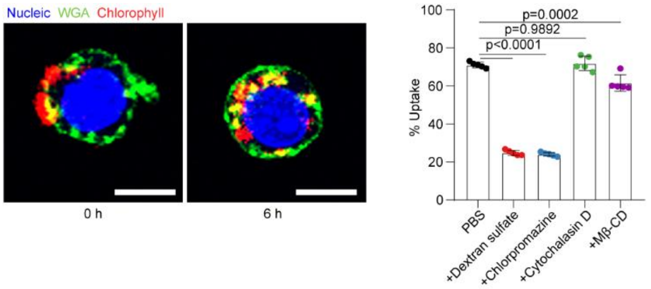

Supplementary Figure 26. Representative immunofluorescence images of CCR2-DC-ANVs. ANVs were labeled by chlorophyll (red) and the cell membrane was labeled by WGA (green). Scale bar, 10 μm. Uptake of ANVs by BMDCs after endocytosis inhibitor treatment (n=5). In particular, dextran sulfate, chlorpromazine, cytochalasin D Mβ-CD are inhibitors of scavenger receptor class A-mediated endocytosis, clathrin-mediated endocytosis, micropinocytosis/phagocytosis and lipid rafts/cholesterol-enriched microdomains/caveolae. Data are mean ± s. d. Statistical analysis was evaluated with one-way ANOVA.

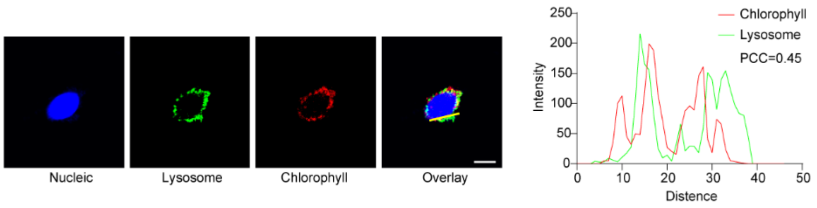

Supplementary Figure 27, Fluorescent visualization of ANVs localization in BMDCs 6 h after incubation (ANVs, red; nuclei, blue; lysosome, green) and intensity profiles across the cell along the selected line (yellow line) ($n = 3$). Scale bar, 5 μm.

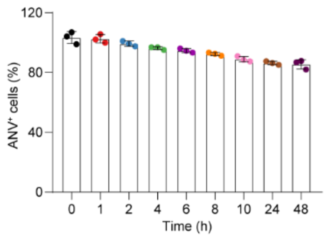

Supplementary Figure 28. Quantitative analysis of ANVs in BMDCs post-incubation at different times (n=3).

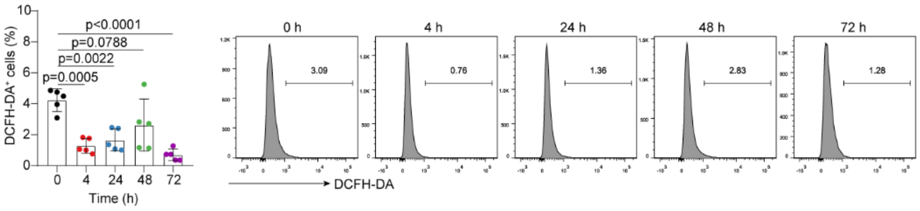

Supplementary Figure 29. Quantitative analysis and representative cytometry histograms of intracellular reactive oxygen species (ROS) in CCR2-DC-ANVs (n=5). Data are mean ± s. d. Statistical analysis was

evaluated with one-way ANOVA.

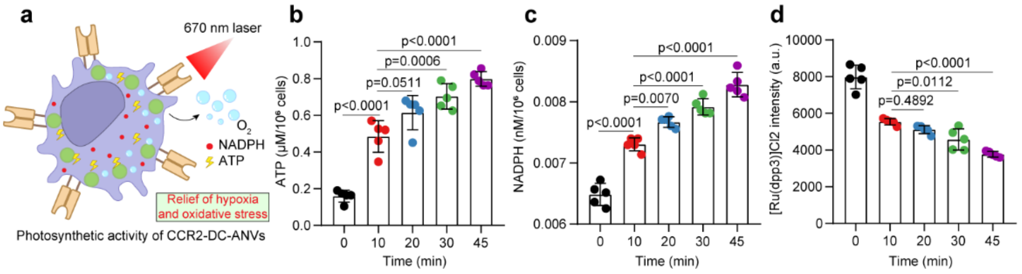

Supplementary Figure 30. a, Scheme illustration of ATP, NADPH and oxygen generation of CCR2-DC-ANVs. **b-d**, Quantitative analysis of intracellular ATP (**b**), NADPH (**c**) and [Ru(dpp3)] Cl2 intensity (**d**) of CCR2-DC-ANVs under red light irradiation for different times (n=5). Data are mean ± s. d. Statistical analysis was evaluated with one-way ANOVA.

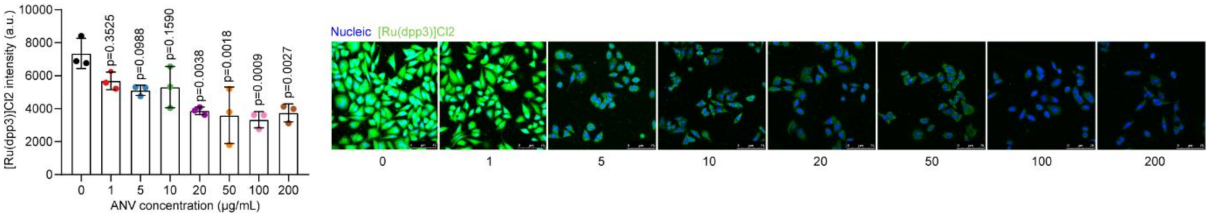

Supplementary Figure 31. [Ru(dpp3)] Cl2 intensity and representative immunofluorescence images of [Ru(dpp3)] Cl2 in CCR2-DC-ANVs after incubation with different content of ANVs plus red-light exposure ($n = 3$). Scale bar, 75 μm. Data are mean ± s. d. Statistical analysis was evaluated with one-way ANOVA.

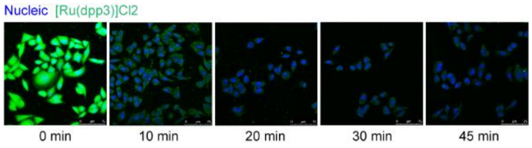

Supplementary Figure 32. Representative immunofluorescence images of [Ru(dpp3)] Cl2 in CCR2-DC-ANVs after irradiation of different durations ($n = 3$). Scale bar, 75 μm.

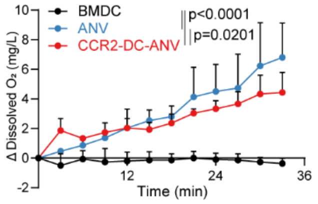

Supplementary Figure 33. Dissolved oxygen content of the BMDCs, ANVs and CCR2-DC-ANVs under red light irradiation (n=3). Data are mean ± s. d. Statistical analysis was evaluated with two-way ANOVA.

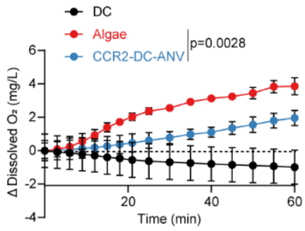

Supplementary Figure 34. Dissolved oxygen content of the BMDCs, algae and CCR2-DC-ANVs under red light irradiation (*n* = 3). Data are mean ± s. d. Statistical analysis was evaluated with two-way ANOVA.

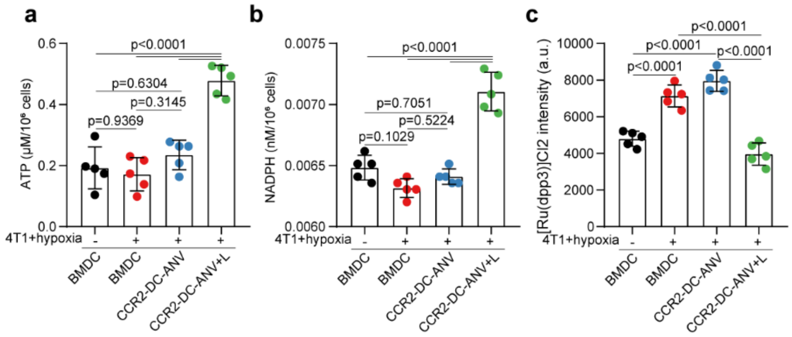

Supplementary Figure 35. Quantitative analysis of intracellular ATP (**a**), NADPH (**b**) and [Ru(dpp3)] Cl2 intensity (**c**) of irradiated CCR2-DC-ANVs incubated with tumour cells under hypoxia condition (n=5). Data are mean ± s. d. Statistical analysis was evaluated with one-way ANOVA.

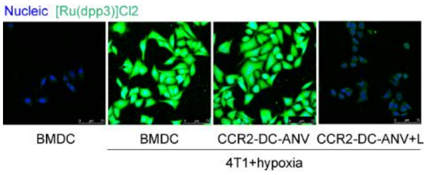

Supplementary Figure 36. Representative immunofluorescence images of [Ru(dpp3)] Cl2 in different groups (*n* = 3). Scale bar, 75 μm.

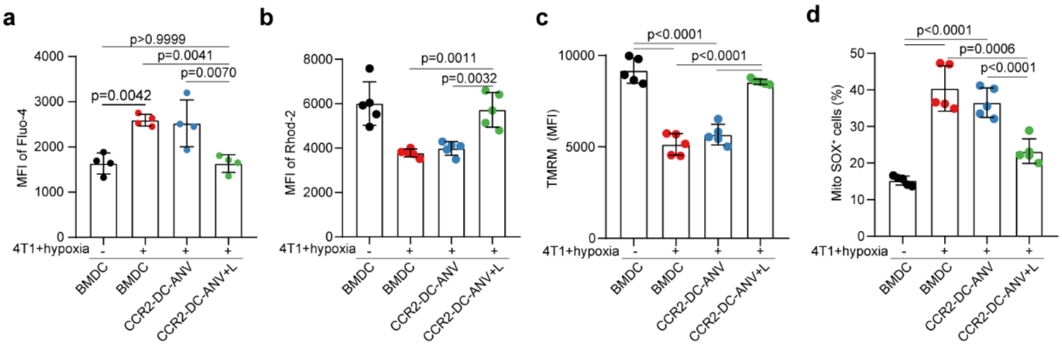

Supplementary Figure 37. a-b, Quantitative analysis of cytosolic calcium (**a**) and mitochondrial calcium (**b**) in CCR2-DC-ANVs (n=5). **c-d**, Quantitative analysis of mitochondrial membrane potential (labeled by TMRM) (**c**) and MitoSOX (**d**) in different groups (n=5). Data are mean ± s. d. Statistical analysis was

evaluated with one-way ANOVA.

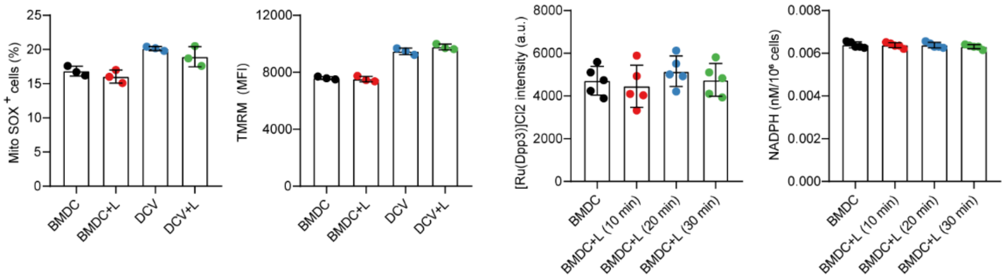

Supplementary Figure 38. Quantitative analysis of MitoSOX and TMRM in BMDCs or DC vaccines (DCVs) upon light exposure (n=3). Quantitative analysis of [Ru(dpp3)] Cl2 intensity and NADPH level in BMDCs upon light exposure (n=5). Data are mean ± s. d. Statistical analysis was evaluated with one-way ANOVA.

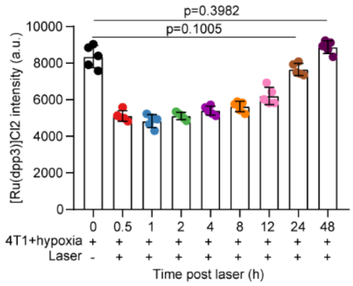

Supplementary Figure 39. Quantitative analysis of [Ru(dpp3)] Cl2 intensity in CCR2-DC-ANVs at different time points post-irradiation (n=5). Data are mean ± s. d. Statistical analysis was evaluated with one-way ANOVA.

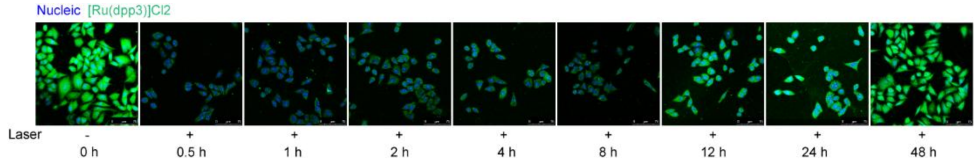

Supplementary Figure 40. Representative immunofluorescence images of [Ru(dpp3)] Cl2 in CCR2-DC-ANVs post-irradiation for different times ($n = 3$). Scale bar, 75 µm.

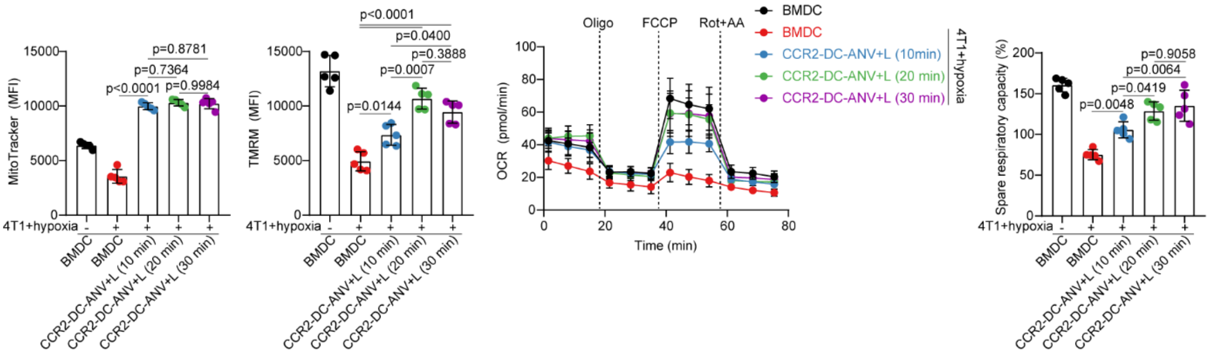

Supplementary Figure 41. Quantitative analysis of MitoTracker and TMRM in CCR2-DC-ANVs after irradiation of different durations (n=5). Oxygen consumption rate (OCR) analysis of CCR2-DC-ANVs after

irradiation of different durations and the calculated spare respiratory capacity (n=5). Data are mean ± s. d. Statistical analysis was evaluated with one-way ANOVA.

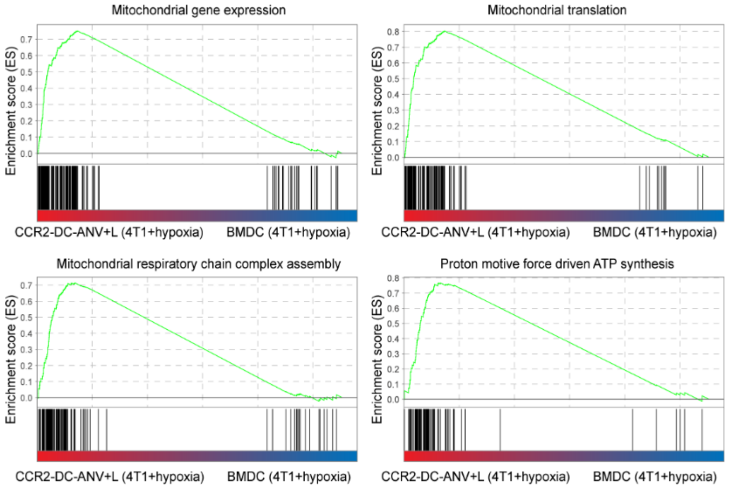

Supplementary Figure 42. GSEA enrichment analysis of the differentially expressed genes in BMDC (4T1+hypoxia) and CCR2-DC-ANV group after red light irradiation ($n = 3$).

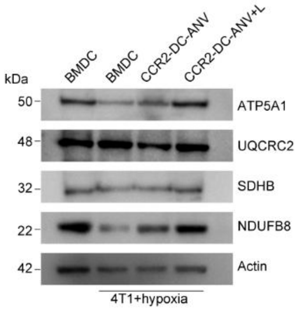

Supplementary Figure 43. Western blotting analysis of mitochondrial function-related protein expression in different groups ($n = 3$).

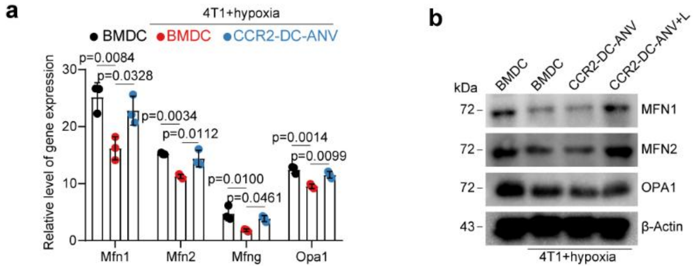

Supplementary Figure 44. a, Relative levels of mitochondrial fusion-related genes in different groups via transcriptomic analysis (n = 3). **b**, Western blotting analysis of MFN1/2 and OPA1 in different groups ($n = 3$). Data are mean ± s. d. Statistical analysis was evaluated with one-way ANOVA.

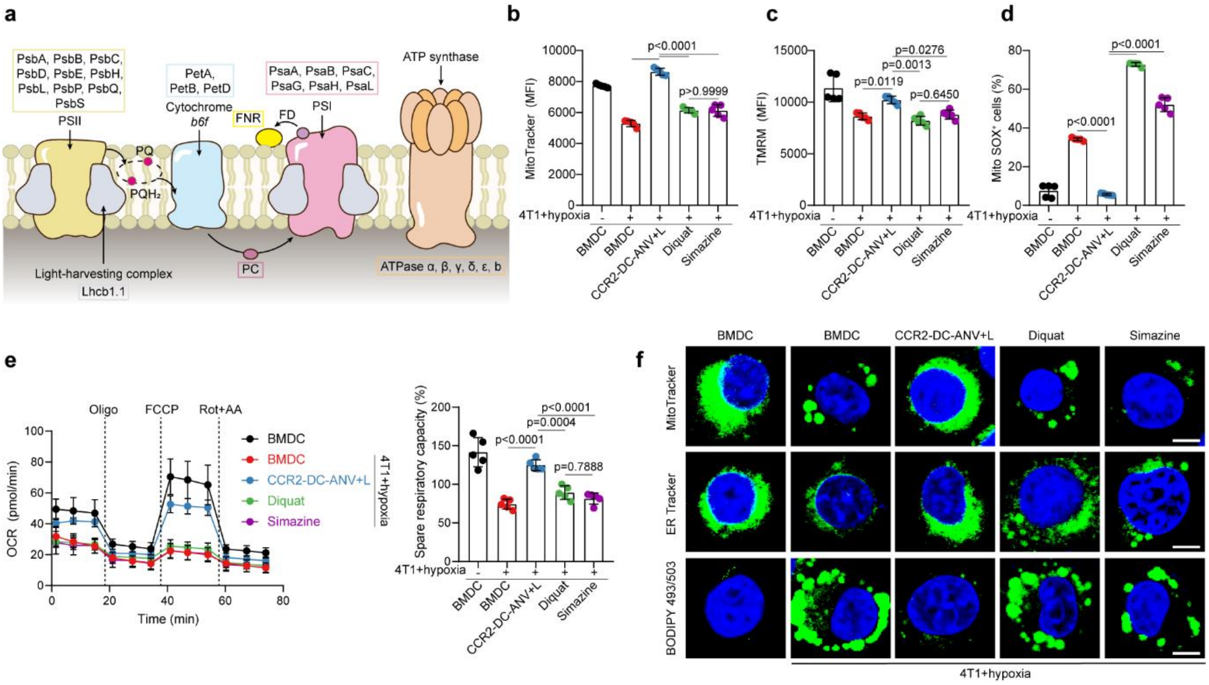

Supplementary Figure 45. a, Schematic illustration of photosynthesis light reaction-associated proteins and the photosynthetic electron transport chain in algae and ANVs. FD, ferredoxin; PC, plastocyanin; PSI, photosystem I; PSII, photosystem II; PQ, plastoquinone. **b-d**, Quantitative analysis of MitoTracker (**b**) mitochondrial membrane potential (labeled by TMRM) (**c**) and MitoSOX (**d**) in different groups (n=5). **e**, Oxygen consumption rate (OCR) analysis of CCR2-DC-ANVs and the calculated spare respiratory capacity (n=5). **f**, Representative immunofluorescence images of MitoTracker, ER Tracker and BODIPY493/503 in different groups (*n* = 3). Scale bar, 5 μm. Data are mean ± s. d. Statistical analysis was evaluated with one-way ANOVA.

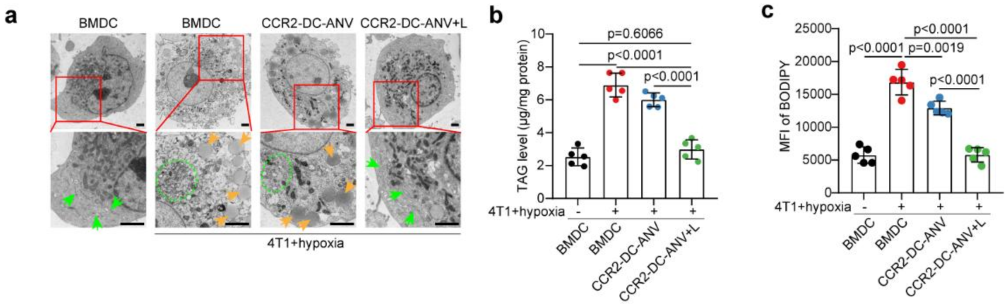

Supplementary Figure 46. a, Transmission electron microscopy images showing the ER stress and lipid droplet (LD) accumulation of DCs (*n* = 3). The ER and LD were labeled by green and yellow arrows, respectively. Scale bar, 1 μm. **b**, TAG levels in BMDCs and CCR2-DC-ANVs after red light exposure (n=5). **c**, Quantitative analysis of BODIY-stained lipids in different groups (n=5). Data are mean ± s. d. Statistical analysis was evaluated with one-way ANOVA.

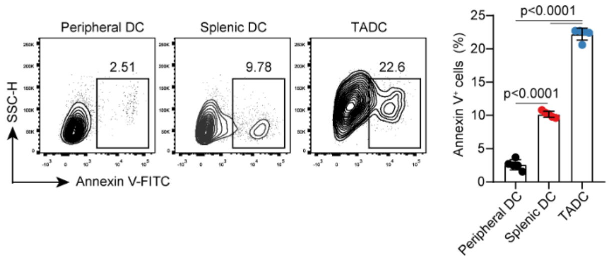

Supplementary Figure 47. Representative flow cytometry plots and quantitative analysis of apoptosis (annexin V^{+}) in peripheral DCs and TADCs (n=5). Data are mean ± s. d. Statistical analysis was evaluated with one-way ANOVA.

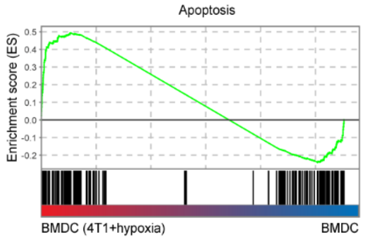

Supplementary Figure 48. GSEA enrichment analysis of Apoptosis between control and BMDC (4T1+hypoxia) groups ($n = 3$).

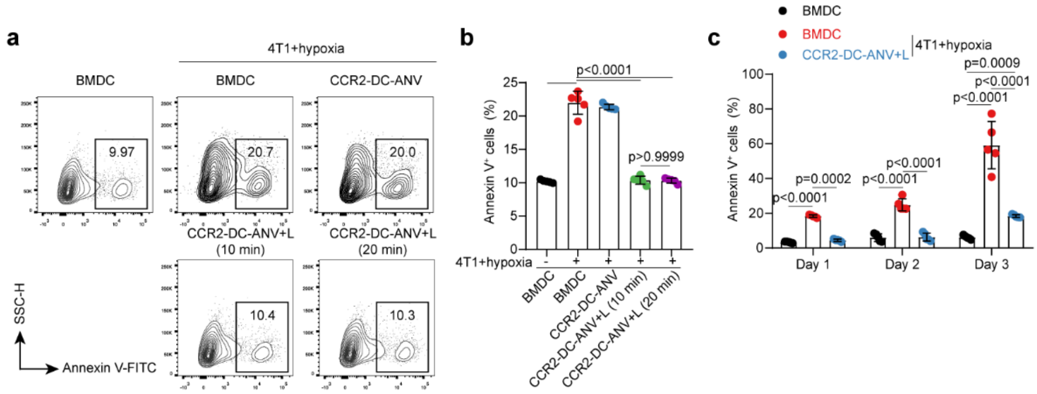

Supplementary Figure 49. a-b, Representative flow cytometry plots (**a**) and quantitative analysis (**b**) of apoptosis (annexin V^{+}) in BMDCs and CCR2-DC-ANVs (n=5). **c,** Quantitative analysis of apoptosis (annexin V^{+}) in BMDCs and CCR2-DC-ANVs on day 1, day 2 and day 3 (n=5). Data are mean ± s. d. Statistical analysis was evaluated with one-way ANOVA.

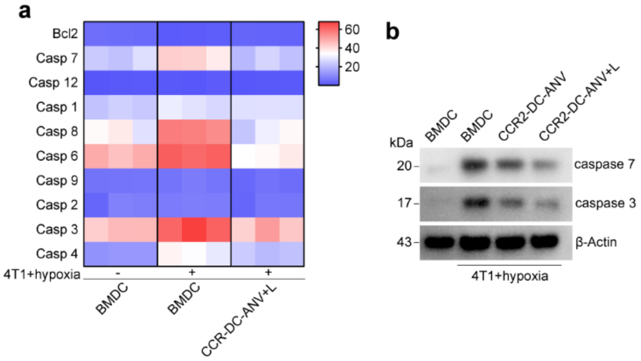

Supplementary Figure 50. a, Heatmap showing expression of apoptosis-related genes in BMDCs and CCR2-DC-ANVs (n=3). **b,** Western blotting analysis of caspase 3 and caspase 7 in different groups ($n = 3$).

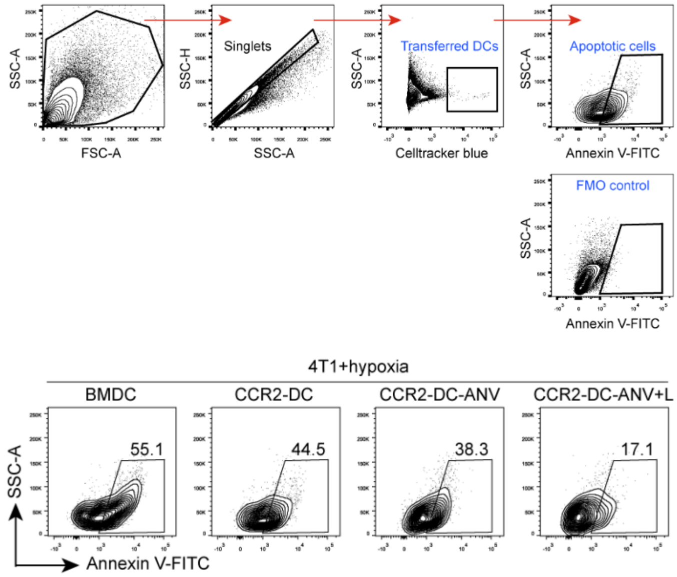

Supplementary Figure 51. Flow cytometric gating strategy and representative flow cytometry plots correspond to Supplementary Figure 52 for apoptotic transferred CCR2-DC-ANVs in tumours.

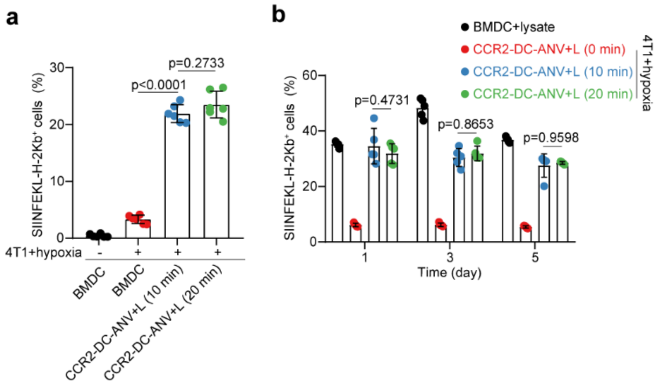

Supplementary Figure 52. a, Quantitative analysis of SIINFEKL-H-2Kb^{+} cells in CCR2-DC-ANVs irradiated for 10 and 20 min (n=5). **b**, Quantitative analysis of SIINFEKL-H-2Kb^{+} cells in CCR2-DC-ANVs several days after light exposure (n=5). Data are mean ± s. d. Statistical analysis was evaluated with one-

way ANOVA.

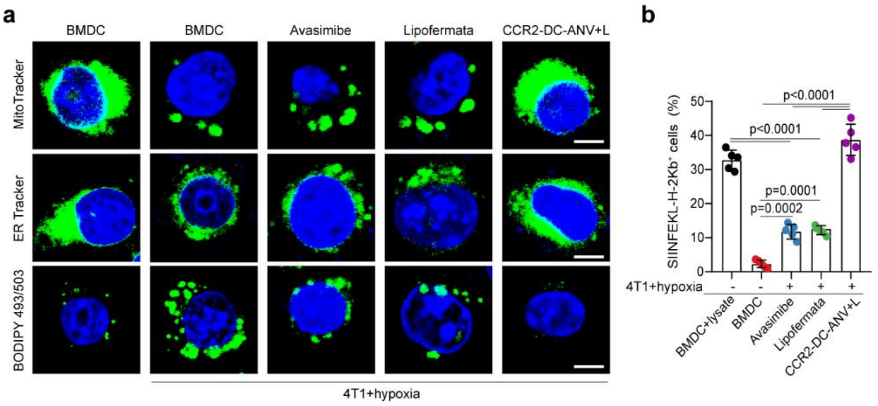

Supplementary Figure 53. Quantitative analysis of SIINFEKL-H-2Kb^{+} cells (**a**) and representative immunofluorescence images of MitoTracker, ER Tracker and BODIPY493/503 (**b**) in BMDCs and CCR2-DC-ANVs after avasimibe and lipofermata treatment (*n* = 5). Scale bar, 5 μm. Data are mean ± s. d. Statistical analysis was evaluated with one-way ANOVA.

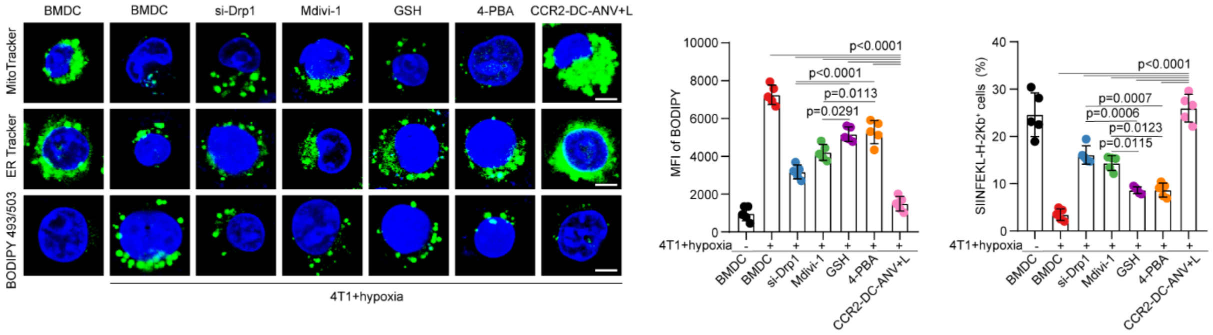

Supplementary Figure 54. Representative immunofluorescence images of MitoTracker, ER Tracker and BODIPY 493/503 in CCR2-DC-ANVs after different treatments. Scar bar, 5 μm. Quantitative analysis of BODIPY 493/503 and SIINFEKL-H-2Kb^{+} cells in CCR2-DC-ANVs after different treatments (n=5). Data are mean ± s. d. Statistical analysis was evaluated with one-way ANOVA.

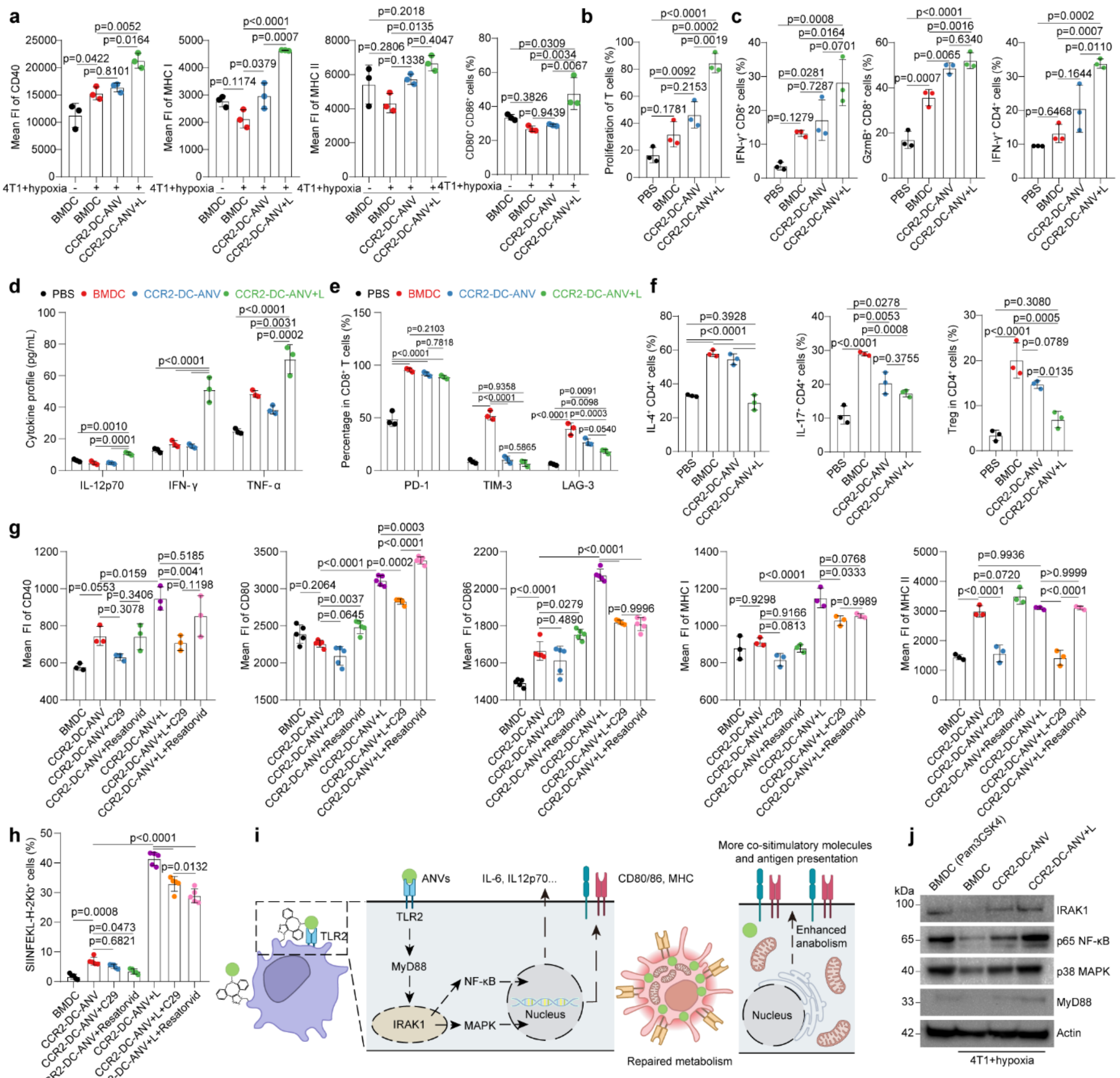

Supplementary Figure 55. CCR2-DC-ANVs enhance anticancer immune response in vitro. a, Quantitative analysis of CD40, MHC I and MHC II expression and $CD80^+$ $CD86^+$ cells among BMDCs and CCR2-DC-ANVs (n=3). **b**, Quantitative analysis of the proliferation rate of T cells (n=3). **c**, Quantitative analysis of IFN-γ^+ $CD8^+$ T cells, $GzmB^+$ $CD8^+$ T cells and IFN-γ^+ $CD4^+$ T cells after CCR2-DC-ANV incubation (n=3). **d**, Cytokine profiles in the cell culture medium measured by ELISA (n=3). **e**, Quantitative analysis of PD-1, TIM-3 and LAG-3 expression on $CD8^+$ T cells (n=3). **f**, Quantitative analysis of Th2 (IL-4^+ $CD4^+$ T cells), Th17 (IL-17^+ $CD4^+$ T cells) and Treg ($Foxp3^+$ $CD4^+$ T cells) after CCR2-DC-ANV incubation (n=3). **g**, Quantitative analysis of CD40, CD80, CD86, MHC I and MHC II expression among BMDCs and CCR2-DC-ANVs after toll-like receptor (TLR) inhibitor treatment (n=5). **h**, Quantitative analysis of SIINFEKL-H-$2Kb^+$ cells ($n = 5$). **i**, Scheme of the activation of TLR2 signaling pathway in

CCR2-DC-ANVs. The repaired mitochondrial function leads to enhanced anabolism in CCR2-DC-ANVs, thus further improve co-stimulatory molecule expression and antigen presentation. **j**, Western blotting analysis of TLR2 signaling pathway-related protein expression in different groups ($n = 3$). Data are mean ± s. d. Statistical analysis was evaluated with one-way ANOVA.

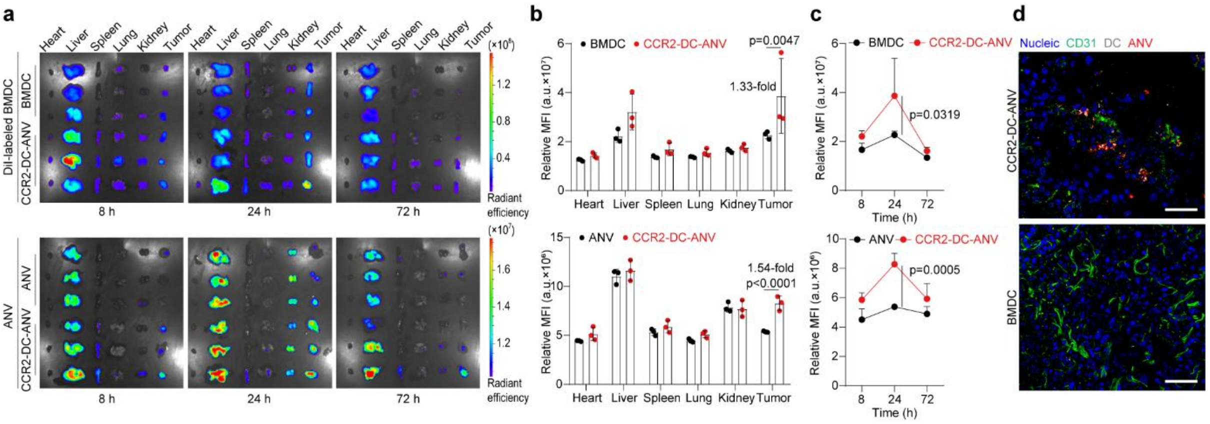

Supplementary Figure 56. a-b, Representative fluorescence images (**a**) and quantitative analysis (**b**) of major organs of tumour-bearing mice after i.v. administration of CCR2-DC-ANVs (DCs and ANVs were labeled with DiI and chlorophyll, respectively) (n=3). **c**, Quantitative fluorescence analysis of tumours at different time points after treatment ($n = 3$). **d**, Representative immunofluorescence images of BMDCs and CCR2-DC-ANVs in tumour ($n = 3$). Scale bar, 50 μm. Data are mean ± s. d. Statistical analysis was evaluated with one-way ANOVA.

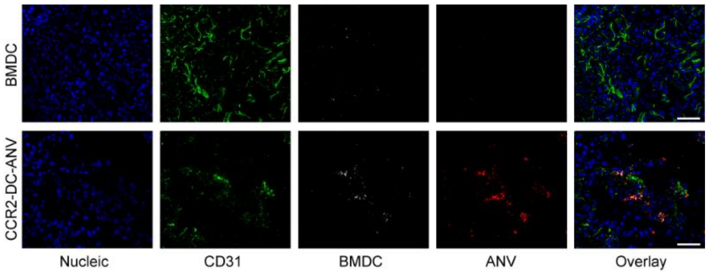

Supplementary Figure 57. Immunofluorescence images of BMDCs and CCR2-DC-ANVs in tumour ($n = 3$). Scale bar, 50 μm.

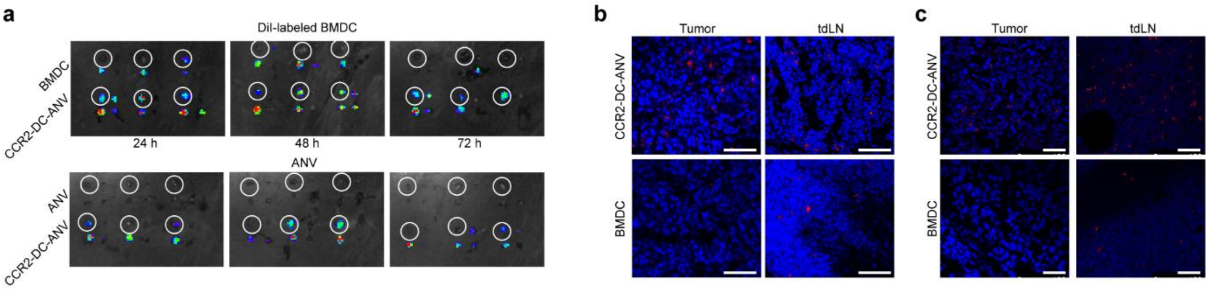

Supplementary Figure 58. a, Representative fluorescence images of tumour-derived lymph nodes (tdLNs)

after treatment ($n = 3$). **b-c**, Representative immunofluorescence images of BMDCs and CCR2-DC-ANVs in tdLN ($n = 3$). Scale bar, 50 μm.

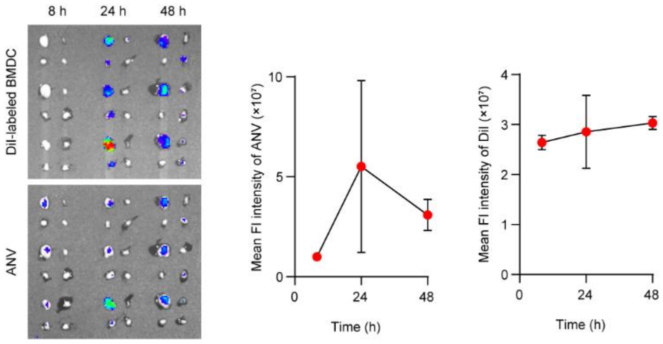

Supplementary Figure 59. Representative fluorescence images and quantitative analysis of tdLNs of tumour-bearing mice after i.v. administration of CCR2-DC-ANVs (DCs and ANVs were labeled with DiI and chlorophyll, respectively) (n=3).

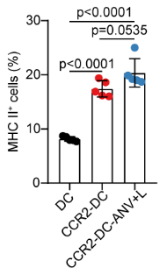

Supplementary Figure 60. Quantitative number of MHC-II^{+} DCs in the tumour (n = 5). Data are mean ± s. d. Statistical analysis was evaluated with one-way ANOVA.

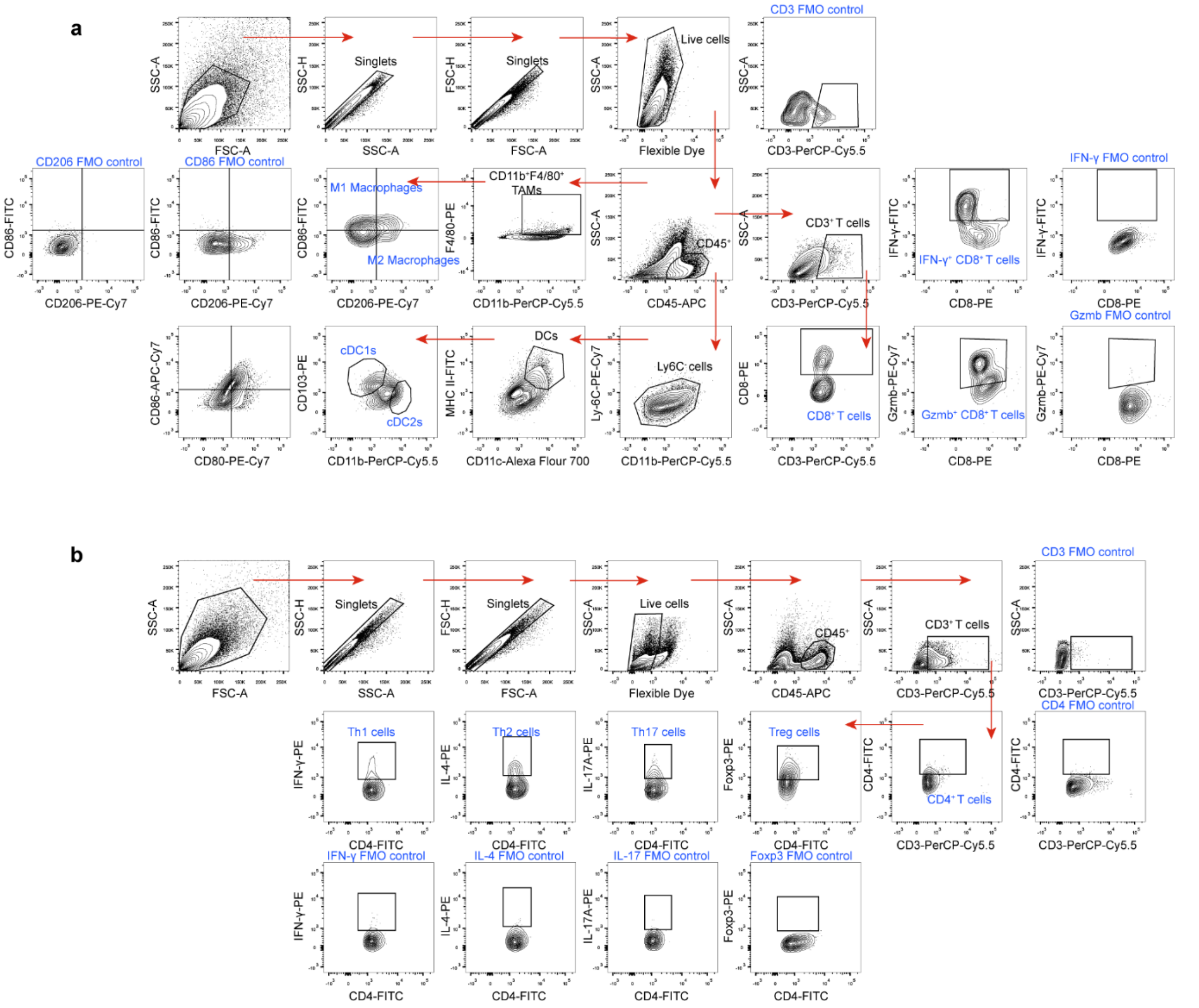

Supplementary Figure 61. Flow cytometric gating strategies correspond to Figure 4i-m and Supplementary Figure 62-64 to analyze immune cells in tumours. First, Live cells were gated using Flexible viability dye eFluor 455. Then, SSC-H was plotted vs. SSC-A and FSC-H was plotted vs. FSC-A for singlet population. CD45$^+$ cells were then gated for immune cells. **a**, Gating strategy for cDC1s (CD11c$^+$MHC-II$^+$CD103$^+$CD11b$^-$), cDC2 (CD11c$^+$MHC-II$^+$CD103$^-$CD11b$^+$), M1 macrophages (CD11b$^+$F4/80$^+$CD86$^+$), M2 macrophages (CD11b$^+$F4/80$^+$CD206$^+$) and CD8$^+$ T cells (CD3$^+$CD8$^+$) in tumour. **b**, Gating strategy for Th1 cells (CD3$^+$CD4$^+$IFN-γ$^+$), Th2 cells (CD3$^+$CD4$^+$IL-4$^+$), Th17 cells (CD3$^+$CD4$^+$IL-17$^+$) and Treg cells (CD3$^+$CD4$^+$FoxP3$^+$) in tumour.

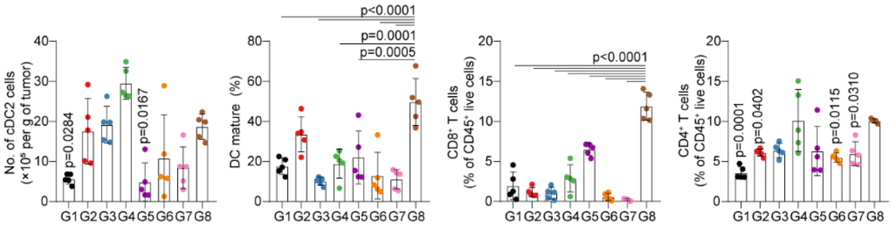

Supplementary Figure 62. Quantitative analysis of cDC2, DC maturation, CD8$^+$ T cells and CD4$^+$ T cells (n=5). G1: PBS, G2: BMDC, G3: CCR2-DC, G4: ANV+L, G5: DC-ANV+L, G6: CCR2-DC+ANV+L, G7: CCR2-DC-ANV, G8: CCR2-DC-ANV+L. Data are mean ± s. d. Statistical analysis was evaluated with one-way ANOVA.

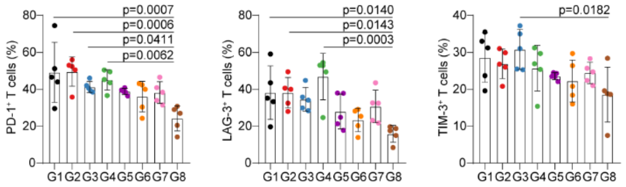

Supplementary Figure 63. Quantitative analysis of PD-1, TIM-3 and LAG-3 expression in CD8$^+$ T cells in tumour (n = 5). G1: PBS, G2: BMDC, G3: CCR2-DC, G4: ANV+L, G5: DC-ANV+L, G6: CCR2-DC+ANV+L, G7: CCR2-DC-ANV, G8: CCR2-DC-ANV+L. Data are mean ± s. d. Statistical analysis was evaluated with one-way ANOVA.

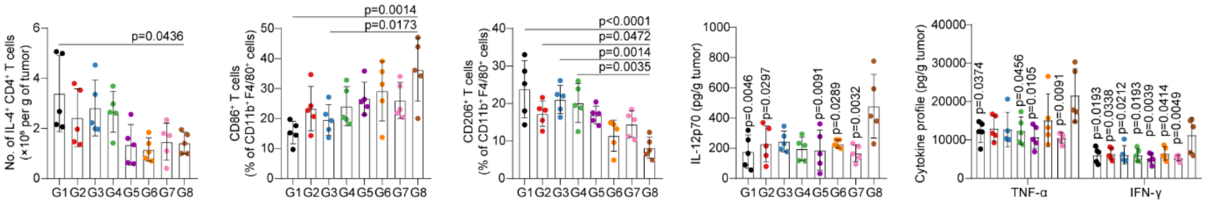

Supplementary Figure 64. Quantitative analysis of Th2, Th17, M1-like tumour-associated macrophages (TAMs), M2-kile TAMs and cytokine profiles in the tumour supernatant measured by ELISA (n=5). G1: PBS, G2: BMDC, G3: CCR2-DC, G4: ANV+L, G5: DC-ANV+L, G6: CCR2-DC+ANV+L, G7: CCR2-DC-ANV, G8: CCR2-DC-ANV+L. Data are mean ± s. d. Statistical analysis was evaluated with one-way ANOVA.

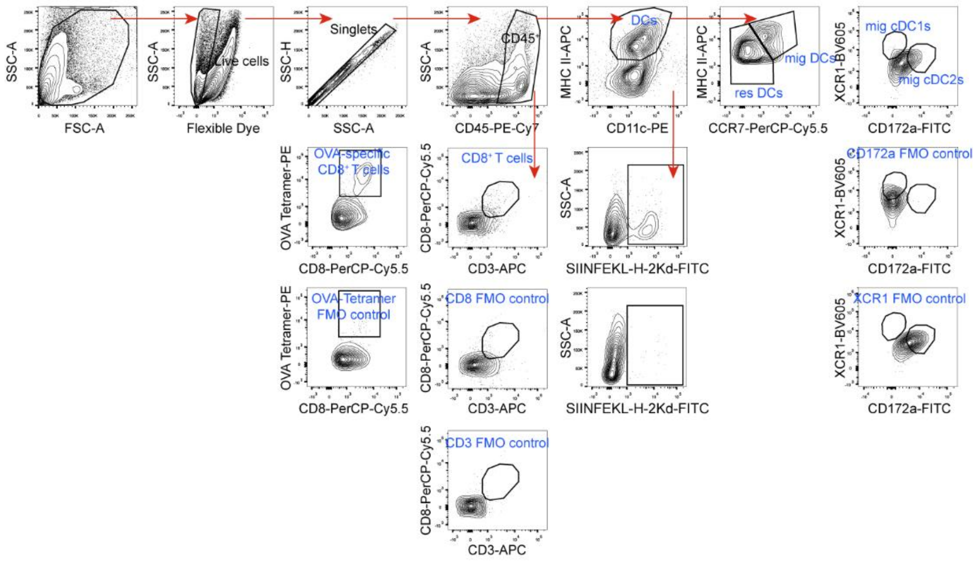

Supplementary Figure 65. Flow cytometric gating strategies correspond to Figure 4n-s and

Supplementary Figure 66 to analyze mig-cDCs and antigen-specific T cells in tumours. First, Live cells were gated using Flexible viability dye eFluor 455. Then, SSC-H was plotted vs. SSC-A and FSC-H was plotted vs. FSC-A for singlet population. $CD45^{+}$ cells were then gated for immune cells. Gating strategy for mig-cDC1s ($CD11c^{+}MHC\text{-}II^{+}CCR7^{+}XCR1^{+}CD172a^{-}$) and mig-cDC2 ($CD11c^{+}MHC\text{-}II^{+}CCR7^{+}XCR1^{-}CD172a^{+}$) in tumour.

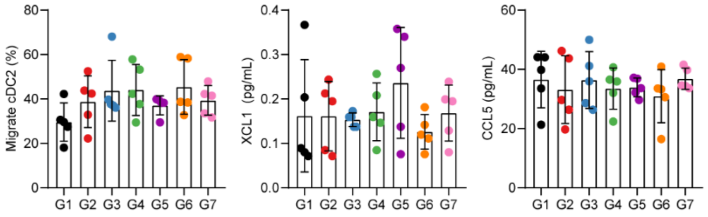

Supplementary Figure 66. Quantitative analysis of mig-cDC2s (**o**) in tumour and XCL1 and CCL5 level in serum (n = 5). G1: PBS, G2: BMDC, G3: CCR2-DC, G4: DC-ANV+L, G5: CCR2-DC+ANV+L, G6: CCR2-DC-ANV, G7: CCR2-DC-ANV+L. Data are mean ± s. d. Statistical analysis was evaluated with one-way ANOVA.

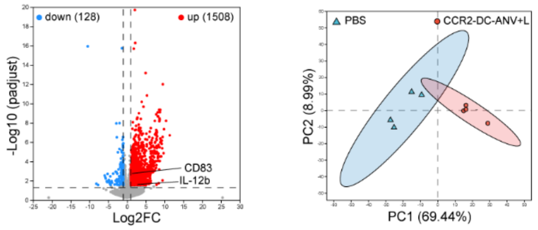

Supplementary Figure 67. Volcano plots and PCA of differentially expressed genes between PBS and CCR2-DC-ANV+L groups ($n = 3$).

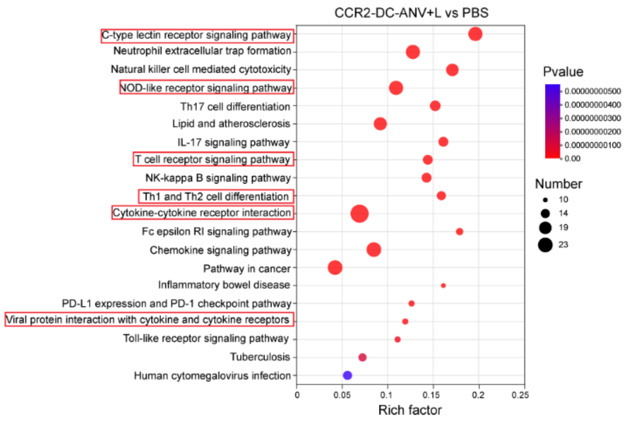

Supplementary Figure 68. GO enrichment analysis (**s**) of differentially expressed genes between PBS and CCR2-DC-ANVs plus light exposure groups ($n = 3$).

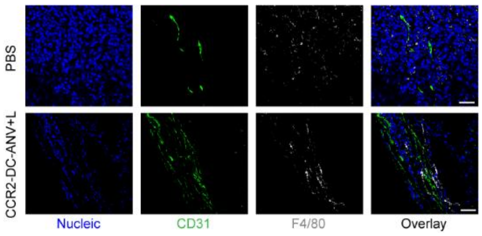

Supplementary Figure 69. Immunofluorescence images of CD31 and F4/80 in tumour ($n = 3$). Scale bar, 50 μm.

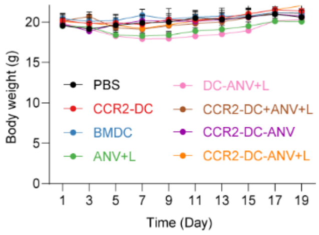

Supplementary Figure 70. Body weight of 4T1 tumour-bearing mice after treatments ($n = 5$).

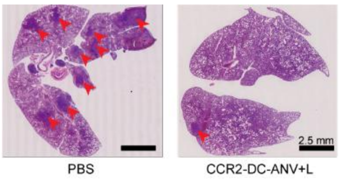

Supplementary Figure 71. Representative H&E staining images of lung metastasis ($n = 3$). Scale bar, 2.5 mm.

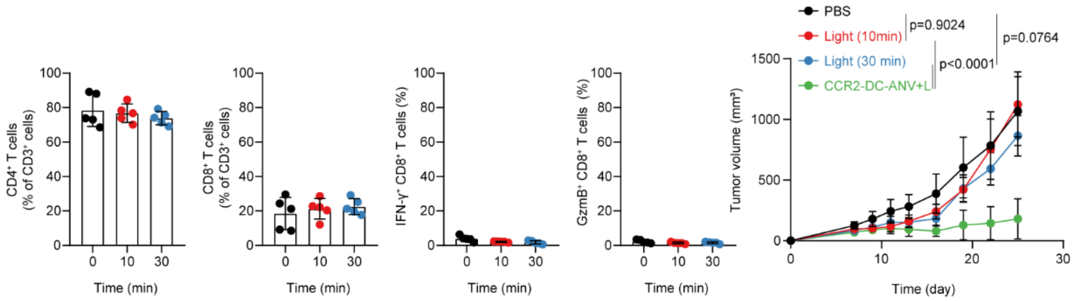

Supplementary Figure 72. Quantitative analysis of immune cells in 4T1 tumours and 4T1 tumour growth curves after light exposure for different duration (n=5). Data are mean ± s. d. Statistical analysis was evaluated with two-way ANOVA.

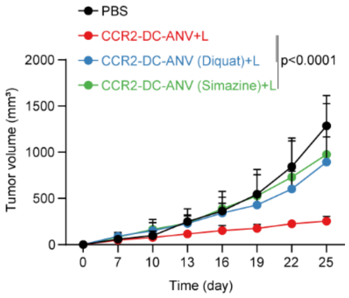

Supplementary Figure 73. 4T1 tumour growth curves after different treatments (n=5). Data are mean ± s. d. Statistical analysis was evaluated with two-way ANOVA.

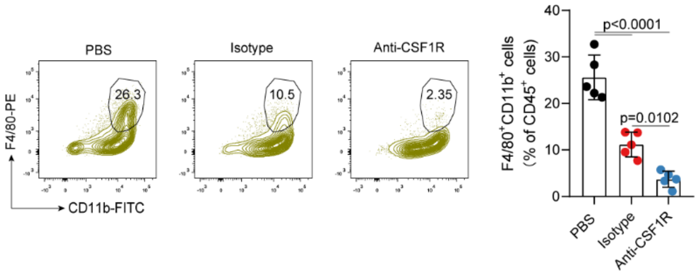

Supplementary Figure 74, Representative flow cytometry plots and quantification of macrophages in tumour-bearing mice (n=5). Data are mean ± s. d. Statistical analysis was evaluated with one-way ANOVA.

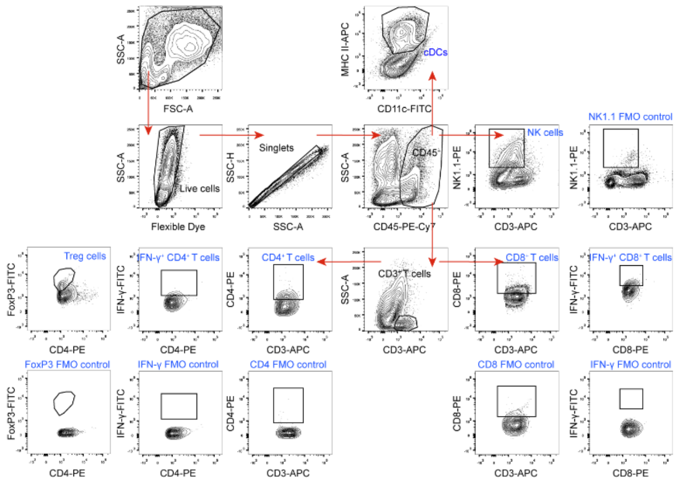

Supplementary Figure 75. Flow cytometric gating strategies correspond to Figure 5g and Supplementary Figure 76 to analyze tumour-infiltrating immune cells in *Batf3*$^{-/-}$ mice.

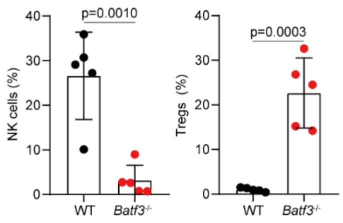

Supplementary Figure 76. Quantitative analysis of NK cells and Tregs in *Batf3*$^{-/-}$ mice (n=5). Data are mean ± s. d. Statistical analysis was evaluated with student's two-tailed unpaired *t*-test.

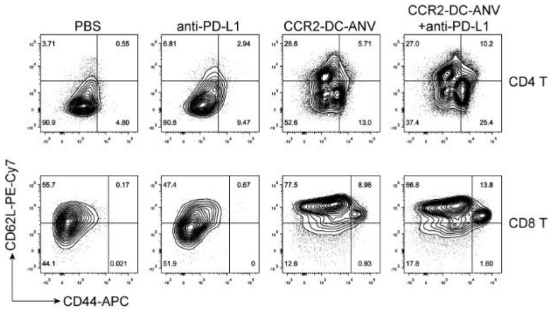

Supplementary Figure 77. Representative flow cytometry plots correspond to Figure 5q-r of T_{em} and T_{cm} in spleen ($n = 5$).

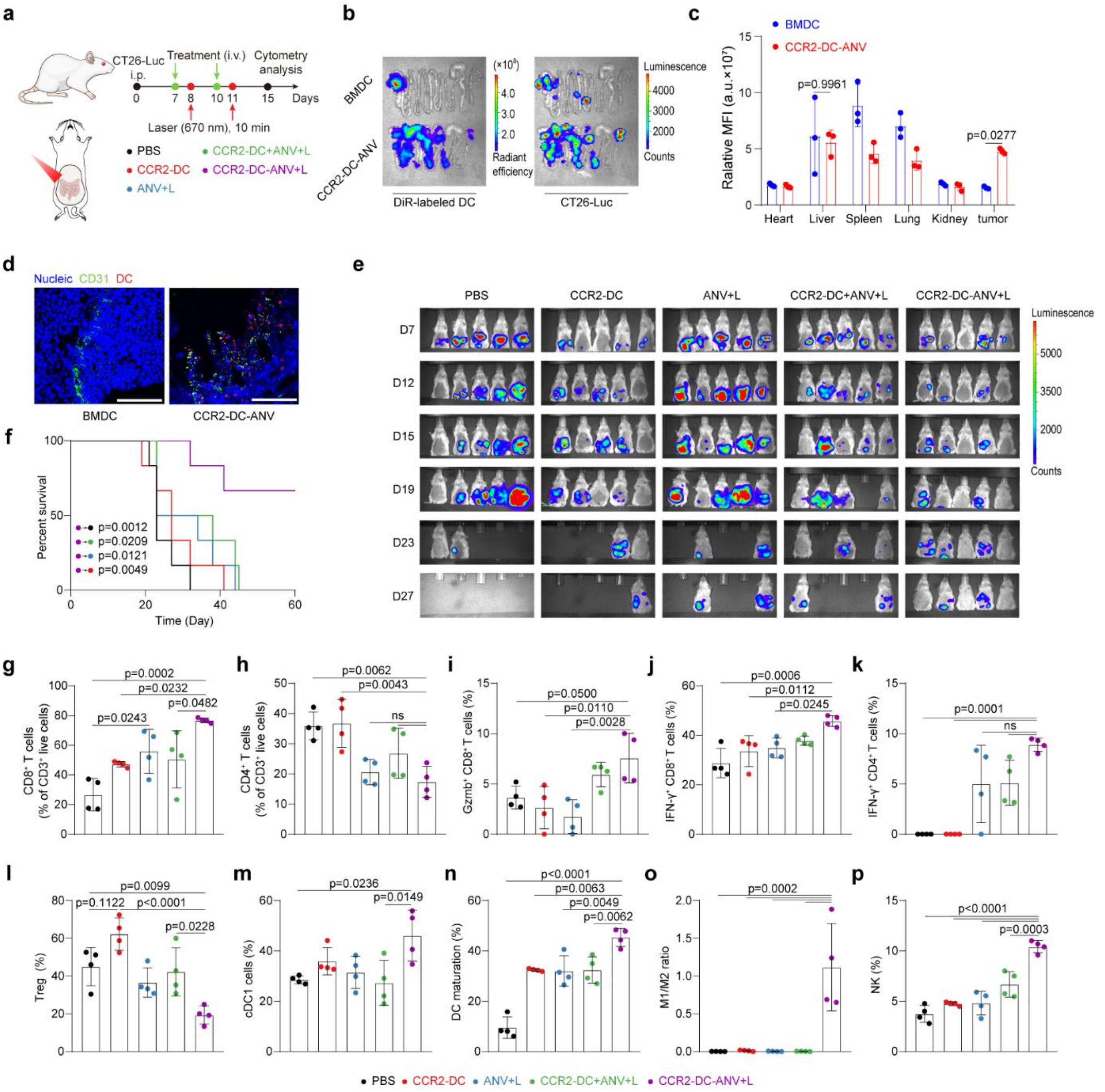

Supplementary Figure 78. Antitumour efficacy of CCR2-DC-ANVs in disseminated tumour-bearing mouse models. a, Scheme of the experimental design and light exposure method for treating abdominal dissemination CT26-Luc tumour using CCR2-DC-ANVs. **b-c**, Representative images of fluorescence and bioluminescence (**b**) in abdominal disseminated tumours and quantitative analysis of fluorescence (**c**) in different organs after CCR2-DC-ANV treatment (n=3). **d**, Representative immunofluorescence images of BMDCs and CCR2-DC-ANVs in tumour ($n = 3$). Scale bar, 100 µm. **e**, In vivo bioluminescence images of abdominal dissemination CT26-Luc tumour-bearing mice ($n = 5$). **f**, Survival curves of CT26-Luc tumour-bearing mice after treatment (n=6). **g-p**, Quantitative analysis of different immune cells in abdominal

dissemination CT26-Luc tumour (n=4). Data are mean ± s. d. Statistical analysis was evaluated with one-way ANOVA (**g-p**), two-way ANOVA (**c**) and log-rank (Mantel–Cox) test (**f**).

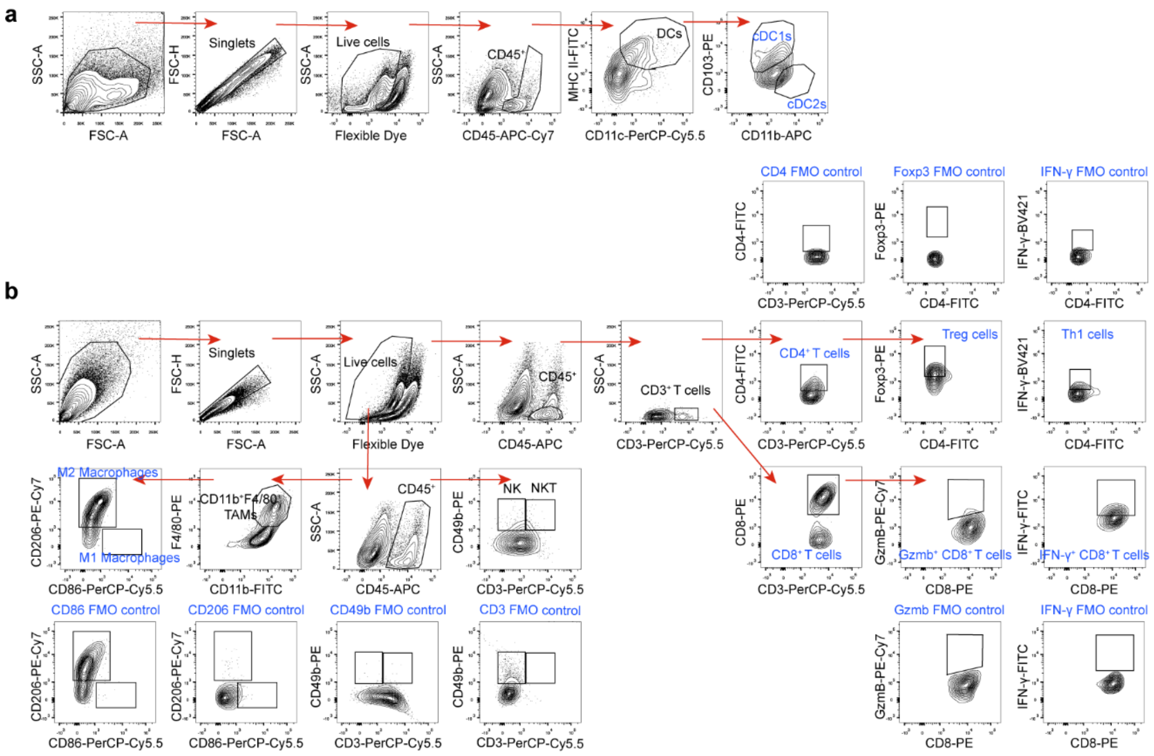

Supplementary Figure 79. Gating strategies correspond to Supplementary Figure 78 to analyze immune cells in CT26-Luc tumours. First, Live cells were gated using Flexible viability dye eFluor 455. Then, SSC-H was plotted vs. SSC-A and FSC-H was plotted vs. FSC-A for singlet population. $CD45^+$ cells were then gated for immune cells. **a**, Gating strategy for cDC1s ($CD11c^+MHC\text{-}II^+CD103^+CD11b^-$) and cDC2 ($CD11c^+MHC\text{-}II^+CD103^-CD11b^+$) in tumour. **b**, Gating strategy for M1 macrophages ($CD11b^+F4/80^+CD86^+$), M2 macrophages ($CD11b^+F4/80^+CD206^+$), $CD8^+$ T cells ($CD3^+CD8^+$), NK cells ($CD3^-CD49b^+$), Th1 cells ($CD3^+CD4^+IFN\text{-}\gamma^+$) and Treg cells ($CD3^+CD4^+FoxP3^+$) and Treg cells ($CD3^+CD4^+FoxP3^+$) in tumour.

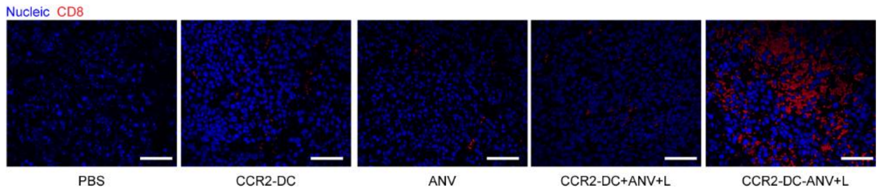

Supplementary Figure 80. Representative immunofluorescence images of $CD8^+$ T cells in tumour ($n = 3$). Scale bar, 50 μm.

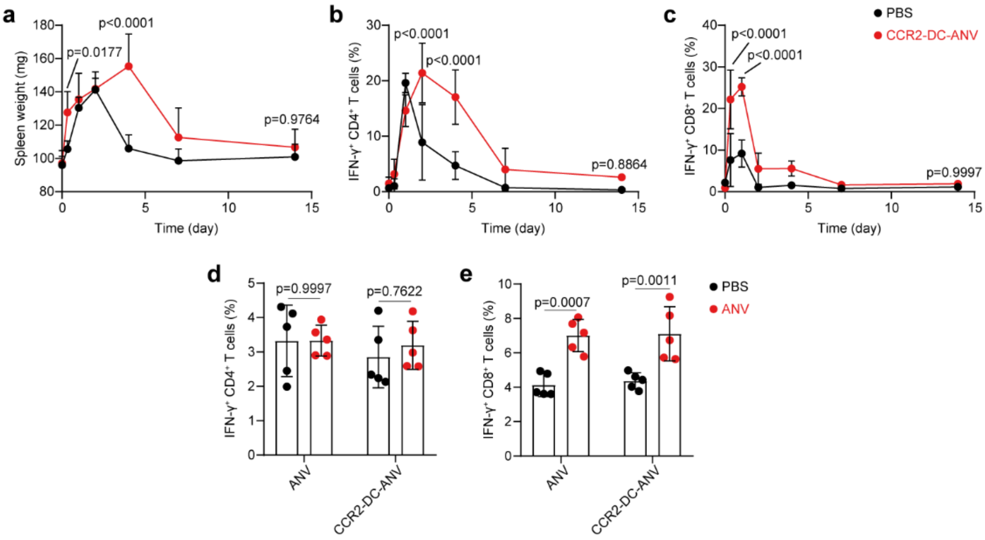

Supplementary Figure 81. a-c, Quantitative analysis of spleen weight (**a**), IFN-γ^{+} CD4^{+} T cells (**b**) and IFN-γ^{+} CD8^{+} T cells (**c**) in spleen of mice post-treated with CCR2-DC-ANVs and ANVs for different times (n=3). **d-e,** Quantitative analysis of IFN-γ^{+} CD4^{+} T cells (**d**) and IFN-γ^{+} CD8^{+} T cells (**e**) (n=5). Splenic T cells were isolated from mice post-treated with CCR2-DC-ANVs and ANVs for different times and re-incubated with ANVs for 48 h. Data are mean ± s. d. Statistical analysis was evaluated with two-way ANOVA.

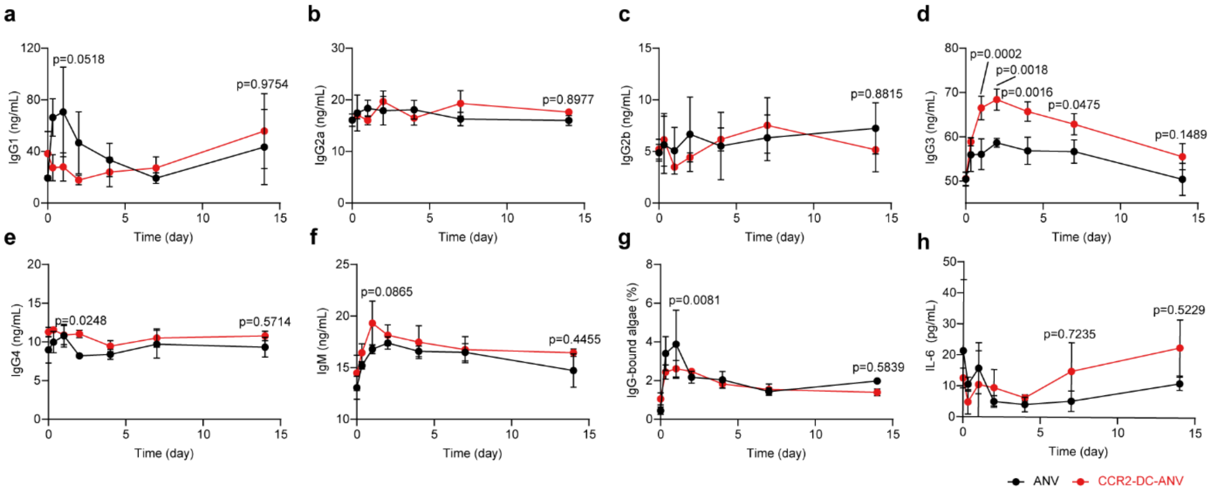

Supplementary Figure 82. a-f, Quantitative analysis of secreted antibodies in serum (n=3). **g**, Quantitative analysis of IgG-bound algae after treated with serum of mice post-treated with CCR2-DC-ANVs and ANVs for different times (n=3). **h**, Quantitative analysis of IL-6 in serum (n=3). Data are mean ± s. d. Statistical analysis was evaluated with two-way ANOVA.

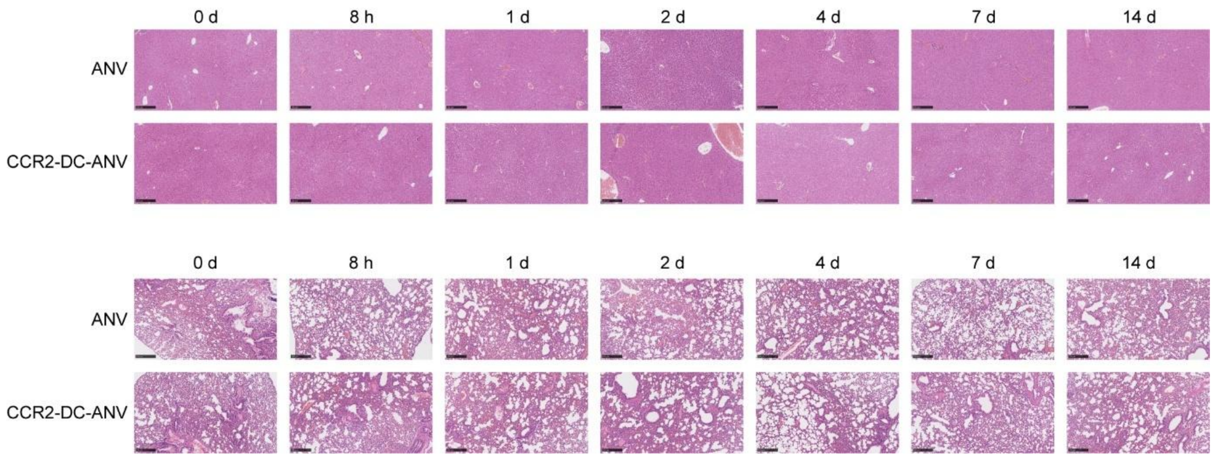

Supplementary Figure 83. Representative H&E staining images of liver and lung ($n = 3$). Scale bar, 250 μm.

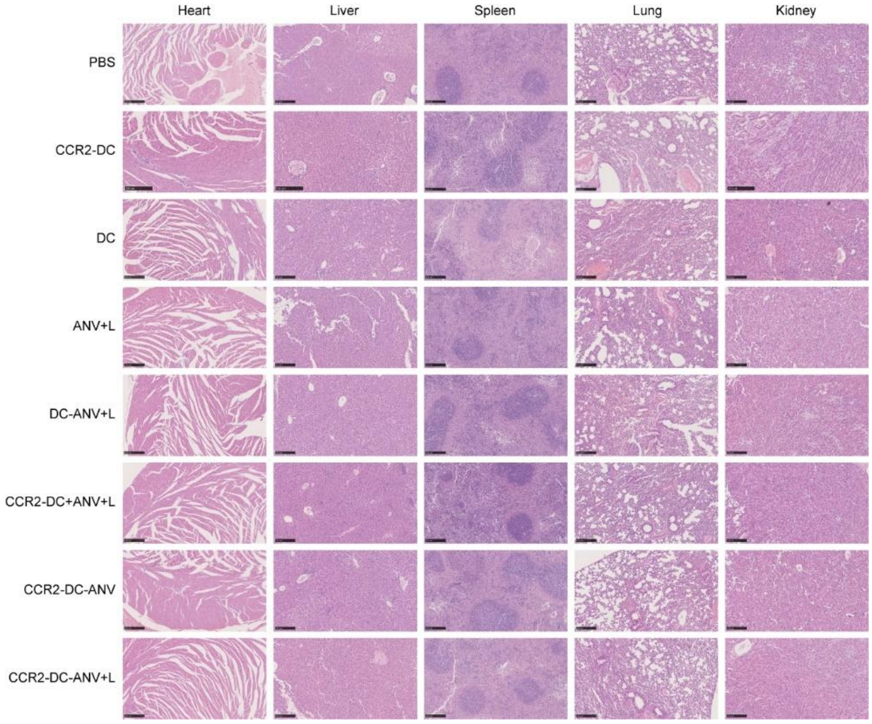

Supplementary Figure 84. Representative H&E staining images of major organs ($n = 3$). Scale bar, 250 μm.

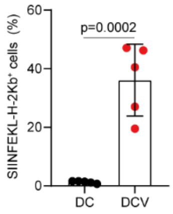

Supplementary Figure 85. Quantitative analysis of SIINFEKL-H-2Kb$^+$ cells in DC vaccines (n=5). Data are mean ± s. d. Statistical analysis was evaluated with student's two-tailed unpaired *t*-test.

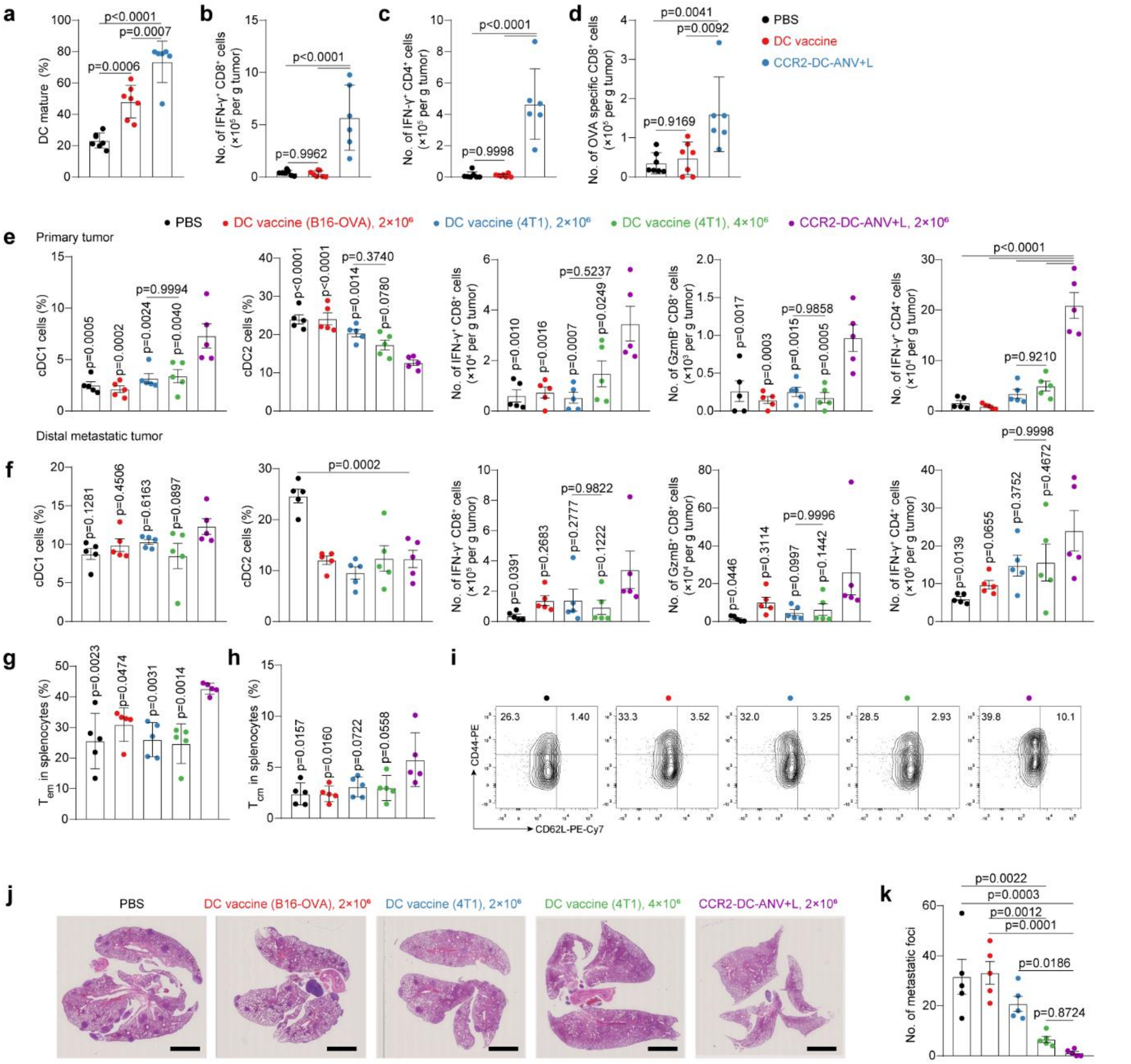

Supplementary Figure 86. Antitumour efficacy of CCR2-DC-ANVs compare to lysate-pulsed autologous DC vaccines. a-d, Quantitative analysis of DC mature (**a**), IFN-γ^{+} CD8^{+} T cells (**b**), IFN-γ^{+} CD4^{+} T cells (**c**) and OVA-specific CD8^{+} T cells (**d**) in B16-OVA tumour (n=6) **e-f**, Quantitative analysis of cDC1, cDC2, IFN-γ^{+} CD8^{+} T cells, GzmB^{+} CD8^{+} T cells and IFN-γ^{+} CD4^{+} T cells in primary tumour (**e**) and distal metastatic tumour (**f**) (n=5). **g-h**, Quantitative analysis of Tem (CD44^{+} CD62L^{-}) and Tcm (CD44^{+} CD62L^{+}) in spleen (n=5). **i**, Representative cytometry plots of Tem and Tcm. **j**, Representative H&E staining images of lung metastasis (*n* = 5). Scale bar, 2.5 mm. **k**, Total number of lung surface metastases (n=5). Scale bar, 100 μm. Data are mean ± s. d. Statistical analysis was evaluated with one-way ANOVA.

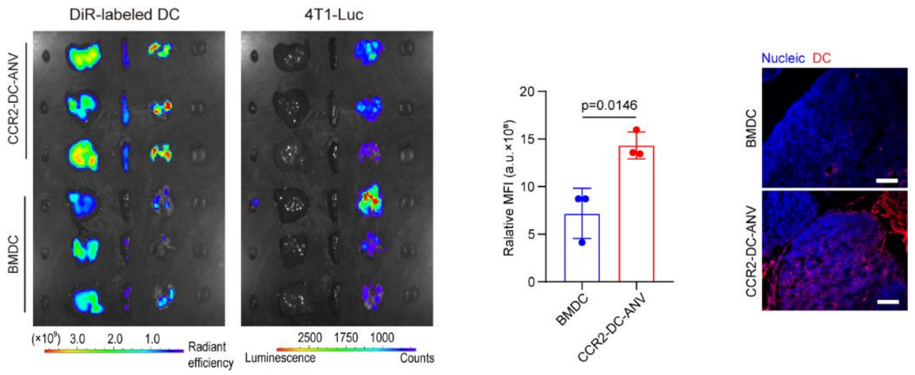

Supplementary Figure 87. Representative fluorescence images of major organs of tumour-bearing mice after i.v. administration of CCR2-DC-ANVs and BMDCs. Quantitative fluorescence analysis of lungs and representative immunofluorescence images of DC infiltration within lung metastasis lesion were evaluated ($n = 3$). Data are mean ± s. d. Statistical analysis was evaluated with student's two-tailed unpaired t-test.

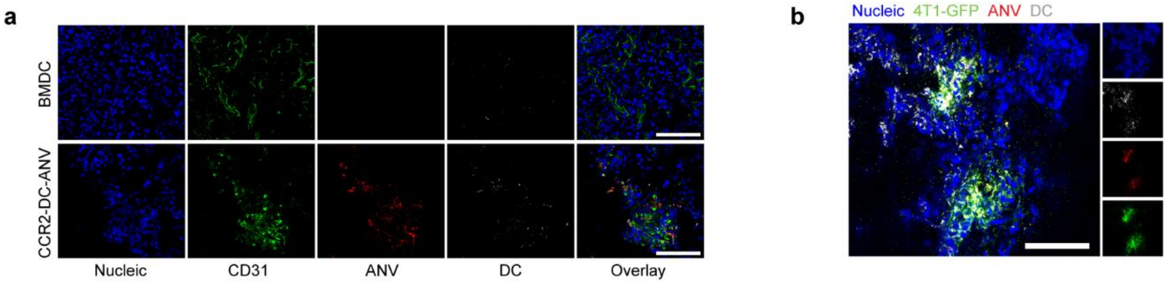

Supplementary Figure 88. **a**, Representative immunofluorescence images of BMDCs and CCR2-DC-ANVs in lung metastasis lesion ($n = 3$). Scale bar, 100 µm. **b**, Fluorescent visualization of CCR2-DC-ANV localization in lung metastasis lesion ($n = 3$). Tumour cells were labeled by GFP. Scale bar, 100 µm.

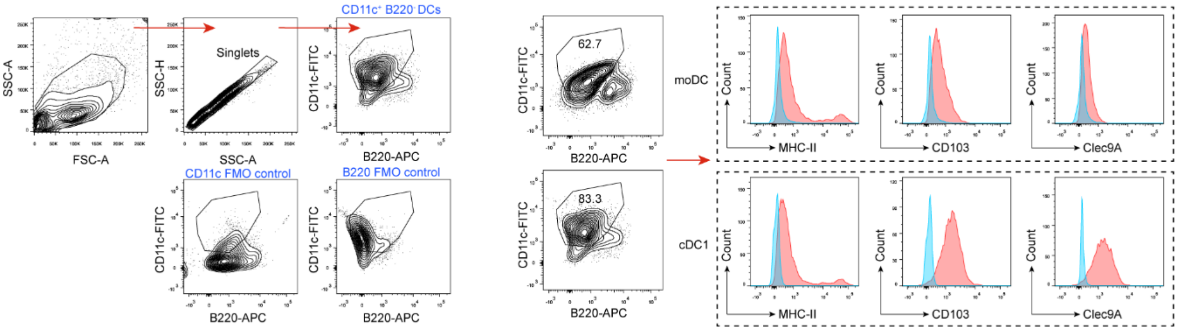

Supplementary Figure 89. Flow cytometric gating strategy of moDCs and cDC1s and representative cytometry histograms of MHC-II, CD103 and Clec9A in moDCs and cDC1s ($n = 5$).

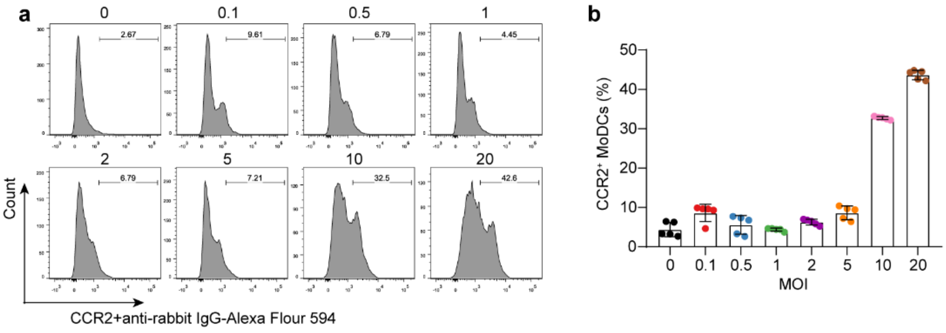

Supplementary Figure 90. Representative cytometry plots and quantitative analysis of CCR2 expression on CCR2-moDC-ANV ($n = 5$).

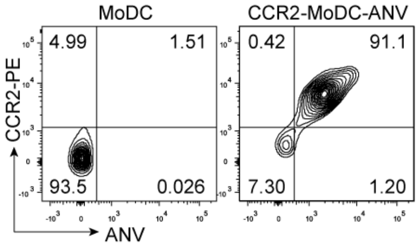

Supplementary Figure 91. Representative cytometry plots of CCR2-moDC-ANVs ($n = 3$).

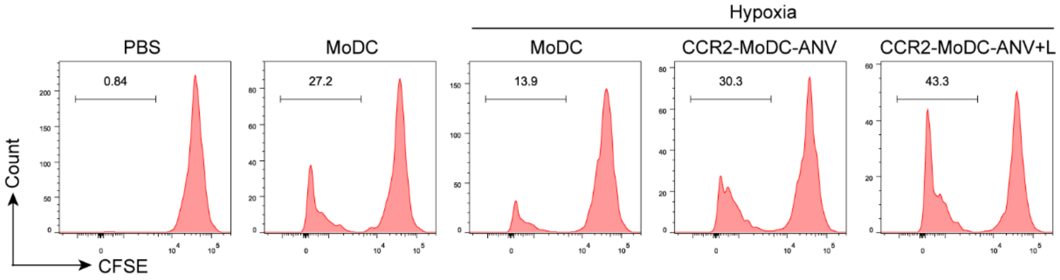

Supplementary Figure 92. Representative cytometry histograms of T cell proliferation after incubation with CCR2-DC-ANVs and moDCs ($n = 3$).

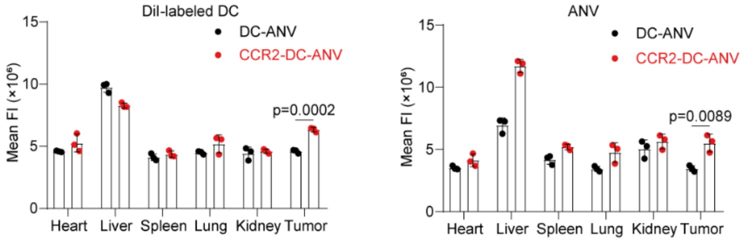

Supplementary Figure 93. Quantitative analysis of major organs of humanized CDX tumour-bearing mice after i.v. administration of CCR2-DC-ANVs (DCs and ANVs were labeled with DiI and chlorophyll, respectively) (n=3). Data are mean ± s. d. Statistical analysis was evaluated with two-way ANOVA.

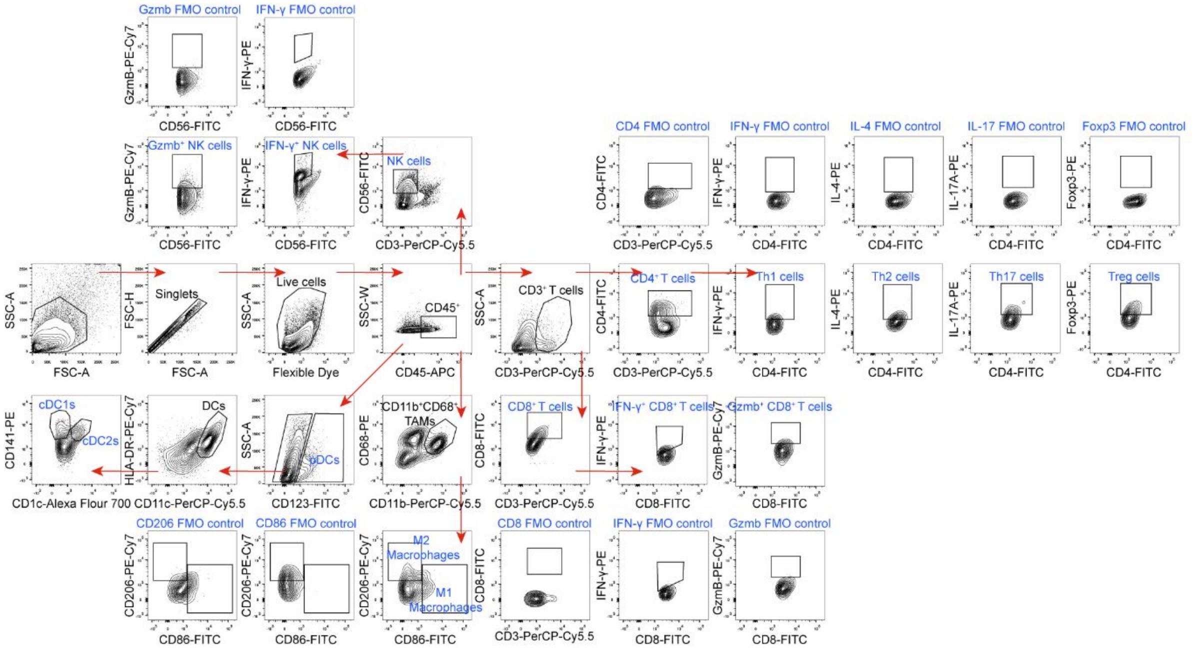

Supplementary Figure 94. Gating strategies to correspond to Figure 7l and Supplementary Figure 95 analyze immune cells in humanized CDX tumours. First, Live cells were gated using Flexible viability dye eFluor 455. Then, SSC-H was plotted vs. SSC-A and FSC-H was plotted vs. FSC-A for singlet population. CD45^{+} cells were then gated for immune cells. Gating strategy for cDC1s (CD123^{-}CD11c^{+}HLA-DR^{+}CD141^{+}CD1c^{-}), cDC2 (CD123^{-}CD11c^{+}HLA-DR^{+}CD141^{-}CD1c^{+}), M1 macrophages (CD11b^{+}CD68^{+}CD86^{+}), M2 macrophages (CD11b^{+}CD68^{+}CD206^{+}), CD8^{+} T cells (CD3^{+}CD8^{+}), NK cells (CD3^{-}CD56^{+}), Th1 cells (CD3^{+}CD4^{+}IFN-γ^{+}), Th2 cells (CD3^{+}CD4^{+}IL-4^{+}), Th17 cells (CD3^{+}CD4^{+}IL-17^{+}) and Treg cells (CD3^{+}CD4^{+}FoxP3in tumour were presented.

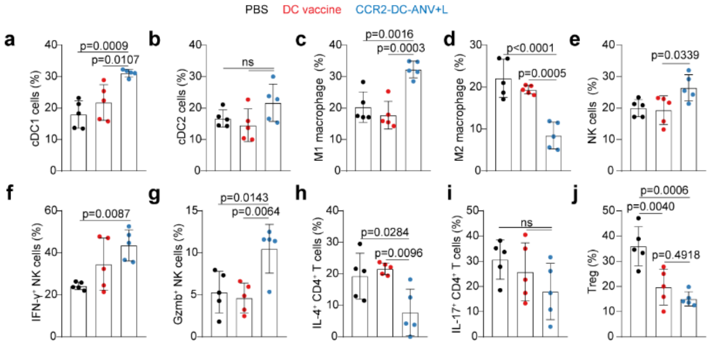

Supplementary Figure 95. a-j, Quantitative analysis of cDC1, cDC2, M1-like TAMs, M2-like TAMs, NK cells, IFN-γ^{+} NK cells, GzmB^{+} NK cells, Th2, Th17 and Treg cells in humanized CDX tumour (n=5). Data are mean ± s. d. Statistical analysis was evaluated with one-way ANOVA.

Supplementary Table 1. Antibodies used in the experiment.

Antibodies	Source	Identifier	Dilution
Fixable Viability Dye eFluor 455	eBioscience	cat. no. 65-0868-14	1:1000
Anti-mouse CD16/32	eBioscience	cat. no. 14-0161-82	1:1000
FITC anti-mouse IgG	BioLegend	cat. no. 406001	1:100
PE anti-mouse CCR2	BioLegend	cat. no. 150609	1:100
APC anti-mouse CCR2	BioLegend	cat. no. 150627	1:100
APC-Cy7 anti-mouse CD45	BioLegend	cat. no. 103116	1:100
APC anti-mouse CD45	eBioscience	cat. no. 17-0451-82	1:100
PE-eFluor 610 anti-mouse CCR7	eBioscience	cat. no. 61-1971-82	1:100
Alexa Flour 700 anti-mouse CD11c	BioLegend	cat. no. 117319	1:100
PE anti-mouse CD11c	BioLegend	cat. no. 117307	1:100
APC anti-mouse CD11c	Tonbo Biosciences	cat. no. 20-0114-u025	1:100
PerCP-Cy5.5 anti-mouse CD11b	Tonbo Biosciences	cat. no. 65-0112-u100	1:100
FITC anti-mouse CD11b	eBioscience	cat. no. 11-0112-82	1:100
Alexa Flour 700 anti-mouse CD11b	eBioscience	cat. no. 56-0112-80	1:100
PE anti-mouse CD80	Tonbo Biosciences	cat. no. 50-0801-u025	1:100
PE-Cy7 anti-mouse CD80	Tonbo Biosciences	cat. no. 60-0801-u025	1:100
APC-Cy7 anti-mouse CD86	BioLegend	cat. no. 105029	1:100
FITC anti-mouse CD86	Tonbo Biosciences	cat. no. 35-0862-u025	1:100
APC-anti-CD86	eBioscience	cat. no. 17-0862-82	1:100
PE anti-mouse MHC I	eBioscience	cat. no. 12-5958-82	1:100
FITC anti-mouse MHC I	eBioscience	cat. no. 11-5958-80	1:100
APC anti-mouse MHC-I	eBioscience	cat. no. 12-5958-82	1:100
FITC anti-mouse MHC II	Tonbo Biosciences	cat. no. 35-5321-u025	1:100
PE anti-mouse MHC II	Tonbo Biosciences	cat. no. 50-5321-u025	1:100
APC anti-mouse CD83	BioLegend	cat. no. 121509	1:100
PE anti-mouse CD40	eBioscience	cat. no. 12-0401-81	1:100
APC anti-mouse H-2Kb bound to SIINFEKL	BioLegend	cat. no. 141605	1:100

FITC anti-mouse H-2Kd bound to SIINFEKL	Beyotime	cat. no. AC1183	1:100
APC anti-mouse H-2Kd bound to SIINFEKL	Beyotime	cat. no. AC0992	1:100
PE anti-mouse H-2Kb bound to SIINFEKL	BioLegend	cat. no. 141603	1:100
FITC anti-mouse CD103	BioLegend	cat. no. 121407	1:100
PE anti-mouse CD103	BioLegend	cat. no. 121405	1:100
FITC anti-mouse CD172a	BioLegend	cat. no. 144006	1:100
PerCP-Cy5.5 anti-mouse XCR1	BioLegend	cat. no. 148207	1:100
BV605 anti-mouse XCR1	BioLegend	cat. no. 148222	1:100
PE-Cy7 anti-mouse Ly-6C	eBioscience	cat. no. 25-5932-80	1:100
FITC anti-mouse CD19	BioLegend	cat. no. 115505	1:100
PerCP-Cy5.5 anti-mouse CD3ε	Tonbo Biosciences	cat. no. 65-0031-u100	1:100
APC anti-mouse CD3ε	eBioscience	cat. no. 17-0037-42	1:100
PE anti-mouse CD8α	BioLegend	cat. no. 100707	1:100
FITC anti-mouse CD8α	Tonbo Biosciences	cat. no. 35-0081-u100	1:100
PerCP-Cy5.5 anti-mouse CD8α	BioLegend	cat. no. 100733	1:100
FITC anti-mouse CD4	Tonbo Biosciences	cat. no. 35-0041-u100	1:100
PE anti-mouse CD4	BioLegend	cat. no.100511	1:100
PerCP-Cy5.5 anti-mouse CD4	BioLegend	cat. no. 100433	1:100
PE anti-mouse H-2Kb OVA Tetramer-SIINFEKL	MBL Life Science	cat. no. TS-5001-1C	1:100
PE anti-mouse IFN-γ	Tonbo Biosciences	cat. no. 50-7311-u025	1:100
FITC anti-mouse IFN-γ	BioLegend	cat. no. 505805	1:100
BV421 anti-mouse IFN-γ	BioLegend	cat. no. 505829	1:100
PE-Cy7 anti-mouse Granzyme B	eBioscience	cat. no. 25-8898-80	1:100
PE anti-mouse IL-4	Tonbo Biosciences	cat. no. 50-7041-u025	1:100
PE anti-mouse IL-17A	eBioscience	cat. no. 12-7177-81	1:100
PE anti-mouse Foxp3	Tonbo Biosciences	cat. no. 50-5773-u025	1:100

FITC anti-mouse Foxp3	eBioscience	cat. no. 11-5773-82	1:100
APC anti-mouse CD279	BioLegend	cat. no. 109111	1:100
APC anti-mouse CD366	eBioscience	cat. no. 17-5870-82	1:100
APC anti-mouse CD223	eBioscience	cat. no. 17-2231-82	1:100
PE anti-mouse CD44	Tonbo Biosciences	cat. no. 50-0441-u025	1:100
PE-Cy7 anti-mouse CD62L	eBioscience	cat. no. 25-0621-81	1:100
FITC anti-mouse CD49b	BioLegend	cat. no. 103503	1:100
APC anti-mouse CD49b	BioLegend	cat. no. 103515	1:100
PE anti-mouse NK1.1	BioLegend	cat. no. 156503	1:100
PE-anti-mouse F4/80	BioLegend	cat. no. 111704	1:100
PE-Cy7 anti-mouse CD206	eBioscience	cat. no. 25-2061-80	1:100
APC-eFlour 780-anti-mouse Ly-6G/Ly-6C	eBioscience	cat. no. 47-5931-80	1:100
Alexa Flour 700-anti-mouse F4/80	eBioscience	cat. no. 56-4801-80	1:100
PE-Cy7-anti-mouse CD326	eBioscience	cat. no. 25-5791-80	1:100
PE-anti-mouse CD88	BioLegend	cat. no. 135805	1:100
Pacific Blue™ anti-mouse Siglec H	BioLegend	cat. no. 129609	1:100
APC anti-mouse CD370	BioLegend	cat. no. 143505	1:100
APC anti-human CD45	eBioscience	cat. no. 17-0459-42	1:100
PerCP-Cy5.5 anti-human CD3	BioLegend	cat. no. 317336	1:100
FITC anti-human CD8α	eBioscience	cat. no. 11-0088-42	1:100
FITC anti-human CD4	eBioscience	cat. no. 11-0049-42	1:100
PE anti-human IFN-γ	eBioscience	cat. no. 12-7319-42	1:100
PE-Cy7 anti-human Granzyme B	BioLegend	cat. no. 372214	1:100
PE anti-human Foxp3	eBioscience	cat. no. 12-4776-42	1:100
PE anti-human IL-4	eBioscience	cat. no. 12-7049-42	1:100
PE anti-human IL-17A	eBioscience	cat. no. 12-7179-42	1:100
FITC anti-human CD56	eBioscience	cat. no. 11-0566-42	1:100
PerCP-eFlour 710 anti-human CD11b	eBioscience	cat. no. 46-0118-42	1:100

			1:100
PE anti-human CD68	eBioscience	cat. no. 12-0689-42	1:100
FITC anti-human CD86	eBioscience	cat. no. 53-0869-42	1:100
PE-Cy7 anti-human CD206	eBioscience	cat. no. 25-2069-42	1:100
PerCP-eFlour 710 anti-human CD11c	eBioscience	cat. no. 46-0116-42	1:100
APC anti-human CD1c	eBioscience	cat. no. 17-0015-42	1:100
PE anti-human CD141	BioLegend	cat. no. 344104	1:100
FITC anti-human CD123	eBioscience	cat. no. 11-1239-42	1:100
PE anti-human CD40	BioLegend	cat. no. 334308	1:100
PE anti-human CD80	BioLegend	cat. no. 305208	1:100
APC anti-human CD83	BioLegend	cat. no. 305312	1:100
PE-Cy7 anti-human HLA-DR	BioLegend	cat. no. 327018	1:100
Anti-mouse CCR2	Abcam	cat. no. ab273050	1:1000
Anti-mouse OPA1	proteintech	cat. no. 27733-1-AP	1:1000
Anti-mouse OPA1	Affinity	cat. no. DF8587	1:1000
Anti-mouse ATP5A1	proteintech	cat. no. 66037-1-Ig	1:1000
Anti-mouse UQCRC2	proteintech	cat. no. 14742-1-AP	1:1000
Anti-mouse SDHB	proteintech	cat. no. 10620-1-AP	1:1000
Anti-mouse NDUFB8	proteintech	cat. no. 67690-1-Ig	1:1000
Anti-mouse IRAK1	Cell Signaling Technology	cat. no. 4504S	1:1000
Anti-mouse NF-κB p65	Cell Signaling Technology	cat. no. 8242T	1:1000
Anti-mouse MAPK p38	Cell Signaling Technology	cat. no. 8690T	1:1000
Anti-mouse MyD88	Cell Signaling Technology	cat. no. 4283S	1:1000
Anti-mouse DRP1	proteintech	cat. no. 81561-1-RR	1:1000
Anti-mouse Phospho-DRP1^{S616}	Abclonal	cat. no. AP1573	1:1000
Anti-mouse Phospho-DRP1^{S637}	Affinity	cat. no. DF2980	1:1000
Anti-mouse MFN1	Affinity	cat. no. DF7543	1:1000
Anti-mouse MFN2	Abclonal	cat. no. A19678	1:1000

Anti-mouse Phospho-mTOR^{S2448}	proteintech	cat. no. 80596-1-RR	1:1000
Anti-mouse Phospho-AKT1^{S473}	proteintech	cat. no. 80462-1-RR	1:1000
Anti-mouse HIF-1α	Affinity	cat. no. AF1009	1:1000
Anti-mouse PERK	Affinity	cat. no. AF5304	1:1000
Anti-mouse Phospho-eIF2α^{S51}	Abclonal	cat. no. AP0692	1:1000
Anti-mouse eIF2α	Abclonal	cat. no. A21221	1:1000
Anti-mouse Phospho-IRE1^{S724}	Abclonal	cat. no. AP1442	1:1000
Anti-mouse XBP-1S	proteintech	cat. no. 83959-5-RR	1:1000
Anti-mouse XBP-1U	proteintech	cat. no. 25997-1-AP	1:1000
Anti-mouse Cleaved Caspase-3	Cell Signaling Technology	cat. no. 9661T	1:1000
Anti-mouse Caspase 7	Cell Signaling Technology	cat. no. 9492T	1:1000
Anti-mouse β-actin	Cell Signaling Technology	cat. no. 13E5	1:1000
Anti-mouse GAPDH	Cell Signaling Technology	cat. no. 14C10	1:1000
Anti-mouse horseradish peroxidase-labelled anti-rabbit IgG (H+L)	Yeasen	cat.no. 33101ES60	1:1000
Anti-CCR2	Abcam	cat. no. ab273050	1:200
Anti-CCR2	Affinity	cat. no. DF7507	1:200
Anti-CD31	Abclonal	cat. no. A0378	1:200
Anti-CCL2/MCP-1	ABclonal	cat. no. A7277	1:200
Anti-CD1c	OriGene	cat. no. TA505411S	1:200
Anti-CD141	ABclonal	cat. no. A22989PM	1:200
Anti-CD83	Abcam	cat. no. ab205343	1:200
Anti-LAMP3	ABclonal	cat. no. A2895	1:200
Anti-CD11c	Abcam	cat. no. ab254183	1:200
Anti-CD8A	Abclonal	cat. no. A23081	1:200
Anti-fibronectin	Affinity	cat. no. AF5335	1:200
Anti-F4/80	Abcam	cat. no. ab6640	1:200
647-conjugated DRP1 (C-	proteintech	cat. no. CL647-12957	1:200

terminal)			
Multi-rAb CoraLite Plus 555-Goat Anti-Rabbit Recombinant Secondary Antibody (H+L)	proteintech	cat. no. RGAR003	1:200
Alexa Fluor 488-labelled goat anti-rabbit IgG (H+L)	Yeasen	cat. no. 33106ES60	1:200
Alexa Fluor 594-labelled donkey anti-rabbit IgG (H+L)	Yeasen	cat. no. 34212ES60	1:200
