# Supplementary material for: A plant vesicle-dendritic cell chimera for enhancing cancer immunotherapy
Source: Nat Commun. 2026 May 28;17:6944. doi: 10.1038/s41467-026-73788-5 (PMC13388912; doi:10.1038/s41467-026-73788-5)
Supplement: Supplementary file 2 — Description of Additional Supplementary Files [file 41467_2026_73788_MOESM2_ESM.pdf]

Description of Additional Supplementary Files

Supplementary Data 1. Summary of algae-related antigenic peptides presented by MHC II molecules in CCR2-DC-ANV (n = 3).

Supplementary Data 2. Summary of algae-related antigenic peptides presented by MHC I molecules in CCR2-DC-ANV (n = 3).
