# Supplementary material for: A plant vesicle-dendritic cell chimera for enhancing cancer immunotherapy
Source: Nat Commun. 2026 May 28;17:6944. doi: 10.1038/s41467-026-73788-5 (PMC13388912; doi:10.1038/s41467-026-73788-5)
Supplement: Supplementary file 4 — Reporting Summary [file 41467_2026_73788_MOESM4_ESM.pdf]

nature portfolio

Corresponding author(s): Qi Zhao
Dangge Wang
Yaping Li

Last updated by author(s): May 18, 2026

Reporting Summary

Nature Portfolio wishes to improve the reproducibility of the work that we publish. This form provides structure for consistency and transparency in reporting. For further information on Nature Portfolio policies, see our Editorial Policies and the Editorial Policy Checklist.

Statistics

For all statistical analyses, confirm that the following items are present in the figure legend, table legend, main text, or Methods section.

n/a	Confirmed	
☐	☒	The exact sample size (n) for each experimental group/condition, given as a discrete number and unit of measurement
☐	☒	A statement on whether measurements were taken from distinct samples or whether the same sample was measured repeatedly
☐	☒	The statistical test(s) used AND whether they are one- or two-sided<br>*Only common tests should be described solely by name; describe more complex techniques in the Methods section.*
☒	☐	A description of all covariates tested
☒	☐	A description of any assumptions or corrections, such as tests of normality and adjustment for multiple comparisons
☐	☒	A full description of the statistical parameters including central tendency (e.g. means) or other basic estimates (e.g. regression coefficient) AND variation (e.g. standard deviation) or associated estimates of uncertainty (e.g. confidence intervals)
☐	☒	For null hypothesis testing, the test statistic (e.g. F, t, r) with confidence intervals, effect sizes, degrees of freedom and P value noted<br>*Give P values as exact values whenever suitable.*
☒	☐	For Bayesian analysis, information on the choice of priors and Markov chain Monte Carlo settings
☒	☐	For hierarchical and complex designs, identification of the appropriate level for tests and full reporting of outcomes
☒	☐	Estimates of effect sizes (e.g. Cohen's d, Pearson's r), indicating how they were calculated

Our web collection on statistics for biologists contains articles on many of the points above.

Software and code

Policy information about availability of computer code

Data collection	Zetasizer Software (version 7.11) was used for size and ζ-potential measurements.<br>TEM imaging and analysis was used for cyto-TEM data analysis.<br>SEM imaging and analysis was used for cell morphology analysis.<br>UV-VIS spectrophotometer (UV-2600, SHIMADZU) was used to analyze loading efficiency of liposomes.<br>BD LSRFortessa Cell Analyzer (BD Biosciences) was used for flow cytometry data collection.<br>Olympus IX81 confocal laser-scanning microscope was used for transwell analysis.<br>TCS SP8 confocal microscope (Leica, Germany) and LAS X software version (3.0.0.15697) was used for confocal laser scanning microscope data collection.<br>Imaging Lab v5.2.1 software was used for western blot data collection.<br>Spectrum Living Image 4.0 Software was used for in vivo/ex vivo fluorescense analysis.
Data analysis	All statistical analyses were performed on Graphpad Prism (version 8.0.1). The flow cytometry data were analyzed using FlowJo software package (version 10.6.2; BD, USA). Living image software (Perkin Elmer) was used to analyze bioluminescent and fluorescent images. The patient sample gene expression data were analyzed in TIMER (version 1.0). The patient sample survival curves were analyzed in GEPIA (version 1.0), which was developed by R (version 3.3.2) and Perl (version 5.22.1) programs, and processed by PHP scripts (version 7.0.13). The proteomic data,transcriptomics data and metabonomics data were analyszed using Spectronaut software (Version 14) and performed with the Majorbio Cloud platform (https://cloud.majorbio.com). The histological section images were analyzed using CaseViewer (2.0).

For manuscripts utilizing custom algorithms or software that are central to the research but not yet described in published literature, software must be made available to editors and reviewers. We strongly encourage code deposition in a community repository (e.g. GitHub). See the Nature Portfolio guidelines for submitting code & software for further information.

Data

Policy information about availability of data

All manuscripts must include a data availability statement. This statement should provide the following information, where applicable:

- Accession codes, unique identifiers, or web links for publicly available datasets
- A description of any restrictions on data availability
- For clinical datasets or third party data, please ensure that the statement adheres to our policy

The relationship of CCL2 level and DC infiltration level was quantified using the myeloid DC scores derived from the xCell algorithm using the TIMER2.0 web platform (https://compbio.cn/timer2/). The survival analysis and Kaplan–Meier curve of breast cancer patients in Fig. 1 were obtained from the GEPIA bioinformatics platform (http://gepia.cancer-pku.cn/). The raw transcriptomic and proteomic data used in this study are available in https://ngdc.cncb.ac.cn/bioproject/ under accession code CRA022538, CRA022540 and OMIX016859. Additional information regarding results and methods can be requested from the corresponding authors upon reasonable request.

Research involving human participants, their data, or biological material

Policy information about studies with human participants or human data. See also policy information about sex, gender (identity/presentation), and sexual orientation and race, ethnicity and racism.

Reporting on sex and gender	*Use the terms sex (biological attribute) and gender (shaped by social and cultural circumstances) carefully in order to avoid confusing both terms. Indicate if findings apply to only one sex or gender; describe whether sex and gender were considered in study design; whether sex and/or gender was determined based on self-reporting or assigned and methods used.*<br>*Provide in the source data disaggregated sex and gender data, where this information has been collected, and if consent has been obtained for sharing of individual-level data; provide overall numbers in this Reporting Summary. Please state if this information has not been collected.*<br>*Report sex- and gender-based analyses where performed, justify reasons for lack of sex- and gender-based analysis.*
Reporting on race, ethnicity, or other socially relevant groupings	*Please specify the socially constructed or socially relevant categorization variable(s) used in your manuscript and explain why they were used. Please note that such variables should not be used as proxies for other socially constructed/relevant variables (for example, race or ethnicity should not be used as a proxy for socioeconomic status).*<br>*Provide clear definitions of the relevant terms used, how they were provided (by the participants/respondents, the researchers, or third parties), and the method(s) used to classify people into the different categories (e.g. self-report, census or administrative data, social media data, etc.)*<br>*Please provide details about how you controlled for confounding variables in your analyses.*
Population characteristics	*Describe the covariate-relevant population characteristics of the human research participants (e.g. age, genotypic information, past and current diagnosis and treatment categories). If you filled out the behavioural & social sciences study design questions and have nothing to add here, write "See above."*
Recruitment	*Describe how participants were recruited. Outline any potential self-selection bias or other biases that may be present and how these are likely to impact results.*
Ethics oversight	Tissue microarrays (TMAs) were purchased from Yanteng Biotechnology Co., Ltd. (Shanghai, China) and Wknowbio Technology Co., Ltd. (Shanghai, China) with approval number SHYJS-CP-240103.<br>Peripheral blood samples from healthy donor were obtained with written informed consent from all participants and ethical approval from the Ethics Committee of Shanghai General Hospital (approval number 2025SQ066)

Note that full information on the approval of the study protocol must also be provided in the manuscript.

Field-specific reporting

Please select the one below that is the best fit for your research. If you are not sure, read the appropriate sections before making your selection.

☒ Life sciences ☐ Behavioural & social sciences ☐ Ecological, evolutionary & environmental sciences

For a reference copy of the document with all sections, see nature.com/documents/nr-reporting-summary-flat.pdf

Life sciences study design

All studies must disclose on these points even when the disclosure is negative.

Sample size	No sample size calculation was performed, but our sample sizes are consistent with the previously published works on Nat Cancer (Nat Cancer. 2020 Sep;1(9):882-893; Nat Cancer. 2022 Mar;3(3):303-317; Nat Cancer. 2022 Jul;3(7):866-884). The detailed sample sizes are clearly reported in the Figure Legend and Methods. Besides, we were strict compliance with sample size requirements for determining statistical significance.
Data exclusions	No data were excluded from the analysis.
Replication	The experiments were performed in biological and/or technical replicates, as indicated in the figure legends. The in vitro experiments were

executed with at least 3 biological replicates for each group. The immunological mechanism in animals were conducted on 4T1 and CT26 models (n = 5 biological replicates for each group). The anti-tumour studies were replicated on different models (n = 5 or 6 biological replicates for each group).

Randomization	Animal groups were randomized by body weight. In the other experiments, samples were randomly assigned to experimental groups.
Blinding	No blinding assay was performed and no participant groups were involved in this manuscript. The cryo-TEM and tissue microarrays were imaged and analyzed by an independent operator. The fluorescence images were captured using a computer-controlled instrument with the same setting and the image processing parameters were coherent in an independent experiment. Other experiments were conducted and analyzed by only one operator.

Reporting for specific materials, systems and methods

We require information from authors about some types of materials, experimental systems and methods used in many studies. Here, indicate whether each material, system or method listed is relevant to your study. If you are not sure if a list item applies to your research, read the appropriate section before selecting a response.

Materials & experimental systems

n/a	Involved in the study	
[ ]	[x]	Antibodies
[ ]	[x]	Eukaryotic cell lines
[x]	[ ]	Palaeontology and archaeology
[ ]	[x]	Animals and other organisms
[x]	[ ]	Clinical data
[x]	[ ]	Dual use research of concern
[ ]	[x]	Plants

Methods

n/a	Involved in the study	
[x]	[ ]	ChIP-seq
[ ]	[x]	Flow cytometry
[x]	[ ]	MRI-based neuroimaging

Antibodies

Antibodies used

The detailed antibody information (including company, catalogue number, clone, and dilution) were listed as follows:
Flow cytometry analysis:
Fixable Viability Dye eFluor 455 (eBioscience: cat. no. 65-0868-14, 1:100) was used to recognize and exclude the dead cell populations. Anti-mouse CD16/32 (eBioscience: cat. no. 14-0161-82) antibody was used for Fc blockade. Cells were respectively stained with the following antibodies: FITC anti-mouse IgG (BioLegend: cat. no. 406001, 1:100), PE anti-mouse CCR2 (BioLegend: cat. no. 150609, 1:100), APC anti-mouse CCR2 (BioLegend: cat. no. 150627, 1:100), APC-Cy7 anti-mouse CD45 (BioLegend: cat. no. 103116, 1:100), APC anti-mouse CD45 (eBioscience: cat. no. 17-0451-82, 1:100), PE-eFluor 610 anti-mouse CCR7 (eBioscience: cat. no. 61-1971-82, 1:100), Alexa Flour 700 anti-mouse CD11c (BioLegend: cat. no. 117319, 1:100), PE anti-mouse CD11c (BioLegend: cat. no. 117307, 1:100), APC anti-mouse CD11c (Tonbo Biosciences, cat. no. 20-0114-u025, 1:100), PerCP-Cy5.5 anti-mouse CD11b (Tonbo Biosciences, cat. no. 65-0112-u100, 1:100), FITC anti-mouse CD11b (eBioscience: cat. no. 11-0112-82, 1:100), Alexa Flour 700 anti-mouse CD11b (eBioscience: cat. no. 56-0112-80, 1:100), PE anti-mouse CD80 (Tonbo Biosciences, cat. no. 50-0801-u025, 1:100), PE-Cy7 anti-mouse CD80 (Tonbo Biosciences, cat. no. 60-0801-u025, 1:100), APC-Cy7 anti-mouse CD86 (BioLegend: cat. no. 105029, 1:100), FITC anti-mouse CD86 (Tonbo Biosciences, cat. no. 35-0862-u025, 1:100), APC-anti-CD86 (eBioscience: cat. no. 17-0862-82, 1:100), PE anti-mouse MHC I (eBioscience: cat. no. 12-5958-82, 1:100), FITC anti-mouse MHC I (eBioscience: cat. no. 11-5958-80, 1:100), APC anti-mouse MHC-I (eBioscience: cat. no. 12-5958-82, 1:100), FITC anti-mouse MHC II (Tonbo Biosciences, cat. no. 35-5321-u025, 1:100), PE anti-mouse MHC II (Tonbo Biosciences, cat. no. 50-5321-u025, 1:100), APC anti-mouse CD83 (BioLegend: cat. no. 121509, 1:100), PE anti-mouse CD40 (eBioscience: cat. no. 12-0401-81, 1:100), APC anti-mouse H-2Kb bound to SIINFEKL (BioLegend: cat. no. 141605, 1:100), FITC anti-mouse H-2Kd bound to SIINFEKL (Beyotime: cat. no. AC1183, 1:100), APC anti-mouse H-2Kd bound to SIINFEKL (Beyotime: cat. no. AC0992, 1:100), PE anti-mouse H-2Kb bound to SIINFEKL (BioLegend: cat. no. 141603, 1:100), FITC anti-mouse CD103 (BioLegend: cat. no. 121407, 1:100), PE anti-mouse CD103 (BioLegend: cat. no. 121405, 1:100), FITC anti-mouse CD172a (SIPRα) (BioLegend: cat. no. 144006, 1:100), PerCP-Cy5.5 anti-mouse XCR1 (BioLegend: cat. no. 148207, 1:100), BV605 anti-mouse XCR1 (BioLegend: cat. no. 148222, 1:100), PerCP-Cy5.5 anti-mouse CD63 (LAMP3) (BioLegend: cat. no. 143912, 1:100), PE-Cy7 anti-mouse Ly-6C (eBioscience: cat. no. 25-5932-80, 1:100), FITC anti-mouse CD19 (BioLegend: cat. no. 115505, 1:100), PerCP-Cy5.5 anti-mouse CD3ε (Tonbo Biosciences: cat. no. 65-0031-u100, 1:100), APC anti-mouse CD3ε (eBioscience: cat. no. 17-0037-42, 1:100), PE anti-mouse CD8α (BioLegend: cat. no. 100707, 1:100), FITC anti-mouse CD8α (Tonbo Biosciences: cat. no. 35-0081-u100, 1:100), PerCP-Cy5.5 anti-mouse CD8α (BioLegend: cat. no. 100733, 1:100), FITC anti-mouse CD4 (Tonbo Biosciences: cat. no. 35-0041-u100, 1:100), PE anti-mouse CD4 (BioLegend: cat. no.100511, 1:100), PerCP-Cy5.5 anti-mouse CD4 (BioLegend: cat. no. 100433, 1:100), PE anti-mouse H-2Kb OVA Tetramer-SIINFEKL (MBL Life Science: cat. no. TS-5001-1C, 1:100), PE anti-mouse IFN-γ (Tonbo Biosciences: cat. no. 50-7311-u025, 1:100), FITC anti-mouse IFN-γ (BioLegend: cat. no. 505805, 1:100), BV421 anti-mouse IFN-γ (BioLegend: cat. no. 505829, 1:100), PE-Cy7 anti-mouse Granzyme B (eBioscience: cat. no. 25-8898-80, 1:100), PE anti-mouse IL-4 (Tonbo Biosciences: cat. no. 50-7041-u025, 1:100), PE anti-mouse IL-17A (eBioscience: cat. no. 12-7177-81, 1:100), PE anti-mouse Foxp3 (Tonbo Biosciences: cat. no. 50-5773-u025, 1:100), FITC anti-mouse Foxp3 (eBioscience: cat. no. 11-5773-82, 1:100), APC anti-mouse CD279 (BioLegend: cat. no. 109111, 1:100), APC anti-mouse CD366 (eBioscience: cat. no. 17-5870-82, 1:100), APC anti-mouse CD223 (eBioscience: cat. no. 17-2231-82, 1:100), PE anti-mouse CD44 (Tonbo Biosciences: cat. no. 50-0441-u025, 1:100), PE-Cy7 anti-mouse CD62L (eBioscience: cat. no. 25-0621-81, 1:100), FITC anti-mouse CD49b (BioLegend: cat. no. 103503, 1:100), APC anti-mouse CD49b (BioLegend: cat. no. 103515, 1:100), PE anti-mouse NK1.1 (BioLegend: cat. no. 156503, 1:100), PE-anti-mouse F4/80 (BioLegend: cat. no. 111704, 1:100), PE-Cy7 anti-mouse CD206 (eBioscience: cat. no. 25-2061-80, 1:100), APC-eFlour 780-anti-Ly-6G/Ly-6C (eBioscience: cat. no. 47-5931-80, 1:100), Alexa Flour 700-anti-F4/80 (eBioscience: cat. no. 56-4801-80, 1:100), PE-Cy7-anti-CD326 (EpCAM) (eBioscience: cat. no. 25-5791-80, 1:100), PE-anti-CD88 (BioLegend: cat. no. 135805, 1:100), Pacific Blue™ anti-mouse Siglec H (BioLegend: cat. no. 129609, 1:100) and APC anti-mouse CD370 (BioLegend: cat. no. 143505, 1:100). Cells from

human and humanized NSG mice were stained with the following antibodies: APC anti-human CD45 (eBioscience: cat. no. 17-0459-42, 1:100), PerCP-Cy5.5 anti-human CD3 (BioLegend: cat. no. 317336, 1:100), FITC anti-human CD8α (eBioscience: cat. no. 11-0088-42, 1:100), FITC anti-human CD4 (eBioscience: cat. no. 11-0049-42, 1:100), PE anti-human IFN-γ (eBioscience: cat. no. 12-7319-42, 1:100), PE-Cy7 anti-human Granzyme B (BioLegend: cat. no. 372214, 1:100), PE anti-human Foxp3 (eBioscience: cat. no. 12-4776-42, 1:100), PE anti-human IL-4 (eBioscience: cat. no. 12-7049-42, 1:100), PE anti-human IL-17A (eBioscience: cat. no. 12-7179-42, 1:100), FITC anti-human CD56 (eBioscience: cat. no. 11-0566-42, 1:100), PerCP-eFlour 710 anti-human CD11b (eBioscience: cat. no. 46-0118-42, 1:100), PE anti-human CD68 (eBioscience: cat. no. 12-0689-42, 1:100), FITC anti-human CD86 (eBioscience: cat. no. 53-0869-42, 1:100), PE-Cy7 anti-human CD206 (eBioscience: cat. no. 25-2069-42, 1:100), PerCP-eFlour 710 anti-human CD11c (eBioscience: cat. no. 46-0116-42, 1:100), APC anti-human CD1c (eBioscience: cat. no. 17-0015-42, 1:100), PE anti-human CD141 (BioLegend: cat. no. 344104, 1:100), FITC anti-human CD123 (eBioscience: cat. no. 11-1239-42), PE anti-human CD40 (BioLegend: cat. no. 334308, 1:100), PE anti-human CD80 (BioLegend: cat. no. 305208, 1:100), APC anti-human CD83 (BioLegend: cat. no. 305312, 1:100) and PE-Cy7 anti-human HLA-DR (BioLegend: cat. no. 327018, 1:100).

Western blot analysis:

The primary antibody was against CCR2 (Abcam: cat. no. ab273050, 1:1000), OPA1 (proteintech: cat. no. 27733-1-AP, 1:1000), OPA1 (Affinity: cat. no. DF8587, 1:1000), ATP5A1 (proteintech: 66037-1-Ig, 1:1000), UQCRC2 (proteintech: 14742-1-AP, 1:1000), SDHB (proteintech: 10620-1-AP, 1:1000), NDUFB8 (proteintech: 67690-1-Ig, 1:1000), IRAK1 (Cell Signaling Technology: 4504S, 1:1000), NF-κB p65 (Cell Signaling Technology: 8242T, 1:1000), MAPK p38 (Cell Signaling Technology: 8690T, 1:1000), MyD88 (Cell Signaling Technology: 4283S, 1:1000), DRP1 (proteintech: 81561-1-RR, 1:1000), Phospho-DRP1S616 (Abclonal: AP1573, 1:1000), Phospho-DRP1S637 (Affinity: DF2980, 1:1000), MFN1 (Affinity: DF7543, 1:1000), MFN2 (Abclonal: A19678, 1:1000), Phospho-mTORS2448 (proteintech: 80596-1-RR, 1:1000), Phospho-AKT1S473 (proteintech: 80462-1-RR, 1:1000), HIF-1α (Affinity: AF1009, 1:1000), PERK (Affinity: AF5304, 1:1000), Phospho-eIF2αS51 (Abclonal: AP0692, 1:1000), eIF2α (Abclonal: A21221, 1:1000), Phospho-IRE1S724 (Abclonal: AP1442, 1:1000), XBP-1S (proteintech: 83959-5-RR, 1:1000), XBP-1U (proteintech: 25997-1-AP, 1:1000), Cleaved Caspase-3 (Cell Signaling Technology: 9661T, 1:1000), Caspase 7 (Cell Signaling Technology: 9492T, 1:1000), β-actin (Cell Signaling Technology: 13E5, 1:1000) and GAPDH (Cell Signaling Technology: 14C10, 1:1000) antibodies and the secondary antibody was horseradish peroxidase-labelled anti-rabbit IgG (H+L) (Yeasen: cat.no. 33101ES60, 1:1000).

Immunofluorescence staining analysis:

The primary antibody was against CCR2 (Abcam: cat. no. ab273050, 1:100), CCR2 (Affinity: cat. no. DF7507, 1:200), CD31 (Abclonal: cat. no. A0378, 1:100), CCL2/MCP-1 (ABclonal: cat. no. A7277 1:200), CD1c (OriGene: cat. no. TA505411S, 1:200), CD141 (ABclonal: cat. no. A22989PM, 1:200), LAMP3 (ABclonal: cat. no. A2895, 1:200), CD83 (Abcam: cat. no. ab205343, 1:200) and CD11c (Abcam: cat. no. ab254183, 1:200), CD8A (Abclonal: cat. no. A23081, 1:100), fibronectin (Affinity: cat. no. AF5335, 1:100), F4/80 (Abcam: cat. no. ab6640, 1:100), 647-conjugated DRP1 (C-terminal) (proteintech: cat. no. CL647-12957, 1:100) and the related secondary antibodies were Multi-rAb CoraLite Plus 555-Goat Anti-Rabbit Recombinant Secondary Antibody (H+L) (proteintech: cat. no. RGAR003, 1:200), Alexa Fluor 488-labelled goat anti-rabbit IgG (H+L) (Yeasen: cat. no. 33106ES60, 1:200) and Alexa Fluor 594-labelled donkey anti-rabbit IgG (H+L) (Yeasen: cat. no. 34212ES60, 1:200).

Tissue microarrays analysis:

Tissue microarrays (TMAs) were purchased from Yanteng Biotechnology Co., Ltd. (Shanghai, China) and Wknowbio Technology Co., Ltd. (Shanghai, China). Breast carcinoma TMAs (AF-BrcSur2201: array point diameter, 1.5 mm, containing 80 breast carcinomas; BRC1603: array point diameter, 1.5 mm, containing 163 breast carcinomas), Melanoma TMAs (K063Me01: array point diameter, 1.5 mm, containing 61 melanoma samples; K983501: array point diameter, 1.5 mm, containing 49 melanoma samples) and Colorectal cancer TMAs (ZM6.27H-1: array point diameter, 1.5 mm, containing 78 colorectal cancer samples; ZM6.27H-2: array point diameter, 1.5 mm, containing 71 colorectal cancer samples) were applied to measure the expression of CD1c, CD141, LAMP3, CD83 and CD11c. The samples were incubated with primary antibodies against CD1c, CD141, LAMP3, CD83 and CD11c for immumohistochemical and immunofluorescent staining. They were imaged with slides scanner (Pannoramic DESK, 3D HISTECH, Hungary) and analyzed via Image J software.

Validation

All antibodies were supplied by vendors, BioLegend, eBioscience, Tonbobio, Abcam, Affinity, Cell Signaling Technology, Bioss, Yeasen, Servicebio, and SimuwuBio. Their relevant citations and antibody profiles were validated. For flow cytometry analysis, the fluorescence-conjugated antibodies were purchased from ebioscience, BioLegend or Tonbo Biosciences to label the different cell markers. Fixable Viability Dye eFluor 455 (eBioscience: cat. no. 65-0868-14, 1:100) was used to recognize and exclude the dead cell populations. Anti-mouse CD16/32 (eBioscience: cat. no. 14-0161-82) antibody was used for Fc blockade. Cells were respectively stained with the following antibodies: PE anti-mouse CCR2 (BioLegend: cat. no. 150609, 1:100), APC-Cy7 anti-mouse CD45 (BioLegend: cat. no. 103116, 1:100), APC anti-mouse CD45 (eBioscience: cat. no. 17-0451-82, 1:100), Alexa Flour 700 anti-mouse CD11c (BioLegend: cat. no. 117319, 1:100), PE anti-mouse CD11c (BioLegend: cat. no. 117307, 1:100), APC anti-mouse CD11c (Tonbo Biosciences, cat. no. 20-0114-u025, 1:100), PerCP-Cy5.5 anti-mouse CD11b (Tonbo Biosciences, cat. no. 65-0112-u100, 1:100), FITC anti-mouse CD11b (eBioscience: cat. no. 11-0112-82, 1:100), Alexa Flour 700 anti-mouse CD11b (eBioscience: cat. no. 56-0112-80, 1:100), PE anti-mouse CD80 (Tonbo Biosciences, cat. no. 50-0801-u025, 1:100), PE-Cy7 anti-mouse CD80 (Tonbo Biosciences, cat. no. 60-0801-u025, 1:100), APC-Cy7 anti-mouse CD86 (BioLegend: cat. no. 105029, 1:100), FITC anti-mouse CD86 (Tonbo Biosciences, cat. no. 35-0862-u025, 1:100), APC-anti-CD86 (eBioscience: cat. no. 17-0862-82, 1:100), PE anti-mouse MHC I (eBioscience: cat. no. 12-5958-82, 1:100), FITC anti-mouse MHC I (eBioscience: cat. no. 11-5958-80, 1:100), APC anti-mouse MHC-I (eBioscience: cat. no. 12-5958-82, 1:100), FITC anti-mouse MHC II (Tonbo Biosciences, cat. no. 35-5321-u025, 1:100), PE anti-mouse MHC II (Tonbo Biosciences, cat. no. 50-5321-u025, 1:100), APC anti-mouse CD83 (BioLegend: cat. no. 121509, 1:100), PE anti-mouse CD40 (eBioscience: cat. no. 12-0401-81, 1:100), APC anti-mouse H-2Kb bound to SIINFEKL (BioLegend: cat. no. 141605, 1:100), PE anti-mouse CD103 (BioLegend: cat. no. 121405, 1:100), PE-Cy7 anti-mouse Ly-6C (eBioscience: cat. no. 25-5932-80, 1:100), PerCP-Cy5.5 anti-mouse CD3ε (Tonbo Biosciences: cat. no. 65-0031-u100, 1:100), APC anti-mouse CD3ε (eBioscience: cat. no. 17-0037-42, 1:100), PE anti-mouse CD8α (BioLegend: cat. no. 100707, 1:100), FITC anti-mouse CD8α (Tonbo Biosciences: cat. no. 35-0081-u100, 1:100), FITC anti-mouse CD4 (Tonbo Biosciences: cat. no. 35-0041-u100, 1:100), PE anti-mouse H-2Kb OVA Tetramer-SIINFEKL (MBL Life Science: cat. no. TS-5001-1C, 1:100), PE anti-mouse IFN-γ (Tonbo Biosciences: cat. no. 50-7311-u025, 1:100), FITC anti-mouse IFN-γ (BioLegend: cat. no. 505805, 1:100), BV421 anti-mouse IFN-γ (BioLegend: cat. no. 505829, 1:100), PE-Cy7 anti-mouse Granzyme B (eBioscience: cat. no. 25-8898-80, 1:100), PE anti-mouse IL-4 (Tonbo Biosciences: cat. no. 50-7041-u025, 1:100), PE anti-mouse IL-17A (eBioscience: cat. no. 12-7177-81, 1:100), PE anti-mouse Foxp3 (Tonbo Biosciences: cat. no. 50-5773-u025, 1:100), APC anti-mouse CD279 (BioLegend: cat. no. 109111, 1:100), APC anti-mouse CD366 (eBioscience: cat. no. 17-5870-82, 1:100), APC anti-mouse CD223 (eBioscience: cat. no. 17-2231-82, 1:100), PE anti-mouse CD44 (Tonbo Biosciences: cat. no. 50-0441-u025, 1:100), PE-Cy7 anti-mouse CD62L (eBioscience: cat. no. 25-0621-81, 1:100) PE-anti-mouse F4/80 (BioLegend: cat. no. 111704, 1:100) and PE-Cy7 anti-mouse CD206 (eBioscience: cat. no. 25-2061-80, 1:100). Cells from human and humanized NSG mice were stained with the following antibodies: APC anti-human CD45 (eBioscience: cat. no. 17-0459-42, 1:100), PerCP-Cy5.5 anti-human CD3 (BioLegend: cat.

no. 317336, 1:100), FITC anti-human CD8α (eBioscience: cat. no. 11-0088-42, 1:100), FITC anti-human CD4 (eBioscience: cat. no. 11-0049-42, 1:100), PE anti-human IFN-γ (eBioscience: cat. no. 12-7319-42, 1:100), PE-Cy7 anti-human Granzyme B (BioLegend: cat. no. 372214, 1:100), PE anti-human Foxp3 (eBioscience: cat. no. 12-4776-42, 1:100), PE anti-human IL-4 (eBioscience: cat. no. 12-7049-42, 1:100), PE anti-human IL-17A (eBioscience: cat. no. 12-7179-42, 1:100), FITC anti-human CD56 (eBioscience: cat. no. 11-0566-42, 1:100), PerCP-eFlour 710 anti-human CD11b (eBioscience: cat. no. 46-0118-42, 1:100), PE anti-human CD68 (eBioscience: cat. no. 12-0689-42, 1:100), FITC anti-human CD86 (eBioscience: cat. no. 53-0869-42, 1:100), PE-Cy7 anti-human CD206 (eBioscience: cat. no. 25-2069-42, 1:100), PerCP-eFlour 710 anti-human CD11c (eBioscience: cat. no. 46-0116-42, 1:100), APC anti-human CD1c (eBioscience: cat. no. 17-0015-42, 1:100), PE anti-human CD141 (BioLegend: cat. no. 344104, 1:100), FITC anti-human CD123 (eBioscience: cat. no. 11-1239-42), PE anti-human CD40 (BioLegend: cat. no. 334308, 1:100), PE anti-human CD80 (BioLegend: cat. no. 305208, 1:100), APC anti-human CD83 (BioLegend: cat. no. 305312, 1:100) and PE-Cy7 anti-human HLA-DR (BioLegend: cat. no. 327018, 1:100). For western blot (Bio-Rad, USA) detection, the primary antibody was against CCR2 (Abcam: cat. no. ab273050, 1:1000), OPA1 (proteintech: cat. no. 27733-1-AP, 1:1000), ATP5A1 (proteintech: 66037-1-Ig, 1:1000), UQCRC2 (proteintech: 14742-1-AP, 1:1000), SDHB (proteintech: 10620-1-AP, 1:1000), NDUFB8 (proteintech: 67690-1-Ig, 1:1000), IRAK1 (Cell Signaling Technology: 4504S, 1:1000), NF-κB p65 (Cell Signaling Technology: 8242T, 1:1000), MAPK p38 (Cell Signaling Technology: 8690T, 1:1000), MyD88 (Cell Signaling Technology: 4283S, 1:1000), β-actin (Cell Signaling Technology: 13E5, 1:1000) and GAPDH (Cell Signaling Technology: 14C10, 1:1000) antibodies and the secondary antibody was horseradish peroxidase-labelled anti-rabbit IgG (H+L) (Yeasen: cat.no. 33101ES60, 1:1000). For immunofluorescence (IF) and immumohistochemical staining, the primary antibody was against CCR2 (Abcam: cat. no. ab273050, 1:100), CCR2 (Affinity: cat. no. DF7507, 1:200), CD31 (ABclonal: cat. no. A0378, 1:100), CCL2/MCP-1 (ABclonal: cat. no. A7277 1:200), IL-12B (ABclonal: cat. no. A24262, 1:200), CD83 (Abcam: cat. no. ab205343, 1:200) and CD11c (Abcam: cat. no. ab254183, 1:200), CD8A (ABclonal: cat. no. A23081, 1:100), fibronectin (Affinity: cat. no. AF5335, 1:100) and the related secondary antibodies were Alexa Fluor 488-labelled goat anti-rabbit IgG (H+L) (Yeasen: cat. no. 33106ES60, 1:200) and Alexa Fluor 594-labelled donkey anti-rabbit IgG (H+L) (Yeasen: cat. no. 34212ES60, 1:200).

Tissue Microarrays
Tissue microarrays (TMAs) were purchased from Yanteng Biotechnology Co., Ltd. (Shanghai, China) and Wknowbio Technology Co., Ltd. (Shanghai, China). Breast carcinoma with matched or unmatched breast TMAs (AF-BrcSur2201: array point diameter, 1.5 mm, containing 80 breast carcinomas; BRC1603: array point diameter, 1.5 mm, containing 163 breast carcinomas) were applied to measure the expression of CCL2/MCP-1, IL-12B, CD83 and CD11c. The samples were incubated with primary antibodies against CCL2/MCP-1, IL-12B, CD83 and CD11c for immumohistochemical and immunofluorescent staining. They were imaged with slides scanner (Pannoramic DESK, 3D HISTECH, Hungary) and analyzed via Image J software.

Eukaryotic cell lines

Policy information about cell lines and Sex and Gender in Research

Cell line source(s)	Cell line sources were provided under "Method: cell culture" section. DC2.4, HCT116 and B16-OVA cell lines were purchased from Jinyuan Biotechnology Co., Ltd. (Shanghai, China). 4T1-OVA cell line was purchased from Fuheng Biotechnology Co., Ltd. (Shanghai, China). MDA-MB-231, CT26-Luc, 4T1, 4T1-Luc and 4T1-GFP cell lines were purchased from the Cell Bank of Shanghai, Chinese Academy of Sciences. The cells were tested mycoplasma negative before use. All cell lines were cultured in RPMI 1640 medium containing 10% fetal bovine serum (FBS, Gibco, USA), 2.5 g/L of glucose, 0.11 g/L of sodium pyruvate and 1% penicillin/streptomycin at 37°C in a humidified atmosphere of 95% air and 5% CO2. Cells were stored at -80 °C in CELLSAVING freezing medium (NCM Biotech, Suzhou, China). To construct the hypoxic environment, cells were put into the hypoxia cell incubator chamber (StemCell, Canada). In brief, 0.1% O2 gas, 5% CO2, and N2 (as a balance) were purged into the chamber at 20 L/min for 5 min, then the inlet and outlet ports were sealed. They were put into the incubator at 37 °C. Murine splenocytes were extracted from spleen of female BALB/c mice.
Authentication	The cell lines were identified by the manufacturers.
Mycoplasma contamination	Cell lines used in this work were tested and without mycoplasma contamination.
Commonly misidentified lines (See ICLAC register)	This is not a problem associated with these cell lines.

Animals and other research organisms

Policy information about studies involving animals; ARRIVE guidelines recommended for reporting animal research, and Sex and Gender in Research

Laboratory animals	Female Balb/c mice (20-22 g) and female C57BL/6 (CD45.2) mice (20-22g) were purchased from Shanghai Experimental Animal Center. Female C57BL/6 (CD45.1) mice, female M-NSG (NOD-PrkdcscidIl2rgem1/Smoc) mice, female Ccl2-/- mice, female Batf3-/- mice and female Rag3-/- mice were purchased from Shanghai Model Organisms Center, Inc. Mice were housed in groups of 5 mice per cage, maintained at a temperature of ~25 °C in a humidity-controlled environment with a 12 h light/dark cycle.
Wild animals	No wild animals were applied.
Reporting on sex	Female animals were collected and used for breast cancer models construction.
Field-collected samples	No field-collected samples were collected.
Ethics oversight	All animals were bred in the animal faculty of Shanghai Institute of Materia Medica, Chinese Academy of Sciences (CAS, Shanghai, China), according to the raising principles approved by the Institutional Animal Care and Use Committee.

Note that full information on the approval of the study protocol must also be provided in the manuscript.

Dual use research of concern

Policy information about dual use research of concern

Hazards

Could the accidental, deliberate or reckless misuse of agents or technologies generated in the work, or the application of information presented in the manuscript, pose a threat to:

No	Yes	
☐	☐	Public health
☐	☐	National security
☐	☐	Crops and/or livestock
☐	☐	Ecosystems
☐	☐	Any other significant area

Experiments of concern

Does the work involve any of these experiments of concern:

No	Yes	
☐	☐	Demonstrate how to render a vaccine ineffective
☐	☐	Confer resistance to therapeutically useful antibiotics or antiviral agents
☐	☐	Enhance the virulence of a pathogen or render a nonpathogen virulent
☐	☐	Increase transmissibility of a pathogen
☐	☐	Alter the host range of a pathogen
☐	☐	Enable evasion of diagnostic/detection modalities
☐	☐	Enable the weaponization of a biological agent or toxin
☐	☐	Any other potentially harmful combination of experiments and agents

Plants

Seed stocks: The algae (GY-D12 Chlorella pyrenoidosa) were purchased from Guangyu Biological Technology Co., Ltd. (Shanghai, China).

Novel plant genotypes: *Describe the methods by which all novel plant genotypes were produced. This includes those generated by transgenic approaches, gene editing, chemical/radiation-based mutagenesis and hybridization. For transgenic lines, describe the transformation method, the number of independent lines analyzed and the generation upon which experiments were performed. For gene-edited lines, describe the editor used, the endogenous sequence targeted for editing, the targeting guide RNA sequence (if applicable) and how the editor was applied.*

Authentication: *Describe any authentication procedures for each seed stock used or novel genotype generated. Describe any experiments used to assess the effect of a mutation and, where applicable, how potential secondary effects (e.g. second site T-DNA insertions, mosiacism, off-target gene editing) were examined.*

Flow Cytometry

Plots

Confirm that:

☒ The axis labels state the marker and fluorochrome used (e.g. CD4-FITC).

☒ The axis scales are clearly visible. Include numbers along axes only for bottom left plot of group (a 'group' is an analysis of identical markers).

☒ All plots are contour plots with outliers or pseudocolor plots.

☒ A numerical value for number of cells or percentage (with statistics) is provided.

Methodology

Sample preparation: Fresh tumours were cut and digested in basal RPMI 1640 media (BasalMedia, L220KJ) with supplement of DNAase (Solarbio: cat.no. D8071, 0.1 mg/mL), collagenase IV (Solarbio: cat.no. C8160, 0.2 mg/mL), and hyaluronidase (Solarbio: H8030, 0.2 mg/mL,) at 37 °C for 1 h. Cells were filtered with 70 µm of cell strainer and collected.
Besides, spleens were grinded with syringe handle, and single-cell suspensions were collected.

Instrument	BD Fortessa or FACSCalibur flow cytometers (USA)
Software	Data collection: BD LSRFortessa Cell Analyzer (BD Biosciences)<br>Data analysis: FlowJo software package (version 10.6.2; BD, USA).
Cell population abundance	Spleen tissues were grinded with syringe handle, and filtered with cell strainers (70 µm, Falcon, USA). Cells were collected (400 g, 10 min), and removed the red blood cells with lysis buffer (Yeasen: cat.no. 40401ES76) to obtain the purified splenocytes.<br>Human PBMCs were isolated using human peripheral blood monocyte isolation kit (TBD2011H05, tbdscience).<br>For immunological efficacy analysis, tumour cells were not sorted.
Gating strategy	The sequential gating strategy was performed: first FSC-A vs FSC-H and SSC-A vs SSC-H axis were plotted to label the singlet population; second Fixable viability dye (FVD-eFluor 450) were used to mark the dead cells and classify the live cells. Gating was then based on positive level. Gating strategies were shown in Supplementary Data.

[x] Tick this box to confirm that a figure exemplifying the gating strategy is provided in the Supplementary Information.
