# Supplementary material for: A plant vesicle-dendritic cell chimera for enhancing cancer immunotherapy
Source: Nat Commun. 2026 May 28;17:6944. doi: 10.1038/s41467-026-73788-5 (PMC13388912; doi:10.1038/s41467-026-73788-5)
Supplement: Supplementary file 5 — Transparent Peer Review file [file 41467_2026_73788_MOESM5_ESM.pdf]

nature portfolio

Peer Review File

A plant vesicle-dendritic cell chimera for enhancing cancer immunotherapy

Corresponding Author: Professor Yaping Li

This manuscript has been previously reviewed at another journal. This document only contains information relating to versions considered at Nature Communications.

Parts of this Peer Review File have been redacted as indicated to remove third-party material.

This file contains all reviewer reports in order by version, followed by all author rebuttals in order by version.

Version 0:

Reviewer comments:

Reviewer #1

(Remarks to the Author)
The authors have sufficiently addressed my concerns in two rounds of revisions. I believe the revised version of this manuscript is of high standard and puts forward an exciting out of the box concept, that is worthy of publication in this journal. I have no further comments

Reviewer #2

(Remarks to the Author)
This study shows some startling results that introduction of algal chloroplasts into DCs and activating a photosynthetic machinery can activate DCs and exert an antitumor effect. The authors have addressed the questions that this reviewer had asked. However, this reviewer has some further queries

1. The authors show that mitochondrial mass in DCs drops rapidly within 4h in hypoxia, but is restored on shining light on the ANV-DC construct. The authors hypothesize that this is due to mitochondrial fission in hypoxia and fusion in the presence of oxygen generated during photosynthesis. Can the authors show that at 54h, where they claim recovery with ANVs, the mitochondria undergoing fission hasn't entered mitophagy, in which case it will require mitochondrial biogenesis to restore mitochondrial mass.

2. Why does ANVs decrease fibronectin levels in tumor? That suggests tumor remodeling, which is not supported by the mechanism that the authors propose.

Reviewer #3

(Remarks to the Author)
In this revision round, the authors have addressed my concerns much more thoroughly and provided solid data to support their conclusions, substantially improving the overall quality of the manuscript.

To clarify comment 2: Although CLL are still used by several groups, this does not necessarily make them an optimal strategy. CLL is often considered a “dirty” approach in the macrophage field because it is taken up by multiple phagocytic cell types, leading to apoptosis not only of macrophages, but also of monocytes and DCs (mainly cDC2s and DC3s). Therefore, it is not a macrophage-specific depletion method. Moreover, it has been shown that some CLL-mediated effects can occur independently of macrophages and instead be driven by polymorphonuclear cells (for example Culemann et al., J Exp Med, 2023). Given that CLL can also induce NETosis and lacks specificity for macrophages, it represents a less suitable depletion strategy compared to the more selective approach now implemented in the revised manuscript.

Remaining major comment:

Previous comment 3: The gating strategy remains suboptimal. The approach referenced by the authors (Nature 645, 244–253 (2025)) is only valid for lymph nodes (and to some extent spleen). In other organs, DCs (or the distingtion between resident and migratory DCs) cannot be reliably defined using this marker combination. To identify migratory DCs in tumors, markers such as Fcn1 or CCR7 should be included. Even then, these markers indicate migratory potential rather than actual migration.
The persistent large double-negative fraction within the $CD11b^{-}CD103^{-}$ gate may result from incomplete exclusion of monocyte-derived cells. In the $GR\text{-}1^{+}$ gate (which strongly detects Ly6G and to a lesser extent Ly6C), primarily neutrophils, and only partially monocytes, are excluded. Monocytes may therefore still be present in subsequent gates. If the authors do not intend to revise their gating strategy, I suggest referring to these populations as “DC-like cells,” in which case the conclusions can be kept as such.
Importantly, Lamp3 expression differs substantially between mouse and human. In humans, LAMP3 marks $CCR7^{+}$ mature DCs. In mice, however, Lamp3 is neither DC-specific nor expressed on mature DCs. Therefore, the Lamp3 gates shown (which are evidently negative) are not informative and should be removed

Minor comments
- There are still many grammatical/spelling errors: examples: line 24 (“The” TME), 88 (“The” TME), 106 (tumour “progression”), line 538 to be rewritten, line 553 (to “investigate”), line 1700 and 1702 (“adoptive”),
- Figure S10: under the 3rd gate: what is “flexible dye”. Perhaps the authors meant “Fixable” live-dead dye?
- Line 159: how cn mitochondrial morphology be profiled at the transcriptomic level?

Version 1:

Reviewer comments:

Reviewer #2

(Remarks to the Author)
The authors have addressed my queries. I have no further queries.

Reviewer #3

(Remarks to the Author)
In this revised version, the authors have incorporated my comments, enhancing the overall accuracy of the manuscript.

Open Access This Peer Review File is licensed under a Creative Commons Attribution 4.0 International License, which permits use, sharing, adaptation, distribution and reproduction in any medium or format, as long as you give appropriate credit to the original author(s) and the source, provide a link to the Creative Commons license, and indicate if changes were made.

In cases where reviewers are anonymous, credit should be given to 'Anonymous Referee' and the source.
The images or other third party material in this Peer Review File are included in the article's Creative Commons license, unless indicated otherwise in a credit line to the material. If material is not included in the article's Creative Commons license and your intended use is not permitted by statutory regulation or exceeds the permitted use, you will need to obtain permission directly from the copyright holder.
To view a copy of this license, visit https://creativecommons.org/licenses/by/4.0/

Response to reviewers

Reviewer #1:

The authors have sufficiently addressed my concerns in two rounds of revisions. I believe the revised version of this manuscript is of high standard and puts forward an exciting out of the box concept, that is worthy of publication in this journal. I have no further comments

Response: We would like to thank you for your professional feedback, constructive comments and valuable suggestions on our manuscript.

Reviewer #2:

This study shows some startling results that introduction of algal chloroplasts into DCs and activating a photosynthetic machinery can activate DCs and exert an antitumor effect. The authors have addressed the questions that this reviewer had asked. However, this reviewer has some further queries:

1. The authors show that mitochondrial mass in DCs drops rapidly within 4h in hypoxia, but is restored on shining light on the ANV-DC construct. The authors hypothesize that this is due to mitochondrial fission in hypoxia and fusion in the presence of oxygen generated during photosynthesis. Can the authors show that at 54h, where they claim recovery with ANVs, the mitochondria undergoing fission hasn't entered mitophagy, in which case it will require mitochondrial biogenesis to restore mitochondrial mass.

Response: Thank you for this insightful comment. Mitophagy is a core mechanism for maintaining cellular homeostasis and energy metabolism, ensuring cell function by selectively eliminating damaged mitochondria[1]. We first analyzed the mitophagic activity of CCR2-DC-ANV (without light) in a simulated hypoxic tumor microenvironment in vitro. The results showed that the proportion of mitophagic-positive cells was only approximately 10% after 54 h of culture, even though increased with prolonged time but remained at a low overall level.

Following light treatment at 24 h, the mitophagic activity recovered to the normal physiological state. Further observation of CCR2-DC-ANV at 54 h post-culture (with laser irradiation at 24 h) revealed that mitochondria exhibited a fragmented morphology, with only a small number of autophagosomes observed. After irradiation at 54 h, the mitochondrial length and abundance significantly increased, yet the mitophagic activity remained at a low level. These results indicate that irradiation-induced mitochondrial fusion is the primary pathway for mitochondrial quality restoration in CCR2-DC-ANV. Besides, the baseline mitophagic activity of CCR2-DC-ANV under hypoxic tumor microenvironment is low and irradiation has no significant effect on it.

Recent studies (Science 2026(392): eadv6582) have demonstrated that the dynamic balance between mitochondrial fusion and fission in tumor-infiltrating cDC1 is critical for regulating their anti-tumor immunity[2]. Alterations in mitochondrial morphology and functional status can inhibit mitophagy and lysosome-dependent degradation of major histocompatibility complex class I

(MHC-I) and antigens. This finding is consistent with the low mitophagic activity observed in CCR2-DC-ANV in our study.

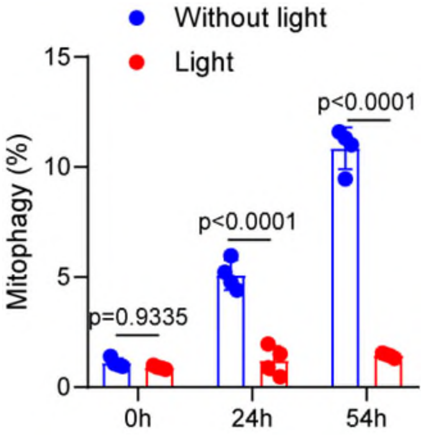

Figure. Percentage of mitophagy in CCR2-DC-ANV at different time points (n = 4).

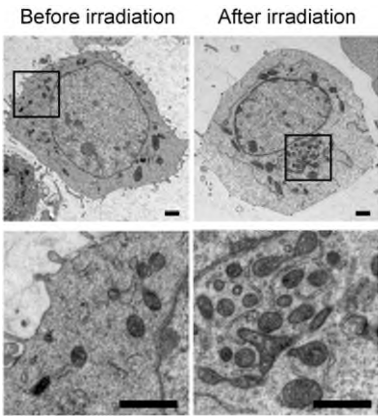

Figure. Transmission electron microscopy images showing the mitochondrial morphology of CCR2-DC-ANV at 54 h (with laser irradiation at 24 h). Scale bar, 1 μm.

2. Why does ANVs decrease fibronectin levels in tumor? That suggests tumor remodeling, which is not supported by the mechanism that the authors propose.

Response: Thanks. Previous studies have indicated that TGF-β1 drove tumour fibrosis via the TGF-β/Smad signaling pathway, activating cancer-associated fibroblasts (CAFs) to overproduce fibronectin[3,4]. It leads to pathological extracellular matrix (ECM) deposition and impaired immune cell infiltration. According to immune-related investigation, CCR2-DC-ANV+L treatment significantly downregulated intratumoural TGF-β1 (Fig. 5u). Based on these results, CCR2-DC-ANV+L treatment enhanced M1-type macrophage proportion and suppressed TGF-β1 expression, which might contribute to angiogenesis and fibronectin

reduction. (**Page 21, Lines 569-578**).

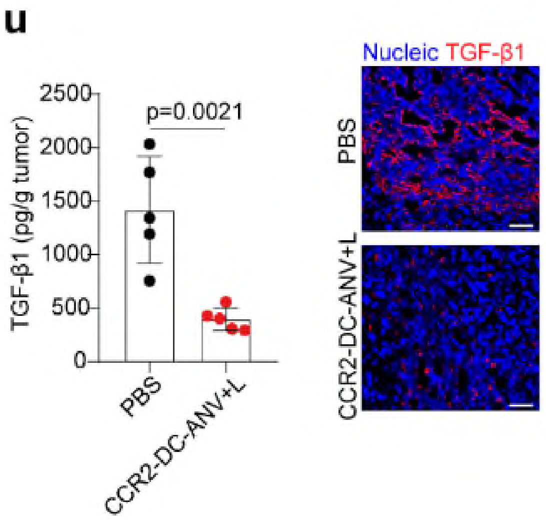

Figure 5u, Quantitative analysis and representative immunofluorescence images of TGF-β1 level (n=5).

1 Picca A. *et al.* Mitophagy in human health, ageing and disease. *Nat Metab* **5**, 2047-2061 (2023).

2 You, Z. *et al.* Mitochondrial metabolism and signaling direct dendritic cell function in antitumor immunity. *Science* **392**, eadv6582 (2026).

3 Zhou, M. *et al.* Nanovesicles loaded with a TGF-β receptor 1 inhibitor overcome immune resistance to potentiate cancer immunotherapy. *Nat Commun* **14**, 3593 (2023).

4 Ren, L. L. *et al.* TGF-β as A Master Regulator of Aging-Associated Tissue Fibrosis. *Aging Dis* **14**, 1633-1650 (2023).

Reviewer #3:

In this revision round, the authors have addressed my concerns much more thoroughly and provided solid data to support their conclusions, substantially improving the overall quality of the manuscript.

Response: We are sincerely grateful for your feedback, which have been instrumental in enhancing the quality and rigor of our manuscript.

To clarify comment 2: Although CLL are still used by several groups, this does not necessarily make them an optimal strategy. CLL is often considered a "dirty" approach in the macrophage field because it is taken up by multiple phagocytic cell types, leading to apoptosis not only of macrophages, but also of monocytes and DCs (mainly cDC2s and DC3s). Therefore, it is not a macrophage-specific depletion method. Moreover, it has been shown that some CLL-mediated effects can occur independently of macrophages and instead be driven by polymorphonuclear cells (for example Culemann et al., J Exp Med, 2023). Given that CLL can also induce NETosis and lacks specificity for macrophages, it represents a less suitable depletion strategy compared to the more selective approach now implemented in the revised manuscript.

Response: Thank you very much for your thoughtful discussion and insightful feedback regarding the use of clodronate liposomes (CLL) in our study. We greatly appreciate the opportunity to address your concerns, which have helped us improve the rigor and specificity of our experimental design.

Remaining major comment:

Previous comment 3: The gating strategy remains suboptimal. The approach referenced by the authors (Nature 645, 244-253 (2025)) is only valid for lymph nodes (and to some extent spleen). In other organs, DCs (or the distingtion between resident and migratory DCs) cannot be reliably defined using this marker combination. To identify migratory DCs in tumors, markers such as Fcn1 or CCR7 should be included. Even then, these markers indicate migratory potential rather than actual migration.

The persistent large double-negative fraction within the CD11b^{-}CD103^{-} gate may result from incomplete exclusion of monocyte-derived cells. In the GR-1^{+} gate (which strongly detects Ly6G and to a lesser extent Ly6C), primarily neutrophils, and only partially monocytes, are

excluded. Monocytes may therefore still be present in subsequent gates. If the authors do not intend to revise their gating strategy, I suggest referring to these populations as “DC-like cells,” in which case the conclusions can be kept as such.

Importantly, Lamp3 expression differs substantially between mouse and human. In humans, LAMP3 marks CCR7^{+} mature DCs. In mice, however, Lamp3 is neither DC-specific nor expressed on mature DCs. Therefore, the Lamp3 gates shown (which are evidently negative) are not informative and should be removed.

Response: Thank you for the comments. In response to your suggestion, we have revised the cell designation in Fig. S2 to ensure the accuracy of characterization. The originally labeled "Mig-cDCs" have been updated to **CD11c^{+}MHC-II^{+} DC-like cells** to more precisely reflect their immunophenotypic features. Additionally, to streamline the dataset and focus on core findings, we have removed LAMP3-associated data from the mouse experiments.

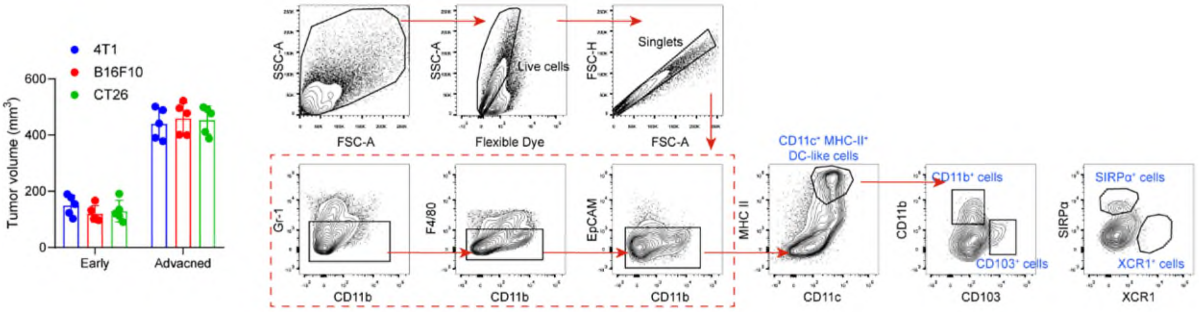

Figure S2. Quantifications of tumour volume at day 7 (early) and 12 (late) and flow cytometric gating strategies to analyze CD11c^{+}MHC-II^{+} cells in tumours.

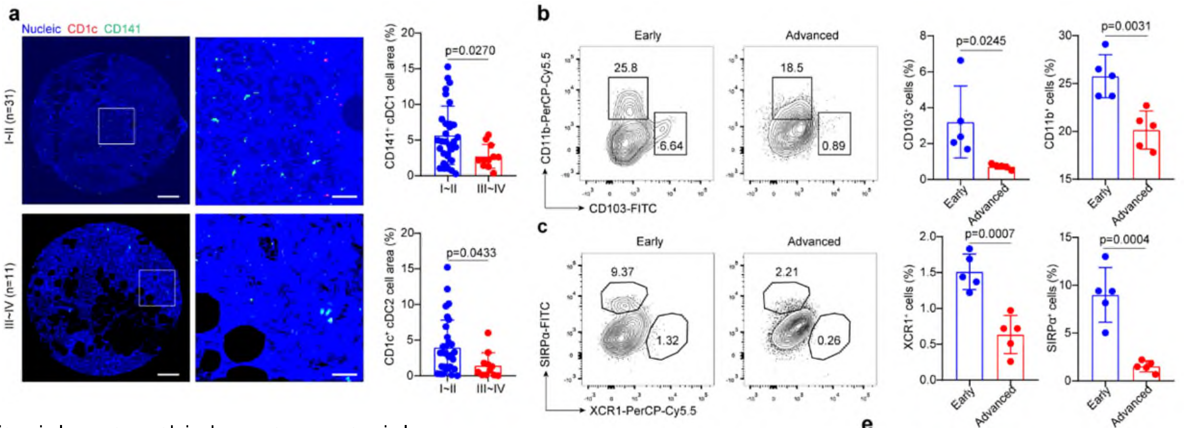

[editorial note: third party material redacted]

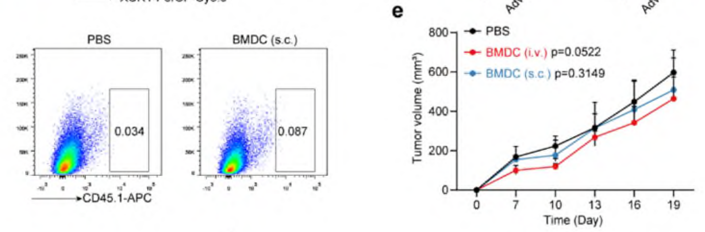

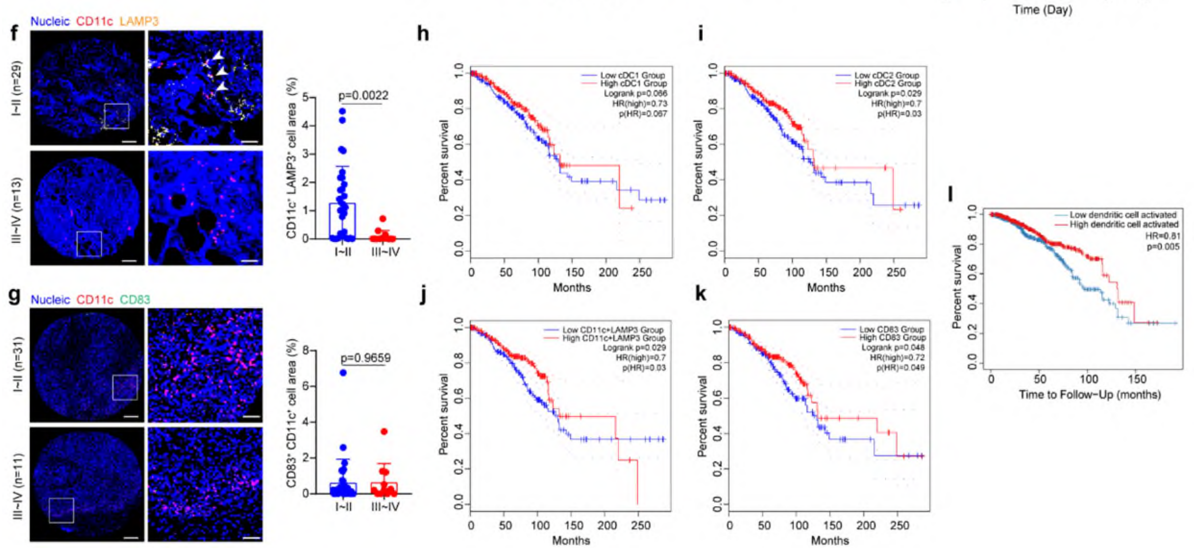

Figure 1. Low infiltration and activation of DCs as tumours progression. b-c, Representative flow cytometry plots and quantification of $CD103^+$ and $CD11b^+$ cells (gated on $CD11c^+MHC\ II^+$ cells) (**b**) or $XCR1^+$ and $SIRP\alpha^+$ cells (gated on $CD11c^+MHC\ II^+$ cells) (**c**) in 4T1 tumour-bearing mice (n=5).

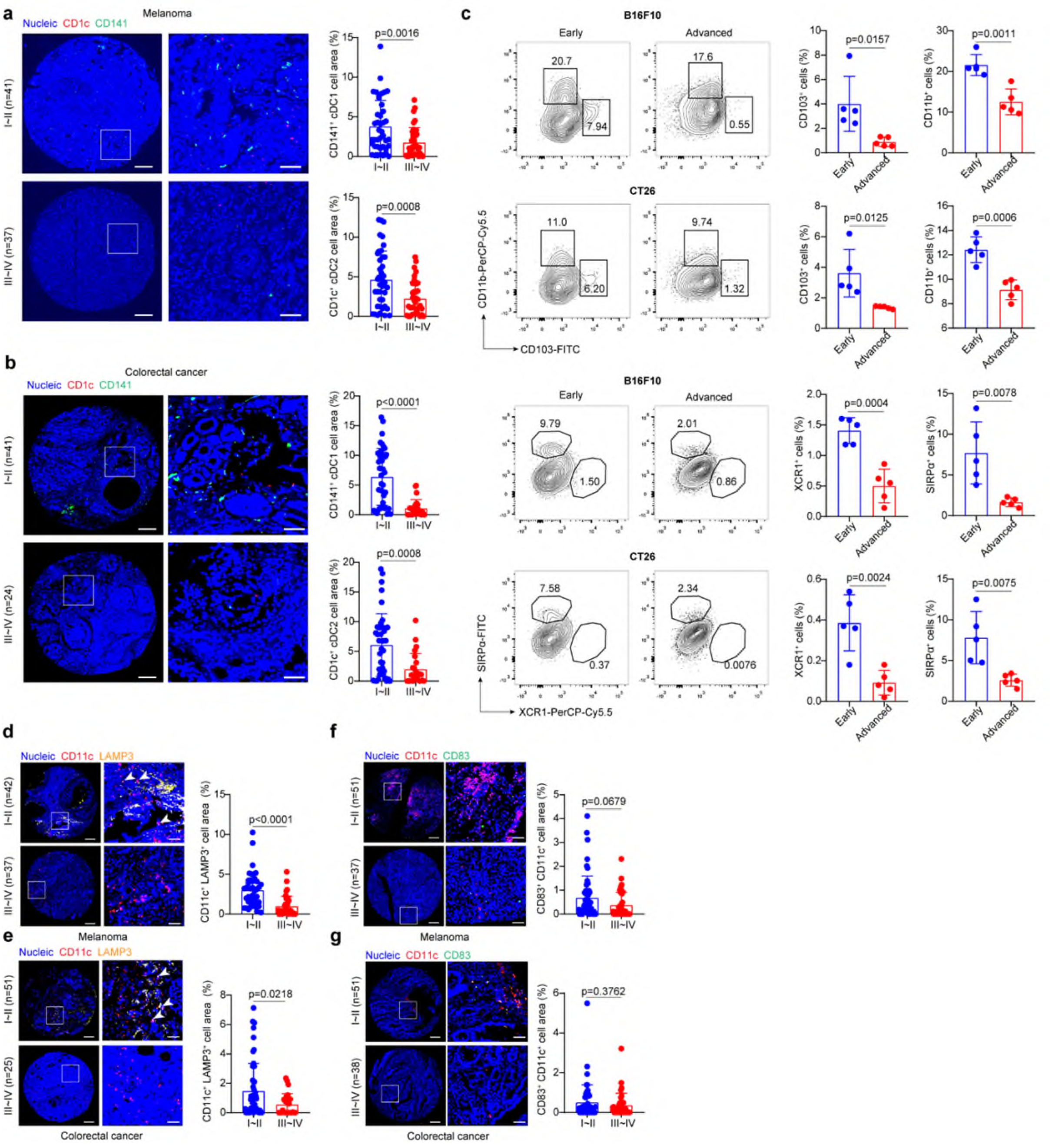

Extended Data Fig 1. Low infiltration and activation of DCs in colorectal cancer and melanoma. c, Representative flow cytometry plots and quantification of $CD103^+$ and $CD11b^+$ cells or $XCR1^+$ and $SIRP\alpha^+$ cells (gated on $CD11c^+$MHC II^+ cells) in B16F10 or CT26 tumour-bearing mice (n=5).

Minor comments

1. There are still many grammatical/spelling errors: examples: line 24 (“The” TME), 88 (“The” TME), 106 (tumour “progression”), line 538 to be rewritten, line 553 (to “investigate”), line 1700 and 1702 (“adoptive”)

Response: Thank the reviewer for pointing out our mistakes in the language. We have corrected the grammatical/spelling errors you mentioned. In addition, we have asked professionals to embellish the language of our articles. For example, we have revised "of" to "in" (Page 2, Line 24), "to" to "for" (Page 2, Line 37), "DC dysfunction" to "the dysfunction of DC" (Page 3, Line 47), "to repair" to "for repairing" (Page 4, Line 76), "progress" to "progression" (Page 51, Line 1695) and rewritten the misunderstanding sentence (Page 4, Line 100-102).

2. Figure S10: under the 3rd gate: what is "flexible dye". Perhaps the authors meant "Fixable" live-dead dye?

Response: Thank you for the comments. In Fig. S10, the term **"flexible dye"** was an inadvertent misnomer; it **denotes** the **"Fixable Viability Dye"** (a reagent commonly employed for labeling live and dead cells). We have corrected this terminology in the revised manuscript to ensure accuracy.

3. Line 159: how can mitochondrial morphology be profiled at the transcriptomic level?

Response: Thank you for the comments. We apologize for the errors in the original manuscript. We employed gene set enrichment analysis (GSEA) for evaluating predefined gene sets associated with mitochondrial function in TADCs. The incorrect terminology previously used has been rectified in the revised version to ensure methodological precision **(Page 7, Line 159)**.
